# Genome-wide RNAi Screen Reveals a Role for Multipass Membrane Proteins in Endosome-to-Golgi Retrieval

**DOI:** 10.1016/j.celrep.2014.10.053

**Published:** 2014-11-20

**Authors:** Sophia Y. Breusegem, Matthew N.J. Seaman

**Affiliations:** 1 Department of Clinical Biochemistry, Cambridge Institute for Medical Research, University of Cambridge, Wellcome Trust/MRC Building, Cambridge Biomedical Campus, Hills Road, Cambridge CB2 0XY, UK

## Abstract

Endosome-to-Golgi retrieval is an essential membrane trafficking pathway required for many important physiological processes and linked to neurodegenerative disease and infection by bacterial and viral pathogens. The prototypical cargo protein for this pathway is the cation-independent mannose 6-phosphate receptor (CIMPR), which delivers lysosomal hydrolases to endosomes. Efficient retrieval of CIMPR to the Golgi requires the retromer complex, but other aspects of the endosome-to-Golgi retrieval pathway are poorly understood. Employing an image-based antibody-uptake assay, we conducted a genome-wide RNAi loss-of-function screen for novel regulators of this trafficking pathway and report ∼90 genes that are required for endosome-to-Golgi retrieval of a CD8-CIMPR reporter protein. Among these regulators of endosome-to-Golgi retrieval are a number of multipass membrane-spanning proteins, a class of proteins often overlooked with respect to a role in membrane trafficking. We further demonstrate a role for three multipass membrane proteins, SFT2D2, ZDHHC5, and GRINA, in endosome-to-Golgi retrieval.

## Highlights

• Endosome-to-Golgi retrieval is critical for diverse physiological processes
• We carried out a genome-wide siRNA screen for regulators of this pathway
• We report 88 genes whose knockdown inhibits endosome-to-Golgi retrieval
• We verify a role for three multipass membrane-spanning proteins in this pathway

## Graphical Abstract

**Figure:**
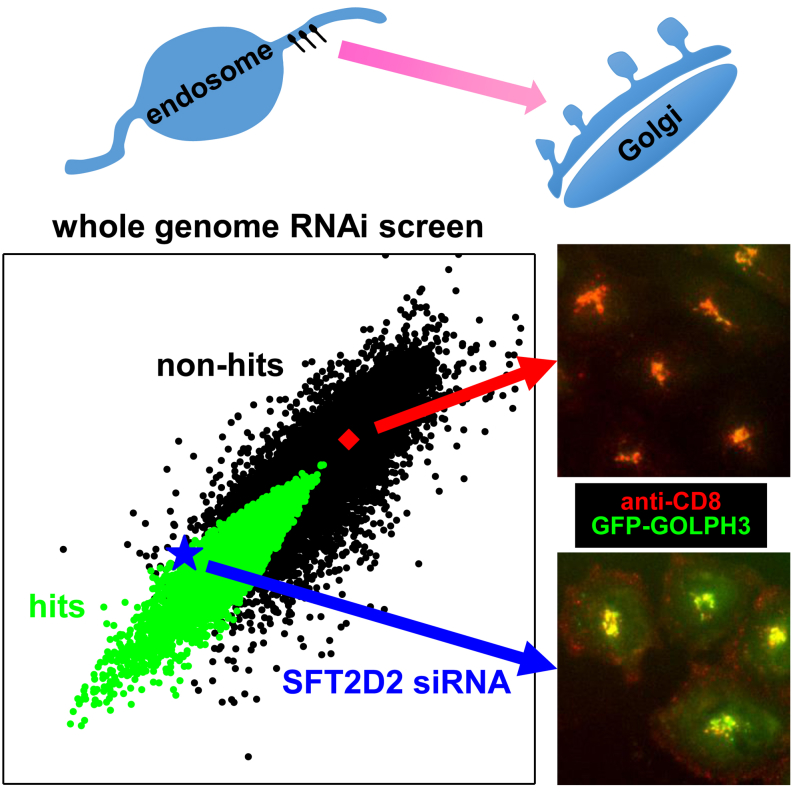

## Teaser

Breusegem et al. perform an imaging-based genome-wide siRNA screen for proteins required for endosome-to-Golgi retrieval of the cation-independent mannose-6-phosphate receptor. Their hits include several multipass membrane proteins, a class of proteins often overlooked in intracellular trafficking. Among the hits, they have characterized SFT2D2, ZDHHC5, and GRINA in endosome-to-Golgi retrieval.

## Introduction

The endosome-to-Golgi retrieval pathway is conserved across all eukaryotes, sorting a diverse set of cargo proteins that operate in lysosome biogenesis, iron homeostasis, polarity generation, and other essential cellular functions. The pathway is also at the epicenter of many pathogenic events including Alzheimer’s disease (AD), Parkinson’s disease (PD), and bacterial and viral infections. This universally conserved trafficking route functions to maintain a diverse array of membrane proteins at the Golgi. Possibly the best characterized cargo proteins for the endosome-to-Golgi pathway are the lysosomal or vacuolar hydrolase sorting receptors that mediate the transport of acid hydrolases required for lysosomal and vacuole-mediated degradation.

An essential regulator of this pathway is the retromer complex, which was first described in budding yeast (Seaman et al., 1998) and is conserved across all eukaryotes (Arighi et al., 2004; Carlton et al., 2004; Koumandou et al., 2011; Seaman, 2004). Its prototypical cargo includes the hydrolase receptors, particularly the cation-independent mannose-6-phosphate receptor (CIMPR), and it is also required for localization of the TGN marker protein TGN46, the Wnt transporter Wntless (Belenkaya et al., 2008; Yang et al., 2008), and SorL1, a member of the Vps10-domain-containing family (Fjorback et al., 2012; Nielsen et al., 2007). This interaction may therefore be relevant in AD, because SorL1 interacts with amyloid precursor protein (APP) to regulate its processing via the endosome-to-Golgi pathway, and loss of SorL1 or loss of retromer function can increase amyloidogenic processing of APP to the AD-causing Aβ form (reviewed in Fjorback and Andersen, 2012; Small, 2008; Willnow and Andersen, 2013). Some pathogens have also evolved to exploit retromer and/or endosome-to-Golgi retrieval to their own ends. For example, the human papilloma virus (HPV), following entry into the cell, interacts with retromer and is directed into an endosome-to-Golgi pathway that is believed to contribute to viral propagation within the host cell (Lipovsky et al., 2013). Furthermore, Shiga toxin produced by Shigella bacteria also utilizes retromer-mediated endosome-to-Golgi retrieval after uptake (Popoff et al., 2007). For Shiga toxin, retromer-mediated endosome-to-Golgi retrieval facilitates access to first the Golgi and then the endoplasmic reticulum where the toxin can exert its cytotoxic effects. Thus, understanding how retromer-mediated trafficking is controlled has broad implications in development and disease.

The retromer complex comprises two distinct functional units: the cargo-selective complex (CSC), which is a trimer of the Vps35, Vps29, and Vps26 proteins and a membrane-bending sorting nexin (Snx) dimer that can tubulate membranes to generate a transport intermediate (reviewed in Bonifacino and Hurley, 2008; Seaman, 2005, 2012). Although it is essential for efficient endosome-to-Golgi retrieval, retromer does not operate in isolation. The Snx dimer component of retromer (comprising SNX1 or SNX2 with SNX5 or SNX6) that mediates tubule formation also links to the microtubule cytoskeleton through interactions with p150 glued (Hong et al., 2009; Wassmer et al., 2009). These tubules are stabilized by EH-domain-containing proteins EHD1 and EHD3 (Gokool et al., 2007; McKenzie et al., 2012; Naslavsky et al., 2009). Retromer also associates with the Arp2/3-activating WASH complex that generates branched actin patches on endosomes (Harbour et al., 2010; Derivery et al., 2009; Gomez and Billadeau, 2009; and reviewed in Seaman et al., 2013). Finally, its activity is regulated by the small GTPase Rab7a, which mediates membrane recruitment of the retromer CSC (Rojas et al., 2008; Seaman et al., 2009).

Aside from retromer, only a few other factors have been linked to this trafficking pathway (including the SNARE proteins syntaxins 5, 6, 10, and 16 that mediate membrane fusion events; Ganley et al., 2008; Mallard et al., 2002; Tai et al., 2004). We therefore hypothesized that there will be many other uncharacterized components of the endosome-to-Golgi retrieval pathway, including proteins that act during retromer-mediated sorting, or independently of retromer. To address this, we have undertaken a genome-wide small interfering RNA (siRNA) screen for genes that affect endosome-to-Golgi trafficking. We have identified ∼90 genes that, when silenced, result in reduced endosome-to-Golgi retrieval. These include kinases, phosphatases, cytoskeleton-associated proteins, as well as several factors that have been linked to PD. Notably, several of the genes encode multipass membrane-spanning proteins—a class of proteins often overlooked with respect to a role in membrane trafficking. We have characterized three of these multipass membrane proteins, SFT2D2, ZDHHC5, and GRINA, to further verify their function in endosome-to-Golgi retrieval.

## Results

### Anti-CD8 Antibody Uptake Assay for siRNA Screening

CIMPR is a prototypical cargo for retromer-mediated retrieval in the endosome-to-Golgi pathway. It binds hydrolases at the *trans*-Golgi network (TGN), is packaged into clathrin-coated vesicles for delivery to endosomes, and is then recycled back to the Golgi by retromer for further rounds of hydrolase sorting. We and others have used HeLa cells stably expressing a CD8-CIMPR chimera in combination with immunofluorescence to assay the trafficking routes and protein interaction partners of the CIMPR cargo protein (Carlton et al., 2004; Harasaki et al., 2005; Seaman, 2004; Wassmer et al., 2007). In particular, we have shown that the efficient retrieval of the CD8-CIMPR reporter from endosomes to the Golgi requires retromer (Seaman, 2004). In addition, we have used a cell line stably expressing both CD8-CIMPR and GFP-tagged GOLPH3, a peripheral Golgi protein (Wu et al., 2000), to evaluate the role of retromer-interacting proteins (e.g., members of the WASH complex, TBC1D5, SNX3, and Rab7a) in endosome-to-Golgi retrieval of the CD8-CIMPR reporter (Harbour et al., 2010).

**Figure 1:**
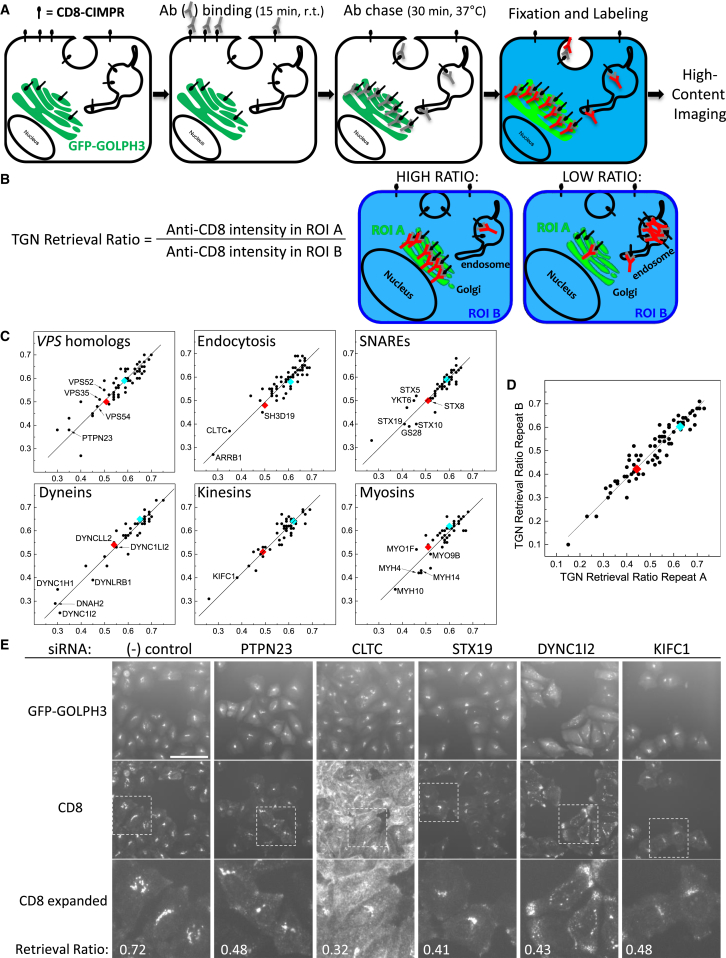
Pilot Screen for Known Regulators of Endosome-to-Golgi Retrieval Using Anti-CD8 Uptake Assay (A) Schematic of the anti-CD8 antibody retrieval assay as adapted for high-throughput screening. HeLa cells stably expressing a CD8-CIMPR reporter and the Golgi protein GFP-GOLPH3 were used. Anti-CD8 antibody (Ab) was bound at room temperature for 15 min and chased at 37°C for 30 min. Labeling and imaging details are in Supplemental Experimental Procedures. (B) Definition of the TGN retrieval ratio used in our studies and depiction of subcellular antibody localizations that give rise to high or low TGN retrieval ratios. (C) Scatterplots of the TGN retrieval ratios measured in replicate screens of 60 *VPS* gene homologs, 60 endocytosis genes, 43 SNARE protein genes, 41 dynein genes, 49 kinesin genes, and 43 myosin genes. Negative control measurements are in blue; SNX1 siRNA positive control measurements are in red. A linear fit to the data points is also shown. Marked data points indicate known endosome-to-Golgi retrieval pathway genes or genes that were selected for further validation. (D) Replicate TGN retrieval ratios for the validation screen of the pilot study, in which 80 individual ON TARGETplus siRNA sequences (four per gene) were assayed. Controls and linear fit are as in (C). (E) Representative images of the pilot validation screen for the negative control (no siRNA) and for five sets of siRNA-treated cells with reduced TGN retrieval ratio. Different phenotypes that give rise to reduced TGN retrieval ratios are discussed in the text. Scale bar, 100 μm (top two rows) and 38 μm (bottom row).

We therefore utilized the CD8-CIMPR reporter in a genome-wide siRNA screen to uncover proteins that act in endosome-to-Golgi retrieval. Our protocol employed siRNA transfection of cells stably expressing CD8-CIMPR and GFP-GOLPH3 in 96-well plates, followed by a three-step semiautomated anti-CD8 antibody uptake assay depicted in Figure 1A and detailed in Breusegem and Seaman (2014). The assay relies on a proportion of CD8-CIMPR being present at the cell surface at any given time. Thus, to assay endosome-to-Golgi retrieval the uptake of the CD8-CIMPR reporter by endocytosis is a necessary first step.

Images were acquired on an automated microscope, and, for every selected cell imaged, the retrieval of the anti-CD8 antibody to the TGN was quantified as a TGN retrieval ratio (Figure 1B). For each siRNA, TGN retrieval ratios were averaged over all measured cells and compared to average TGN retrieval ratios of negative and positive control wells.

### Pilot Screen with Known Trafficking Genes

We first assessed our experimental workflow by assaying six mini-siRNA libraries, each containing between 41 and 60 ON TARGETplus siRNA pools that target human genes coding for known regulators of intracellular trafficking or cytoskeletal dynamics. In total, 310 individual genes were assayed in duplicate (data in Table S1), and this duplicate screen was repeated to assess biological reproducibility. Technical replicates showed high correlation coefficients for the TGN retrieval ratio (Figure 1C). Knockdown (KD) of most of the assayed genes yielded a TGN retrieval ratio that was similar to the value measured for the negative control wells (cyan data point, between 0.6 and 0.7), whereas KD of a few genes in each mini-library yielded a TGN retrieval ratio equal to or less than the one measured for the positive control cells (SNX1 KD, red data point, between 0.45 and 0.55). Biological replicates reproducibly generated a set of genes for which KD by siRNA led to a measured TGN retrieval ratio that was smaller than the one measured for SNX1 KD. This “hit” list included proteins with a well-documented role in endosome-to-Golgi retrieval and/or endocytosis (e.g., the retromer components VPS35 and SNX1, the GARP complex proteins VPS52 and VPS54, clathrin heavy chain, Golgi SNARE proteins STX5, STX10, GS15, and GS28). It also identified proteins that have not so far been linked to endosome-to-Golgi retrieval, e.g., PTPN23, ARRB1, and STX19.

To validate the role of these proteins in endosome-to-Golgi retrieval, the anti-CD8 uptake assay was repeated using the individual siRNA sequences of the ON TARGETplus pool in four separate siRNA transfections for 20 selected genes (Figure 1D). Ten out of the 20 genes were validated with two or more single siRNA oligos (Table S1). Using automated microscopy, both control cells and cells transfected with individual siRNA sequences (both of which express CD8-CIMPR and GFP-GOLPH3) were imaged after anti-CD8 antibody uptake. Example images in Figure 1E show that changes in anti-CD8 localization correlate with a decreased TGN retrieval ratio. For example, clathrin heavy chain (CLTC) KD results in a large fraction of anti-CD8 antibody binding to plasma membrane localized CD8-CIMPR, indicative of a defect in the endocytic uptake of the CD8-CIMPR reporter. In contrast, KD of DYNC1I2 causes an accumulation of anti-CD8 in cytoplasmic vesicles located at the cell periphery. Other examples in Figure 1E display intermediate phenotypes with reduced levels of anti-CD8 at the TGN and increased amounts of anti-CD8 in cytoplasmic vesicles.

Thus, the pilot screens provide a strong proof-of-principle that this strategy can be used to identify bona fide regulators of endosome-to-Golgi retrieval and candidate genes for further testing.

### Genome-wide Primary Screen

**Figure 2:**
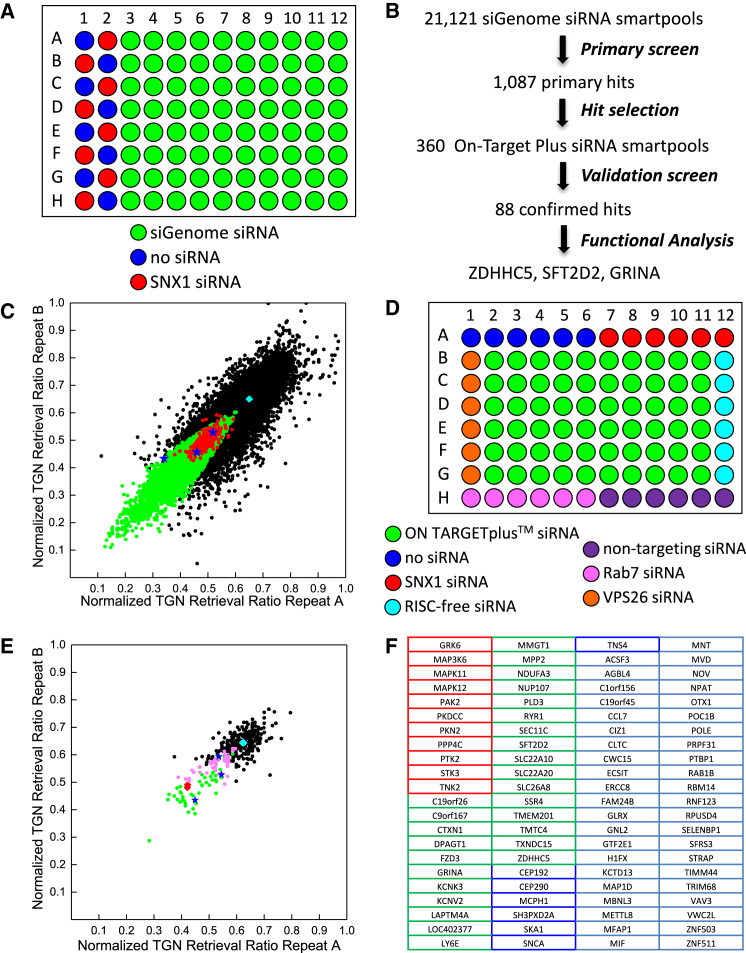
A Whole-Genome siRNA Screen to Identify Regulators of Endosome-to-Golgi Retrieval (A) Plate layout for genome-wide loss-of-function screen. (B) Whole-genome screen hit selection. (C) Scatterplot of the normalized TGN retrieval ratios for the two replicates of the whole-genome screen. Each data point represents a single siRNA pool. Only valid measurements (18,465 siRNA pools) are included. Negative (cyan square) and positive (SNX1 siRNA, red data points) controls are shown. Green data points indicate siRNA pools with TGN retrieval ratio SSMD ≤ (−3) (very strong hits). Blue star data points indicate the three hits characterized further in Figures 3, 4, 5, and 6. (D) Plate layout for the ON TARGETplus validation screen. (E) Replicate normalized TGN retrieval ratios for the ON TARGETplus smartpool validation screen (360 pools). The colors used are as in (C), with additional magenta data points indicating siRNA pools with TGN retrieval ratio SSMD between (−2) and (−3) (strong hits). (F) Table listing the 88 genes that were very strong or strong hits in the validation screen. Red-bordered cells indicate kinase and phosphatase genes, green-bordered cells contain genes encoding membrane proteins, and blue-bordered cells contain genes encoding cytoskeletal proteins. More information about these genes is provided in Table S4.

We next carried out a genome-wide screen for effectors of endosome-to-Golgi retrieval of CIMPR using this same strategy with a human siRNA library. The genome-wide siRNA library contained 21,121 siGenome siRNA pools arrayed onto 267 library plates (Figure 2A). TGN retrieval ratios were normalized and strictly standardized mean difference (SSMD) values were calculated, both for the normalized TGN retrieval ratio and for the cellular anti-CD8 intensity, to allow hit selection based on a statistical analysis (see Experimental Procedures). Figure 2B summarizes the genome screen workflow, whereas Figure 2C shows a plot of the normalized TGN retrieval ratios measured in the two replicates of the genome-wide screen. Only valid data points are included, i.e., siRNAs that cause cell toxicity or severe cell division defects are not included. As in the pilot screen, data points are scattered around the diagonal indicating good reproducibility (correlation coefficient 0.88). Green data points represent 4,556 siRNAs with TGN retrieval ratio SSMD values smaller than (−3), indicating very strong siRNA effects. Positive control cells (SNX1 siRNA) also had a TGN retrieval ratio SSMD ≤ (−3) on the majority of the plates (red data points in Figure 2C). Further hit selection criteria included HeLa cell gene expression data and exclusion of hits that had been removed from the NCBI database (see Experimental Procedures for full details). This resulted in 1,087 hits from the genome-wide screen (∼5% of the siRNAs screened) (Table S2).

### Validation Screen

We selected 360 genes for further validation in a follow-up siRNA screen. For this, we arrayed ON TARGETplus siRNA pools in the 60 central wells of six 96-well plates (Figure 2D). The ON TARGETplus siRNA pools comprise, in many cases, completely distinct sequences from the ones in the siGenome siRNA pools and therefore are an effective means of validating the phenotype identified in the primary screen.

Similarly to the primary screen, we first assayed endosome-to-Golgi retrieval in HeLa cells stably expressing CD8-CIMPR and GFP-GOLPH3. The results are listed in Table S3, whereas Figure 2E shows the TGN retrieval ratios measured for the 360 genes in duplicate. Statistical analysis of the normalized TGN retrieval ratios yielded 44 very strong hits [12% of assayed genes with SSMD ≤ (−3), including clathrin, which is required for the uptake of the CD8-CIMPR reporter]. Because the ON TARGETplus siRNA pools are designed to higher specificity standards than the siGenome siRNA pools (Jackson et al., 2006), we also included 44 strong hits [−3 < SSMD ≤ (−2)] in our table of hits (Figure 2F). Interestingly, PLD3, a gene recently linked to late-onset AD (Cruchaga et al., 2014), was among the confirmed hits, whereas pathway analysis indicated a significant enrichment in genes linked to PD (SNCA, MAPK11, MAPK12). Further hits included genes with diverse predicted functions (kinases/phosphatases, red border in Figure 2F) and cellular locations (e.g., cytoskeleton, blue border in Figure 2F) as well as several membrane proteins (green border in Figure 2F). Available functional information, cellular location, and aliases of the 88 hit proteins are listed in Table S4.

### Detailed Hit Characterization: SFT2D2, ZDHHC5, and GRINA

Closer inspection of the hits (Figure 2F) indicated a large number of multipass membrane-spanning proteins, including KCNK3, ZDHHC5, SLC22A10, SFT2D2, and GRINA. Many of these are high-confidence hits, i.e., the primary screen siGenome and validation screen ON TARGETplus siRNA pools for these genes did not share any sequences (Table S4). Current protein structure prediction tools suggest that ∼26% of human genes encode membrane-spanning proteins, with almost half of these being multipass membrane proteins (Fagerberg et al., 2010; Kanapin et al., 2003). Yet, few multipass membrane-spanning proteins have been shown to affect membrane trafficking, and, to our knowledge, they have not been linked to endosome-to-Golgi retrieval in mammals. Therefore, we selected three multipass membrane-spanning high-confidence hits for further study: SFT2D2, ZDHHC5, and GRINA.

#### SFT2D2

**Figure 3:**
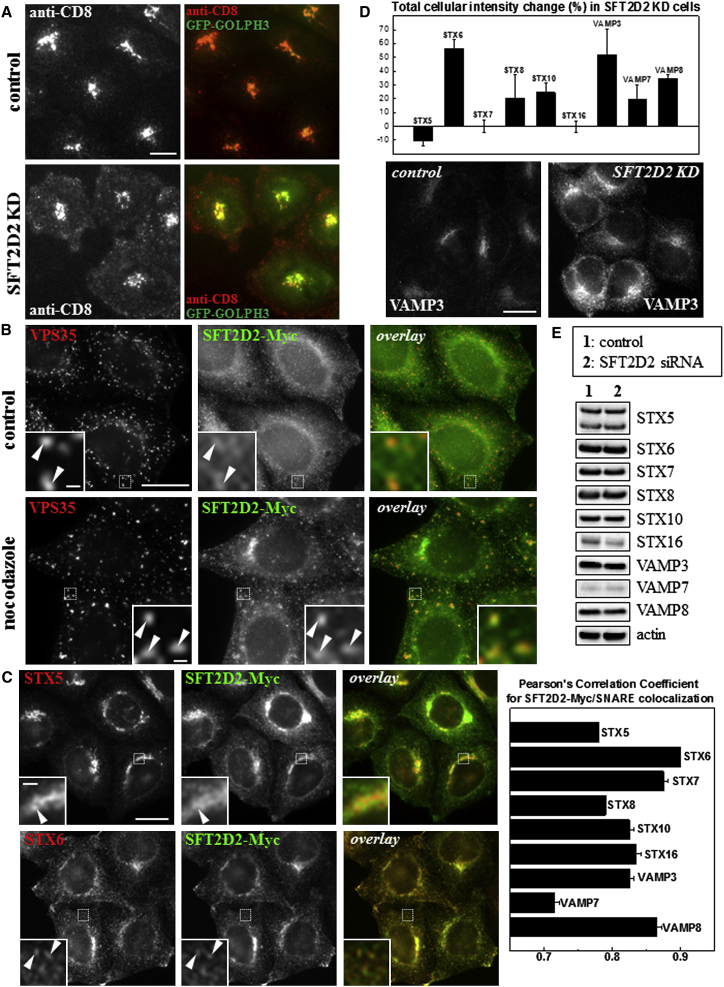
Characterization of SFT2D2 Function in Endosome-to-Golgi Retrieval (A) Primary screen anti-CD8 antibody-uptake images for control cells (top) and SFT2D2 KD cells (bottom). (B) Cells stably expressing SFT2D2-Myc show localization of SFT2D2 to perinuclear membranes and endosomes, including VPS35-positive endosomes (arrowheads in inset). Treatment with nocodazole disperses the endosomes and even more clearly shows colocalization of SFT2D2-Myc and VPS35 (arrowheads in inset). (C) Cells stably expressing SFT2D2-Myc were costained with antibodies against various post-Golgi SNARE proteins. Colocalization was quantified by Pearson’s correlation coefficient (right-hand graph) and indicates very extensive colocalization with STX6, STX7, and VAMP8. The image panels illustrate colocalization of SFT2D2-Myc and STX5 at the Golgi (top, arrowhead) but much more extensive colocalization of SFT2D2-Myc and STX6 (bottom, arrowheads). In (B) and (C), the white dashed box delineates the area magnified in the insets. (D) SNARE protein staining was compared for control and SFT2D2 KD HeLa cells and quantified. The graph shows the change in cellular intensity measured for each post-Golgi SNARE investigated. Images illustrate the increased cellular intensity of VAMP3 in SFT2D2 KD cells. (E) Control and SFT2D2 KD HeLa cell lysates were separated by LDS-PAGE and blotted for the indicated SNARE proteins or actin. Scale bars in (A)–(D), 20 μm, except insets in (B), 1 μm, and in (C), 2 μm. Quantitation in (C) and (D) was done using automated microscopy (see Experimental Procedures); error bars indicate SD of two separate multicell experiments. Average Pearson’s correlation coefficients measured were often identical in repeated experiments.

SFT2D2 is partially homologous to the yeast Sft2p protein, a genetic interactor of Sed5p (the yeast syntaxin 5 protein) and affects post-Golgi trafficking (Conchon et al., 1999). However, the mammalian homologs (SFT2D1, SFT2D2, and SFT2D3) are uncharacterized. Images of anti-CD8 localization obtained in the primary screen show an accumulation of antibody in peripheral puncta in SFT2D2-silenced cells compared to control HeLa cells (Figure 3A) as well as reduced levels of antibody at the TGN.

To determine where in the endosome-to-Golgi pathway SFT2D2 could be acting, we established a cell line stably expressing Myc-tagged SFT2D2. We find that SFT2D2 localizes to both perinuclear membranes and structures positive for the retromer CSC protein, VPS35. The colocalization of SFT2D2 with VPS35 is especially apparent after treatment with nocodazole to depolymerize microtubules (Figure 3B). The reported genetic interaction between yeast Sft2p and Sed5 (Conchon et al., 1999) prompted us to evaluate the localization of SFT2D2 with respect to a number of SNARE proteins that function in post-Golgi trafficking. We find that SFT2D2 partially colocalizes with syntaxin 5 but exhibits almost complete colocalization with syntaxin 6 (indicated by arrowheads in the immunofluorescence images shown in Figure 3C). Quantitation of the colocalization of SFT2D2 with a number of post-Golgi SNARE proteins is shown graphically in Figure 3C (right panel) and confirms that SFT2D2 resides in a compartment strongly positive for syntaxin 6.

Extending the immunofluorescence-based investigation of SNARE protein distribution, when SFT2D2 expression is silenced by RNAi, we observed a marked change in the fluorescence intensity of several SNARE proteins, with syntaxin 6 and VAMP3 exhibiting the strongest change (Figure 3D). An example of the altered fluorescence intensity for VAMP3 is shown along with the graph in Figure 3D. Surprisingly, however, changes in the fluorescence intensity are not the result of changes in overall levels of the respective SNARE proteins (Figure 3E).

#### ZDHHC5

**Figure 4:**
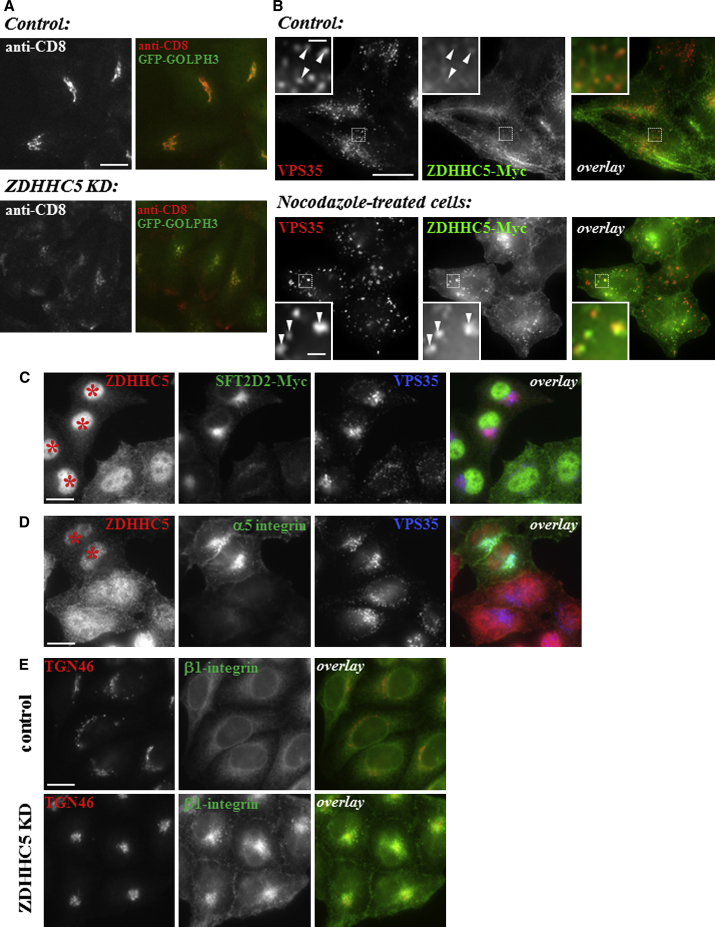
Localization and Characterization of ZDHHC5 in HeLa Cells (A) Primary screen anti-CD8 antibody-uptake images for control cells (top) and ZDHHC5 KD cells (bottom). (B) Cells stably expressing ZDHHC5-Myc show localization of ZDHHC5 to the plasma membrane and to intracellular tubules and vesicles. Some colocalization between ZDHHC5 and retromer VPS35 is observed (arrowheads in inset). Following nocodazole treatment endosomes are dispersed and some are labeled with ZDHHC5 and VPS35 (arrowheads in inset). (C) Control and ZDHHC5 siRNA-treated SFT2D2-Myc cells were mixed and stained for ZDHHC5, Myc, and VPS35. KD cells are marked by an asterisk. (D) Control and ZDHHC5 siRNA-treated HeLa cells were mixed and stained for ZDHHC5, α5-integrin, and VPS35. KD cells are indicated with an asterisk. (E) Control (top) and ZDHHC5 KD (bottom) HeLa cells were fixed and stained for TGN46 and β1-integrin. Scale bars in (A)–(E), 20 μm, except inset in (B), 2 μm.

ZDHHC5 is a palmitoyl-acyl transferase enzyme that has been shown to palmitoylate a number of substrates, including the somatostatin receptor 5 (SSR5), flotillin-2, and GRIP1b (Kokkola et al., 2011; Li et al., 2012; Thomas et al., 2012). Its KD resulted in decreased amounts of anti-CD8 localization at the TGN and an increase in anti-CD8 positive puncta close to the plasma membrane following our antibody-uptake protocol (Figure 4A).

The ZDHHC5 protein, unlike most palmitoyl transferases, has an extensive C-terminal domain with four tyrosine-containing YxxΦ motifs that indicate ZDHHC5 undergoes clathrin-mediated sorting. Indeed, in cells stably expressing a Myc-tagged ZDHHC5 protein, we find that ZDHHC5 localizes extensively to the plasma membrane but also to intracellular tubular and vesicular structures (Figure 4B). As detailed in Figure S1, we further characterized the tubular structures on which ZDHHC5 is localized and found them to be Rab8a- or Rab11-positive recycling tubules. Figure 4B illustrates the partial colocalization of ZDHHC5 with the retromer CSC protein VPS35, which becomes more prominent after nocodazole treatment.

A reported substrate for ZDHHC5 is the SFT2D2 protein. Therefore, in cells expressing SFT2D2-Myc, ZDHHC5 expression was silenced using RNAi. Treated and control cells were mixed and seeded onto coverslips and then labeled with antibodies against ZDHHC5, Myc, and VPS35. In cells where ZDHHC5 expression was silenced (marked with ^∗^), the SFT2D2 staining appeared brighter and more concentrated in the perinuclear region (Figure 4C).

The retromer and the WASH complexes can mediate endosome-to-plasma membrane recycling in addition to endosome-to-Golgi retrieval (Derivery et al., 2009; Gomez and Billadeau, 2009; Steinberg et al., 2013; Temkin et al., 2011). Therefore, we examined the effect of ZDHHC5 KD on the localization of α5β1-integrin, a cargo protein of retromer in the endosome-to-plasma membrane pathway (Duleh and Welch, 2012; Zech et al., 2011). Both plasma membrane localized and intracellular stores of α5-integrin (Figure 4D), as well as β1-integrin (Figure 4E), are markedly increased in ZDHHC5-silenced cells (marked by an asterisk in Figure 4D) compared to control cells.

In separate experiments, we assessed and quantified the localization and expression of several post-Golgi SNARE proteins upon ZDHHC5 KD (Figure S2). Although immunofluorescence indicated increased staining of several (STX6, STX7, STX8) but not all (e.g., STX16) SNARE proteins, total cellular SNARE protein levels as assessed by western blotting were unchanged upon ZDHHC5 KD (Figure S2).

#### GRINA

**Figure 5:**
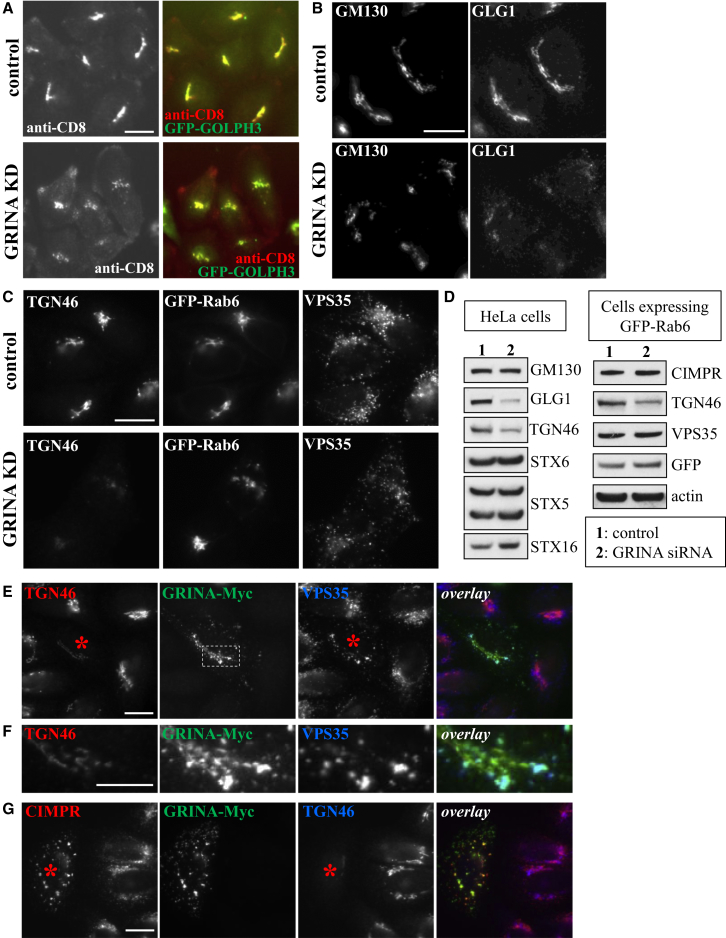
GRINA Knockdown or Overexpression Inhibits Endosome-to-Golgi Retrieval (A) Primary screen anti-CD8 antibody uptake images for control cells (top) and GRINA KD cells (bottom). (B) Control (top) and GRINA KD (bottom) HeLa cells were stained for Golgi marker GM130 and Golgi glycoprotein-1 (GLG1). (C) Cells stably expressing GFP-Rab6 were treated with GRINA siRNA (bottom) and compared to control cells (top) upon staining for TGN46 and VPS35. (D) Quantitation by western blotting of lysates from control and GRINA-silenced HeLa cells and GFP-Rab6 cells. (E–G) HeLa cells were transiently transfected with GRINA-Myc for 24 hr before fixing and staining. (E and F) GRINA-Myc colocalizes with both TGN46 and VPS35. In this example, GRINA-Myc expression (in the cell marked by an asterisk in E) reduced the cell’s TGN46 expression compared to surrounding untransfected cells and caused enlargement of VPS35-positive endosomes. The area in (E) magnified in (F) is indicated by a dashed line. (G) GRINA-Myc expression also perturbs CIMPR localization. In this example, GRINA-Myc transfection (in the cell marked by an asterisk) caused CIMPR to localize to round vesicular structures positive for GRINA-Myc, some of which appeared larger than regular endosomes, whereas TGN46 staining was almost absent in the transfected cell. Scale bars in (A)–(C), (E), and (G), 20 μm, and in (F), 10 μm.

GRINA (*g*lutamate *r*eceptor, *i*onotropic, *N*-methyl D-aspartate-*a*ssociated protein 1), also known as LFG1 or TMBIM3, is a 42 kDa protein with a conserved BAX inhibitor-1 motif and a reported role in protecting cells from ER stress-induced apoptosis (Rojas-Rivera et al., 2012). In addition, a C-terminal fragment of the protein protects HeLa cells from Shiga-toxin induced cytotoxicity (Yamaji et al., 2010). Images from the primary screen indicated that KD of GRINA in HeLa cells expressing CD8-CIMPR and GFP-GOLPH3 leads to a reduction in anti-CD8 antibody that reaches the TGN after a 30 min chase period (Figure 5A). In addition, we noted that our chosen TGN marker, GOLPH3, appeared slightly fragmented in the GRINA KD cells. Further characterization of cells in which GRINA expression was suppressed using siRNA showed reduced levels of TGN46 at the TGN (Figure 5C) and in total cell lysates (Figure 5D), whereas GFP-Rab6 levels and appearance were unaltered (Figures 5C and 5D).

GRINA KD severely altered expression of GLG1, a type-I Golgi membrane protein that traffics from endosomes to the Golgi (Ahn et al., 2005) (Figure 5B and leftmost western blot in Figure 5D), but had no effect on *cis*-Golgi localized GM130 levels (Figure 5D, rightmost western blot). The effect of the loss of GRINA on other Golgi localized proteins (e.g., α-mannosidase II and STX5) or endo/lysosomal proteins (e.g., EEA1 and LAMP1) is shown in Figure S3. In some instances, fragmentation of the *cis*-Golgi was observed in cells in which GRINA was silenced (see Figure 5B), but no changes were observed in the distribution or intensity of the core retromer protein VPS35 (Figures 5C and S3A).

Transient transfection of HeLa cells with Myc-tagged GRINA had pronounced effects on both TGN and endosome morphology. In particular, transfected cells showed a shrunken TGN with reduced levels of TGN46, whereas endosomes appeared enlarged (Figures 5E and 5F). Myc-tagged GRINA colocalized with the remaining TGN46 and also extensively with VPS35-positive (Figure 5F) and SNX1-positive (Figure S3D) endosomes, including endosomes that appeared enlarged. GRINA overexpression severely disrupted CIMPR staining, with CIMPR found in GRINA-positive vesicular structures, some enlarged, scattered throughout the cell (Figure 5G).

### SFT2D2, ZDHHC5, and GRINA Loss Does Not Disrupt Retromer Complex but Leads to Altered Levels of Endosome-to-Golgi Cargo Proteins

To address how SFT2D2, ZDHHC5, or GRINA might affect endosome-to-Golgi retrieval and CIMR trafficking, we performed a biochemical analysis of how their loss affects the integrity of the retromer complex or its interaction with the WASH complex. Native coimmunoprecipitation experiments determined that KD of SFT2D2, ZDHHC5, or GRINA did not affect the protein interactions between the cargo-selective retromer components VPS26, VPS29, and VPS35, nor the interaction between these core retromer components and WASH complex members (strumpellin, FAM21) or TBC1D5, suggesting that SFT2D2, ZDHHC5, and GRINA do not act directly on the cargo-selective retromer complex or the WASH complex (Figure S4A).

**Figure 6:**
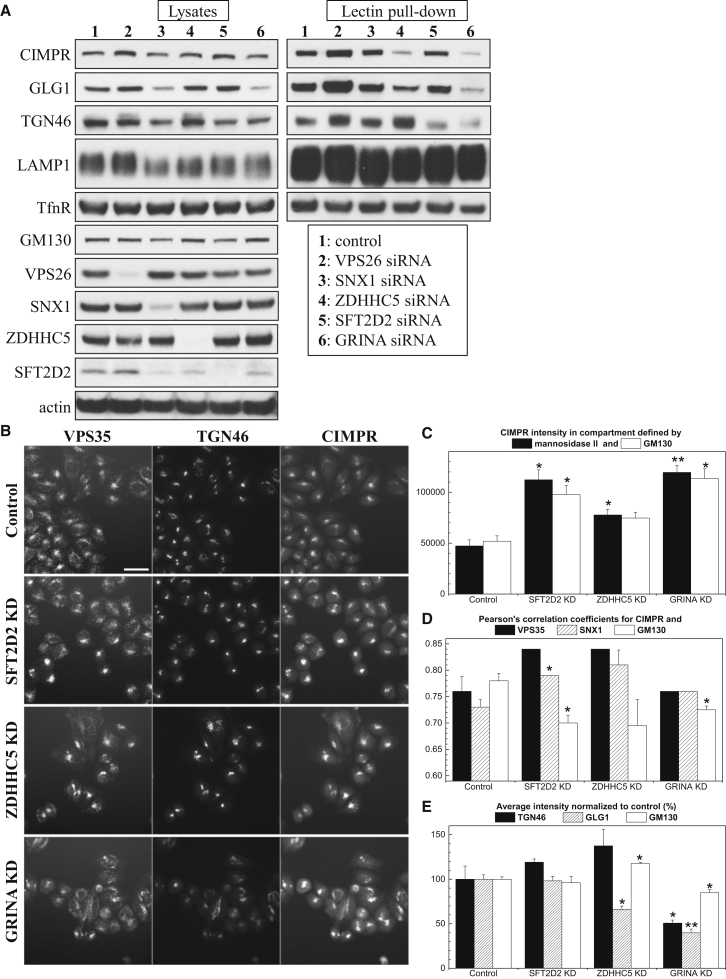
ZDHHC5, SFT2D2, or GRINA Depletion Affect Levels of Endosome-to-Golgi Cargo Proteins (A) Control HeLa cells or cells transfected with the indicated siRNAs were treated for 3 hr with cycloheximide, lysed, and incubated with agarose-bound wheat germ agglutinin to capture glycosylated membrane proteins. Total cell lysates (left) and lectin pull-down samples (right) were assayed by western blotting. The experiment was repeated three times, and representative data are shown. (B–E) Quantitative analysis of SFT2D2, ZDHHC5, and GRINA KD cells immunofluorescence using automated microscopy (see Experimental Procedures). (B) Representative images showing VPS35, TGN46, and CIMPR staining. Scale bar, 50 μm. (C) Quantitative analysis of CIMPR intensity at the Golgi indicates a significant increase in SFT2D2, ZDHHC5, or GRINA KD cells. (D) SFT2D2, ZDHHC5, and GRINA KD increase the Pearson’s correlation coefficient for colocalization between CIMPR and retromer proteins VPS35 or SNX1 while decreasing the correlation between CIMPR and Golgi matrix protein GM130. In some cases, identical correlation coefficients were measured in the replicate experiments. (E) Quantitation of the TGN46 and GLG1 intensity in the three types of KD cells. In (C)–(E), error bars indicate the SD of the replicate measurements. ^∗^p < 0.05; ^∗∗^p < 0.01.

We next assessed whether silencing of SFT2D2, ZDHHC5, or GRINA affected levels of retromer cargo proteins. In addition to blotting for various membrane proteins in crude lysates, immobilized wheat germ agglutinin (WGA), a lectin that binds to galactose and sialic acid moieties, was used to enrich for Golgi and post-Golgi localized glycosylated membrane proteins from lysates of control or KD cells where the retromer components VPS26 or SNX1, or SFT2D2, ZDHHC5, or GRINA, were silenced (Figure 6A). Silencing of ZDHHC5 or SFT2D2, respectively, resulted in reduced levels of CIMPR or TGN46 bound to the lectin, whereas silencing GRINA reduced lectin-bound levels of GLG1, CIMPR, and TGN46 but also decreased their abundance in the lysates from cycloheximide-treated cells (Figure 6A; Figure S4B). Importantly, levels of the transferrin receptor (TfnR) that cycles from endosomes to the cell surface were not affected in any of the KDs. Interestingly, levels of SFT2D2 were found to be strongly affected by the KD of SNX1, suggesting that its steady-state localization may be regulated by retromer.

Using automated microscopy, we next examined the fluorescence intensity of two endogenous proteins known to traffic from endosomes to the TGN in a retromer-dependent manner: the CIMPR and TGN46 (Figure 6B). We find that RNAi-mediated KD of SFT2D2, ZDHHC5, or GRINA all lead to an increase in the CIMPR fluorescence intensity although GRINA KD markedly reduces TGN46 staining. Quantitative analysis shows increased CIMPR fluorescence intensity in Golgi structures marked by mannosidase II or GM130 (Figure 6C) and also, for KD of SFT2D2 and ZDHHC5, increased correlation coefficients for CIMPR and VPS35 (Figure 6D). VPS35 endosomes are also significantly brighter in the three types of KD cells compared to control cells (Figure S4C). The RNAi-mediated silencing of GRINA appears to result in some loss of Golgi integrity and results in a general reduction in fluorescence intensity for both TGN46 and GLG1 (Figure 6E).

Collectively, these experiments indicate that SFT2D2 and ZDHHC5 are important for recycling of specific cargo proteins to the Golgi, whereas loss of GRINA may have more wide-ranging effects on TGN structure and stability of a number of membrane proteins.

## Discussion

Endosome-to-Golgi retrieval is an essential pathway in several key physiological processes and is exploited by bacterial and viral pathogens, underscoring the importance of expanding our understanding of the pathway. Here, we have conducted a genome-wide RNAi screen using an antibody-uptake assay and a model cargo reporter to identify ∼90 proteins that, when silenced, impede the endosome-to-Golgi retrieval. This included a number of kinases, phosphatases, and several multipass membrane spanning proteins that were uncharacterized with respect to endosome-to-Golgi retrieval.

We successfully adapted our antibody-uptake assay for this high-throughput screen. A pilot screen of siRNA mini-libraries that targeted known membrane trafficking genes demonstrated the feasibility of using the CD8 antibody-uptake assay in 96-well plates, identifying known endosome-to-Golgi components such as retromer VPS35 and the GARP complex proteins VPS52 and VPS54 (Pérez-Victoria et al., 2008) as hits. In addition, the pilot screen identified proteins with a role in endosome-to-Golgi retrieval that can now be further investigated, e.g., PTPN23 (also known as HD-PTP), KIFC1 (also known as HSET), and STX19.

Our genome-wide screen generated ∼1,100 hits, including known endosome-to-Golgi participants, such as VPS26, SNX6, VPS54, and endocytic proteins such as clathrin. Other known components of the endosome-to-Golgi retrieval pathway fell just below our stringent hit selection cutoff (e.g., VPS29, SNX5, Rab7a, VPS52). Our distribution of hits is similar to other reported screens, e.g., the screen that reported a role for retromer in HPV infectivity (Lipovsky et al., 2013). The presence of genes known to mediate endocytosis (e.g., clathrin heavy chain) among the hits is a predictable consequence of the use of the antibody-uptake assay. The accumulation of anti-CD8 at the cell surface observed in a clathrin KD (see Figure 1E) provides a useful benchmark against which to compare the other hits reported in our study, and, among the ∼90 endosome-to-Golgi genes we report, none produce a phenotype like the clathrin KD.

We validated our hits from the primary screen using ON TARGETplus siRNA pools. Not only are the four siRNA duplexes in these second generation siRNA pools chemically modified to avoid off-target effects, their sequences are also, for a great many genes, entirely different from the ones in our whole-genome library (siGenome siRNA). They are therefore an effective means of validating our primary screen hits.

Among the confirmed high-confidence hits, we noted several kinases, phosphatases, and proteins that have been implicated in PD. As the retromer protein, VPS35, has been revealed to be a PD gene (Vilariño-Güell et al., 2011; Zimprich et al., 2011), the identification of other PD genes among the hits is consistent with the important role that endosomal protein sorting plays in the pathology of PD.

There was also an enrichment for multipass membrane spanning proteins, a group often overlooked with respect to membrane trafficking regulation. In fact, very few multipass membrane-spanning proteins have a reported role in membrane trafficking. Examples to date include the function of Atg9 in autophagy (reviewed in Reggiori and Tooze, 2012) and that of CLN3 in anterograde post-Golgi trafficking (Metcalf et al., 2008). Thus, this screen has revealed a role for this class of proteins in endosome-to-Golgi retrieval.

We chose three multipass membrane spanning proteins for further characterization: SFT2D2, ZDHHC5, and GRINA. Although each of them, when silenced, had a strong effect on endosome-to-Golgi retrieval of CD8-CIMPR and also affected other cargoes of the endosome-to-Golgi pathway such as TGN46 and GLG1, none affected retromer integrity or compromised retromer association with proteins such as the WASH complex or TBC1D5. Additionally, no association between retromer and the SFT2D2, ZDHHC5, or GRINA proteins has been detected thus far (S.Y.B. and M.N.J.S., unpublished data).

SFT2D2 is evolutionarily conserved, and yeast Sft2p, first described as a genetic interactor of Sed5p (the fungal syntaxin 5 protein; Banfield et al., 1995), has been implicated in post-Golgi membrane traffic (Conchon et al., 1999). We report that SFT2D2 localizes to structures positive for TGN and endosomal markers. ZDHHC5 is a palmitoyl transferase reported to localize to the plasma membrane (Ohno et al., 2006). The extended cytoplasmic tail of ZDHHC5 contains several YxxΦ motifs required for clathrin-mediated sorting processes and marks out ZDHHC5 as distinct from most other palmitoyl transferases (Korycka et al., 2012). We show localization of ZDHHC5-Myc to retromer-positive endosomes and endosomal recycling tubules positive for EHD1, Rab8, and Rab11. EHD1 operates with retromer in endosome-to-Golgi retrieval (Gokool et al., 2007; Zhang et al., 2012), and both EHD1 and Rab11-mediated endosomal protein recycling have recently been shown to regulate localization and processing of APP consistent with the prominent role that endosomal protein sorting plays in AD (Buggia-Prévot et al., 2013; Udayar et al., 2013). Palmitoylation of the cytoplasmic tail of the CIMPR does facilitate its endosome-to-Golgi retrieval, but this modification is mediated by ZDHHC15, a palmitoyl transferase distinct from ZDHHC5 (McCormick et al., 2008). Interestingly, SFT2D2 has been identified as a substrate of ZDHHC5 (Li et al., 2012), and we indeed find that ZDHHC5 KD affects the localization of SFT2D2.

The KD of either SFT2D2 or ZDHHC5 resulted in changes to the fluorescence intensity of several SNARE proteins. For example, loss of SFT2D2 markedly increased the fluorescence intensity of syntaxin 6 and VAMP3. These two SNAREs have been shown to function together (Riggs et al., 2012), so it is perhaps not surprising that both are affected by SFT2D2 KD. Although the fluorescence intensity of several SNAREs was affected by SFT2D2 or ZDHHC5 KD, western blotting indicated that overall levels of the SNARE proteins were not altered. The increased fluorescence must therefore result from either altered localization of the respective SNARE, so it is more concentrated in a discrete localization, and hence the fluorescence intensity is increased, or from changes in the conformation of the respective SNARE such that the antisera used for the immunofluorescence staining has greater access to its epitope resulting in increased fluorescence intensity. Further work is required to distinguish these two possibilities.

**Figure 7:**
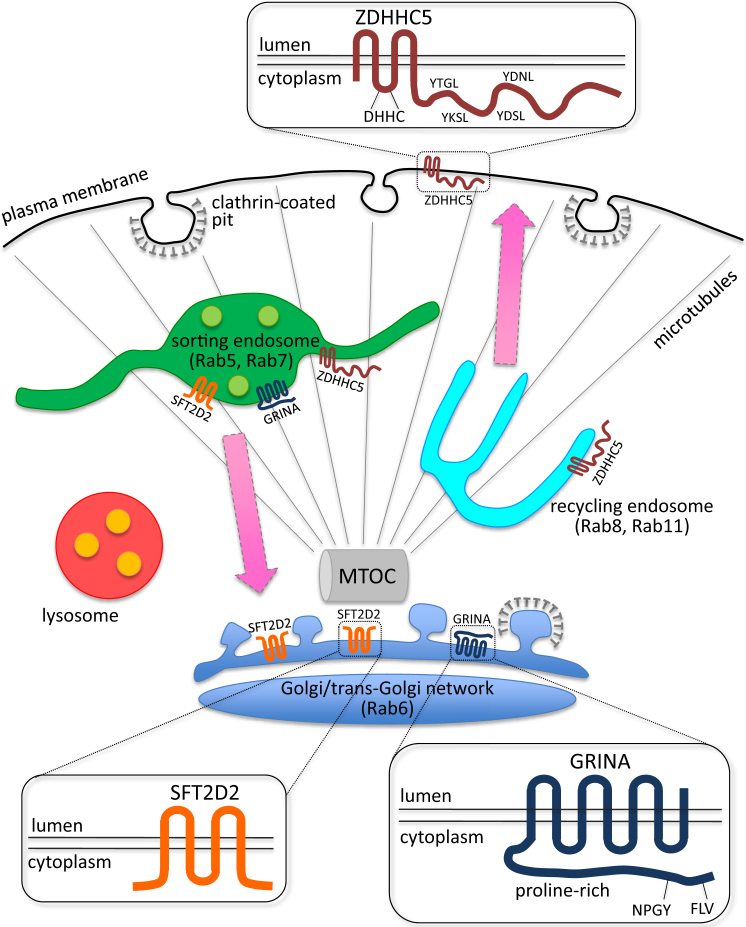
Model Schematic depiction of the localization and topology of the three multipass membrane proteins identified as regulators of endosome-to-Golgi retrieval. Features such as the YxxΦ motifs in ZDHHC5 are indicated.

GRINA is a member of the LFG (Lifeguard) family of proteins, a family of proteins highly conserved among eukaryotes with a proposed protective role in apoptosis (Hu et al., 2009), a function also reported for GRINA (Rojas-Rivera et al., 2012). We observed that GRINA-Myc localizes to the Golgi and to post-Golgi membranes, and that its expression has a dominant-negative effect on the appearance of both the Golgi and endosomes. These observations are in line with a report that GRINA induces resistance to Shiga toxin (Yamaji et al., 2010). In addition, we found that GRINA KD disrupted Golgi localization of several but not all Golgi-localized proteins and reduced the steady-state levels of a number of endosome-to-Golgi cargo proteins including TGN46. Our analyses of the three multipass membrane-spanning proteins, summarized in Figure 7, confirm their function in endosome-to-Golgi retrieval, and future studies may reveal precisely how these proteins operate in this pathway.

Other genes identified as modulating endosome-to-Golgi and reported herein are good candidates for further studies focused on the pathology of AD and PD. A recent analysis of genes encoding retromer-associated proteins identified single nucleotide polymorphisms and other variants linked with late-onset AD (Vardarajan et al., 2012). We report here that PLD3, a gene that increases risk of AD (Cruchaga et al., 2014), is required for endosome-to-Golgi retrieval and therefore establish a role for PLD3 in a pathway that is now viewed as key to events early in the pathology of AD (Small, 2008; Willnow and Andersen, 2013). Thus, other genes we report merit investigation for linkage to diseases such as AD and PD and are also worth examining for a role in the processes that underlie infection by bacterial and viral pathogens (e.g., Legionella and HPV) that exploit the endosome-to-Golgi pathway for their own ends.

## Experimental Procedures

### Screening

Using a Beckman Biomek robot, cells stably expressing CD8-CIMPR and GFP-GOLPH3 (described in Harbour et al., 2010) were seeded onto 20 μl 180 nM siGenome (for the primary screen) or ON TARGETplus (for the pilot screen and validation screens) siRNA pools in 96-well plates. After 72 hr at 37°C, cells were allowed to bind anti-CD8 antibody at room temperature for 15 min, washed, and then chased for 30 min at 37°C before fixing and immunolabeling. Images were acquired on a Cellomics Arrayscan V automated microscope and analyzed using the Cellomics vHCS:View software and its colocalization bioapplication. Data were exported for further analysis, including plate-wise normalization and SSMD calculation, in SQL and Origin.

### Characterization of SFT2D2, ZDHHC5, and GRINA

Myc-tagged human SFT2D2, ZDHHC5, and GRINA constructs were purchased from Origene and used for transient transfections in HeLa cells including into cells stably expressing GFP- or mStrawberry-tagged Rab or EHD1 constructs. Immunolabeled cells were imaged on a wide-field fluorescence microscope (Zeiss). siRNA-mediated silencing was performed using ON TARGETplus siRNA pools. Retromer assembly and interactions were investigated by immunoprecipitation using cells stably expressing VPS29-GFP and published protocols (Harbour et al., 2012). Cargo protein glycosylation changes upon protein silencing were assessed using agarose-bound wheat germ agglutinin (Sigma) precipitation followed by western blotting.

Supplemental Experimental Procedures, including screening details, hit selection criteria, and antibodies used are in Supplemental Information. Screening data not available in Tables S1, S2, S3, and S4, as well as images from any of the reported screens, are available upon request (syab2@cam.ac.uk).

## Supplemental Information

Document S1. Supplemental Experimental Procedures and Figures S1–S4

Table S1. TGN Retrieval Ratios Measured in the Pilot Screen of 310 Trafficking Genes, Related to Figure 1

Table S2. Primary Screen Data for the 1,087 Primary Screen Hits, Related to Figure 2C

Table S3. Validation Screen Results, Related to Figure 2E

Table S4. Reported Cellular Locations and Functional Information for the 88 Validated Hits, Related to Figure 2F

Document S2. Article plus Supplemental Information
