# Supplementary material for: Genome-wide RNAi Screen Reveals a Role for Multipass Membrane Proteins in Endosome-to-Golgi Retrieval
Source: Cell Rep. 2014 Nov 20;9(5):1931–45. doi: 10.1016/j.celrep.2014.10.053 (PMC4542293; doi:10.1016/j.celrep.2014.10.053)
Supplement: Document S1. Supplemental Experimental Procedures and Figures S1–S4 [file mmc1.pdf]

Cell Reports, Volume *9*

Supplemental Information

Genome-wide RNAi Screen Reveals a Role for Multipass Membrane Proteins in Endosome-to-Golgi Retrieval

Sophia Y. Breusegem and Matthew N.J. Seaman

Supplemental Information

Genome-wide RNAi screen reveals role for multi-pass membrane proteins in endosome-to-Golgi retrieval

Sophia Y. Breusegem and Matthew N. J. Seaman

Inventory of Supplemental Information:

Supplemental Data:

Figure S1: ZDHHC5-Myc localizes to the plasma membrane, endosomes, and Rab8- and Rab11-positive recycling tubules – Related to Figure 4B.

Figure S2: ZDHHC5 KD differentially affects SNARE proteins – Related to Figure 4.

Figure S3: GRINA KD perturbs Golgi protein localization; GRINA-Myc expression coalesces retromer-positive endosomes – Related to Figure 5.

Figure S4: Further characterization of retromer and retromer cargo in SFT2D2, ZDHHC5 or GRINA KD cells – Related to Figure 6.

Table S1: TGN retrieval ratios measured in pilot screen of 310 trafficking genes – Related to Figure 1.

Table S2: Primary screen data for the 1087 primary screen hits – Related to Figure 2C.

Table S3: Validation screen results – Related to Figure 2E.

Table S4: Reported cellular locations and functional information for the 88 validated hits – Related to Figure 2F.

Supplemental Experimental Procedures

Supplemental References

Supplemental Data:

Supplemental Figures and Legends:

Figure S1: ZDHHC5-Myc localizes to the plasma membrane, endosomes, and Rab8- and Rab11-positive recycling tubules – Related to Figure 4B.

(A-B) Cells stably expressing mStrawberry-Rab8a were transfected with ZDHHC5-Myc for 24 hours before fixing and staining. Transfected cells demonstrate co-localization of ZDHHC5-Myc with Rab8-positive recycling tubules and with VPS35-positive endosomes (arrows in (**B**)). The area of (**A**) magnified in (**B**) is marked by a white box. **(C)** HeLa cells were transfected with ZDHHC5-Myc for 24 hours before fixation and staining for Myc (*red*) and MICAL-L1 (*green*). Arrows indicate co-localization of ZDHHC5 and MICAL-L1, an interactor of Rab8a (Sharma et al., 2009). **(D-E)** HeLa cells stably expressing GFP-EHD1 were transfected with ZDHHC5-Myc, fixed and stained for Myc (*red*), GFP (*green*) and VPS35 (*blue*). The boxed area in (**D**) is magnified in (**E**). Arrows indicate co-localization of ZDHHC5 and EHD1, another interactor of Rab8a (Roland et al., 2007), on recycling tubules. (**F**) Cells stably expressing GFP-Rab11 were transfected with ZDHHC5-Myc for 24 hours before fixing and staining. Transfected cells show co-localization of intracellular ZDHHC5-Myc with GFP-Rab11 and with MICAL-L1 (*white* colour in overlay image). However, some ZDHHC5-positive tubules are positive for MICAL-L1 but not GFP-Rab11 (*pink* colour in overlay image). Scale bars (**A**, **C**, **D**, **F**) 20 μm, (**B**, **E**) 5 μm.

Figure S2: ZDHHC5 KD differentially affects SNARE proteins – Related to Figure 4.

(A-E) Control and ZDHHC5-silenced HeLa cells were fixed and stained for CIMPR (**A-D**, *red*) or TGN46 (**E**, *red*), GM130 (*blue*, **A-D**) and, in *green*, STX6 (**A**), STX7 (**B**), STX8 (**C**), STX10 (**D**) or STX16 (**E**). **(F)** Whole cell lysates of control and ZDHHC5-silenced HeLa

cells were separated on LDS-PAGE and blotted for the indicated proteins. ZDHHC5 silencing did not affect the total cellular level for any of the SNARE proteins investigated. **(G)** Quantification of the average intensity of the staining for the indicated syntaxin proteins in a representative set of images as in (**A-E**). ZDHHC5 knockdown significantly increased the measured average intensity of STX6, STX7 and STX8 but not of STX5 (*no images shown*), STX10 or STX16. While some of these proteins could be substrates for the palmitoyl transferase activity of ZDHHC5 (e.g. STX7 and/or STX8 (He and Linder, 2009)), others might, similar to CIMPR, depend on ZDHHC5 for normal trafficking, and thus their localization is disrupted when ZDHHC5 is silenced. Scale bar (**A-E**): 20 μm.

Figure S3: GRINA KD perturbs Golgi protein localization; GRINA-Myc expression coalesces retromer-positive endosomes – Related to Figure 5.

(A-C) Control (*top rows*) and GRINA-silenced (*bottom rows*) cells were fixed and stained for (**A**): GM130 (*red*), Golgi mannosidase II (*green*) and VPS35 (*blue*); (**B**): EEA1 (*red*) and STX5 (*green*); (**C**): TGN46 (*red*) and LAMP1 (*green*). GRINA knock-down perturbs Golgi-targeting of Mannosidase II (**A**) and decreases Golgi-localized TNG46 (**C**). In addition, GM130 staining appears fragmented (**A**) while STX5 staining is unaffected (**B**). Finally, GRINA knock-down does not change endosomal morphology of EEA1 (**B**) or VPS35 (**A**) but results in an apparent increase in lysosome size (LAMP1 staining, **C**). **(D)** HeLa cells were transfected with GRINA-Myc and stained for Myc (*red*), SNX1 (*green*) and VPS35 (*blue*). The transfected cell is indicated by an asterisk. GRINA-Myc expression coalesces SNX1- and VPS35-positive endosomes into larger round structures that contain GRINA-Myc. Scale bars (**A-D**): 20 μm.

Figure S4: Further characterization of retromer and retromer cargo in SFT2D2, ZDHHC5 and GRINA KD cells – Related to Figure 6.

(A) SFT2D2, ZDHHC5 or GRINA KD do not affect retromer assembly or interactions with the WASH complex or with TBC1D5. Control and siRNA-treated HeLa cells stably expressing VPS29-GFP were lysed and VPS29-GFP-interacting proteins were isolated by co-immunoprecipitation (co-IP). Lysates and co-IP samples were analysed by Western blotting for the indicated proteins. **(B)** GRINA KD reduces retromer cargo stability. Control and KD cells, silenced for the indicated proteins, were either left untreated or incubated for 3 hours at 37ºC with 100 μg/mL cycloheximide before lysis. Total cell lysates were separated by LDS-PAGE and blotted for the indicated proteins. **(C)** VPS35 endosomes are brighter in SFT2D2, ZDHHC5 or GRINA KD cells compared to control cells. Multiple cells (> 500) were imaged using an automated microscope and measured fluorescence intensities were quantified. SFT2D2, ZDHHC5 or GRINA KD increase the brightness of the VPS35-positive endosomes, increasing their size to a much smaller extent. ** indicates $p < 0.01$ compared to control, * indicates $p < 0.05$ compared to control.

Supplemental Tables

Table S1: TGN retrieval ratios measured in pilot screen of 310 trafficking genes – Related to Figure 1.

Table S2: Primary screen data for the 1087 primary screen hits – Related to Figure 2C.

Table S3: Validation screen results – Related to Figure 2E.

Table S4: Reported cellular locations and functional information for the 88 validated hits – Related to Figure 2F.

Supplemental Experimental Procedures

Plasmids and Plasmid Transfection

The CD8-CIMPR reporter construct was described before (Seaman, 2004) and subcloned in pIRESpuro2 (Clontech, Saint-Germain-en-Laye, France). Human GOLPH3 was PCR-amplified, cloned into pEGFP-C1 (Clontech) and subcloned in pIRESNeo2 (Clontech). Single colonies of HeLa cells stably expressing both GFP-GOLPH3 and CD8-CIMPR were selected and screened for expression. A single cell line was used for all studies presented.

Transfection-ready plasmids encoding Myc- and Flag-tagged proteins (SFT2D2, ZDHHC5, GRINA) were from Origene (Rockville, MD, USA) and purchased from Cambridge Bioscience (Cambridge, UK). Plasmids were transfected into HeLa cells using Effectene (Qiagen, Manchester, UK) according to the manufacturer's instructions, or using polyethylenimine (PEI, Polysciences Inc., Warrington, PA, USA) according to (Breusegem and Seaman, 2014). To create a cell line stably expressing SFT2D2-Myc the gene was excised from the pCMV6 vector by digestion with SgfI and FseI and inserted into a similarly digested pIRESneo2 modified by insertion of a SgfI and FseI site between its NheI and BamHI sites. Restriction enzymes were from New England Biolabs (Hitchin, UK) and ligations were done using the Rapid Ligation kit (Roche Diagnostics Ltd, Burgess Hill, UK). Constructs were verified by agarose gel electrophoresis and immunofluorescence after transient transfection. Stable cell lines were selected using geneticin (Life Technologies Ltd, Paisley, UK).

Cell culture

Mouse hybridoma cells (American Tissue Culture Collection) for the production of monoclonal anti-CD8 antibody were grown in Excell medium (Sigma-Aldrich, Gillingham, UK) supplemented with 10 mM L-glutamine (Sigma-Aldrich).

All other cell lines were maintained in DMEM/high glucose medium containing 5% fetal bovine serum (FBS), 2 mM L-glutamine, 50 units/ml penicillin and 50 μg/ml streptomycin (all from Sigma-Aldrich) (complete medium). HeLa cells stably expressing GFP-GOLPH3 and CD8-CIMPR were grown in complete medium that additionally contained 0.4 mg/mL geneticin (Life Technologies Ltd) and 1 μg/ml puromycin (Sigma-Aldrich). HeLa cells stably expressing mStrawberry-Rab8a were a gift from Andrew Peden (University of Sheffield, UK) and maintained in complete medium containing 0.4 mg/mL geneticin. HeLa cells stably expressing GFP-EHD1 were described before (Gokool et al., 2007), as were HeLa cells stably expressing GFP-tagged Rab proteins (Seaman et al., 2009) and HeLa cells stably expressing VPS29-GFP (Collins et al., 2005). To eliminate possible mycoplasma contamination cells were treated with Plasmocin (Invivogen, San Diego, CA, USA) before use in screening.

Mini-library screens

Six mini-libraries of ON TARGETplus™ siRNA pools (4 oligos/gene, Dharmacon, Thermo Fisher Scientific, Waltham, MA, USA) targeting genes homologous to yeast *VPS* proteins, SNARE protein genes, candidate endocytosis genes or kinesin, dynein or myosin motor protein genes were arrayed into the central wells of V-bottomed 96-well plates (Nunc, Thermo Fisher) and stored at -20°C until ready to use. On a day of screening plates containing 10 μL 1 μM siRNA pool (in siRNA buffer, Thermo Fisher) in the central wells were thawed to room temperature and spun at 5,000 x g for 5 minutes. SiRNA oligos

targeting the retromer proteins VPS26 and SNX1 (obtained from Dharmacon and described in (Gokool et al., 2007)) were added to selected outer wells of the plates as positive controls, while some wells only contained siRNA buffer. Oligofectamine (Life Technologies) was diluted 1:10 in Optimem (Life Technologies) and equilibrated at room temperature for 8 minutes. The siRNA pools were diluted by the addition of 36 μL Optimem to each well. Next 9 μL of the Oligofectamine dilution was added to each well, and siRNA:Oligofectamine complexes were allowed to form for 20 minutes at room temperature. Meanwhile HeLa cells stably expressing CD8-CIMPR and GFP-GOLPH3 were lifted off a 75 cm^2 flask and counted using a CASY cell counter (Roche, Basel, Switzerland). The siRNA:Oligofectamine complexes were aliquoted in 2 flat-bottomed assay plates (Corning, Amsterdam, The Netherlands), each containing 20 μL/well. Cells were diluted in complete medium to 60,000 cells/mL, and 100 μL of this cell suspension was added to each assay plate well. The plates were vortexed briefly and incubated at 37ºC for 72 hours.

Trafficking of CD8-CIMPR was then assessed using the anti-CD8 antibody uptake assay depicted in **Figure 1A**. The cell culture medium was flicked out of the plates and cells were washed once with room temperature PBS (100 μL/well) before incubation with anti-CD8 monoclonal antibody (hybridoma cell culture supernatant diluted 1:5 in complete cell culture medium, 40 μL/well) for 15 minutes at room temperature. Unbound antibody was removed by quickly washing the cells with 50 μL/well PBS and 100 μL/well pre-warmed (37°C) complete medium was then added for a 30-minute antibody chase at 37ºC. All subsequent steps were carried out at room temperature. At the end of the antibody chase period the cells were washed again with PBS before fixation with 50 μL/well 4% paraformaldehyde (PFA, Polysciences Inc., Warrington, PA, USA) in PBS for 10 minutes. Cells were permeabilized with 50 μL/well 0.1% TX-100 (Sigma-Aldrich) in PBS for 10 minutes. Unspecific binding of

antibodies was blocked by incubation for 30 minutes with 3% BSA (Fisher Scientific UK, Loughborough, UK) in PBS (IF buffer). Rabbit anti-GFP (described in (Seaman et al., 2009)) was added at 1:1,000 in IF buffer for 1 hour. Following 2 washes with PBS cells were incubated with Alexa Fluor 488 anti-rabbit antibody and Alexa Fluor 555 anti-mouse IgG_{2a} antibody (each diluted 1:2,000 in IF buffer) for 1 hour. Cells were washed twice with PBS before incubation for 30 minutes with Whole Cell Stain blue (Cellomics®, Thermo Fisher) diluted 1:1,000 in PBS. After 2 final washes with PBS cells were stored with 150 µL/well PBS at 4°C until imaging could be performed.

Cells were imaged on a Cellomics® Arrayscan V^{TI} automated microscope using the Colocalization Bio-application in the Arrayscan software. Per well at least 250 cells ("objects" defined by the whole cell stain) were imaged unless the "sparse well" criterion (8 consecutive field with less than 2 selected objects) was reached. The whole cell stain, Alexa Fluor 488 and Alexa Fluor 555 images were acquired sequentially using a single multi-pass filter set. Images were stored and further analysed in the Cellomics® vHCS™:View software. Regions of interest (ROIs) A and B as indicated in **Figure 1B** were defined in the Colocalization bio-application and derived from the GFP-GOLPH3 and whole cell stain images, respectively. The TGN retrieval ratio as defined in **Figure 1B** was directly calculated in the software for each cell and averaged over all cells selected in each well. Average TGN retrieval ratio values were imported into Origin software (OriginLab Corporation, Northampton, MA, USA) for further analysis and graphical presentation.

Each mini-library was screened at least 2 times in duplicate. From the replicate measurements 20 genes were selected whose knockdown reproducibly reduced the TGN retrieval ratio to values equal to or lower than the average TGN retrieval ratio measured for the SNX1 siRNA positive control. For these 20 genes the sequences making up the ON TARGETplus™ siRNA

pool were ordered as individual ON TARGETplus^{TM} oligos (Dharmacon) and arrayed in columns 3 to 12 of a 96-well plate. Positive (SNX1 siRNA) and negative control wells were arrayed as in **Figure 2A**. Cells expressing GFP-GOLPH3 and CD8-CIMPR were reverse transfected with siRNA using the same protocol as for the ON TARGETplus^{TM} siRNA pool transfection. The anti-CD8 antibody uptake assay was also as described above and repeated twice in duplicate. Results of all mini-library screens are in **Table S1**.

Genome-wide siRNA screen

21,121 siRNA smartpools (siGenome, Dharmacon/Thermo Scientific) were arrayed onto 267 ninety-six-well plates and stored in 10 μL 1 μM aliquots at -20ºC. On a day of transfection siRNAs were thawed to room temperature and spun at 5,000 x g for 5 minutes. Cells stably expressing GFP-GOLPH3 and CD8-CIMPR were reverse transfected as in the mini-library screens, except that 8,000 cells were seeded per well and all steps involving 96-well plates were carried out using a robotic liquid handling system (BiomekNX, Beckman Coulter, High Wycombe, UK) enclosed in a in a custom-made enclosure with airflow technology and a UV lamp (BigNeat Containment Technology, Hampshire). Only cells that were passaged less than 10 times were used. Anti-CD8 antibody uptake was also carried out as described for the mini-library screens, using a bench-top semi-automated liquid dispenser system (Matrix Wellmate, Thermo Scientific) to aid in the antibody-uptake steps as well as in the fixing and staining steps.

Primary hit selection

Several acquired data as well as values calculated by the iView software were exported, assembled and linked to the plate layouts in a SQL database before being imported in Origin for statistical analysis and graphical presentation. TGN retrieval ratios were normalized plate-by-plate, such that the average TGN retrieval ratio for the negative control wells on each pate

equalled the average value measured across the entire screen (0.65). Hit selection was based on calculated SSMD values. SSMD values, defined in (Zhang, 2007), measure the magnitude of the difference between an siRNA of interest and a negative reference: $SSMD = \frac{\mu_i - \mu_N}{\sqrt{\sigma_i^2 + \sigma_N^2}}$, where μ_i and σ_i are the mean, respectively standard deviation, for an siRNA of interest and μ_N and σ_N the mean, respectively standard deviation for the negative control. We calculated SSMD values for the normalized TGN retrieval ratio as well as for the anti-CD8 intensity in the cytoplasm outside the GOLPH3-defined TGN mask. Primary hits were defined as having absolute values for these 2 SSMD values larger than 3 (i.e. very strong effects *vs*. the negative control), as well as fulfilling the following additional criteria: i) at least 40 cells were measured on each plate, with at least one measurement requiring less than 50 fields (i.e. siRNA is non-toxic and does not target a gene necessary for cell division); ii) the siRNA pool targets a single gene, taking into account re-annotation of the original siGenome pools by M. Boutros (DKFZ, using RefSeq25); and iii) for targets represented on an analysed microarray only detected ones were retained as hits. These criteria yielded 1106 primary hits, of which 1087 were still retained in the NCBI database.

To select hits for secondary screening an additional SSMD was calculated for the total cellular anti-CD8 signal to exclude siRNAs that cause a secretion defect (and therefore have less CD8-CIMPR at the plasma membrane at any one time) instead of an endosome-to-Golgi trafficking defect. This intensity criterion yielded 389 very strong hits which were visually inspected to assess morphology, anti-CD8 signal intensity distribution, cell polarization and cell size. In addition, manual inspection was also done for primary hits associated with the gene ontology terms intracellular trafficking, cytoskeleton, Golgi or GTPase activity. In this way 360 genes were selected for follow-up using ON TARGETplus™ siRNA SMARTpools.

Table S2 assembles relevant data acquired for the 1087 primary screen hits as well as the 3 calculated SSMD values used in hit selection.

Validation screen

ON TARGETplus^{TM} siRNA SMARTpools in siRNA buffer were arrayed in the central wells of 96-well plates in 5 μL or 10 μL 1 μM aliquots and stored at -20ºC until use. Reverse transfection of cells with siRNA and anti-CD8 antibody uptake assay were as in the primary screen except that the ratio siRNA:Oligofectamine was decreased by ½ and additional negative (RISC-free and non-targeting) and positive (Rab7 and VPS26) siRNAs were included (see **Figure 2D**).

Small scale siRNA knockdown

For silencing of selected genes the cells of choice were seeded into 6-well plates to 30-40% confluency. Sixteen hours later cells were transfected with ON TARGETplus^{TM} siRNA SMARTpools, or, in the case of SNX1, a single ON TARGETplus^{TM} siRNA oligo. For each well to be transfected 10 μL Oligofectamine was diluted into 20 μL Optimem and left at r.t. for 5-10 minutes. 5 μL of a 20 μM siRNA stock solution was diluted in 165 μL Optimem. The diluted Oligofectamine solution was then added to the diluted siRNA solution and left for 20 minutes at r.t. Cells were washed once with Optimem. siRNA:Oligofectamine complexes were further diluted with 0.8 mL Optimem. The final 1 mL siRNA:Oligofectamine was added drop-wise to the cells. Four hours later 1 mL complete medium containing 20% FBS was added to each well. After 24 hours at 37ºC the medium was replaced with regular complete medium and cells were further incubated at 37ºC. Cells were assayed 72 hours after siRNA transfection. When required cells were trypsinised and seeded onto clean coverslips 48 hours after transfection.

Antibodies and Reagents

Anti-CD8 monoclonal antibody was obtained from hybridoma cell culture supernatant. Rabbit polyclonal antibodies against GFP and against human VPS26, TGN46 and SNX1 were generated in house and are described in previous publications (Seaman, 2004; Seaman et al., 2009). Rabbit polyclonal anti-SFT2D2 antibody was from Abcam (Cambridge, UK). Rabbit polyclonal anti-ZDHHC5 antibodies were obtained from Abcam, Sigma and Protein Tech Group Inc (Manchester, UK), with the Sigma antibody being used for immunofluorescence. Other antibodies used in immunofluorescence: monoclonal anti-GFP (Life Technologies), monoclonal anti-SNX1, anti-EEA1, anti-GM130 and anti-α5-integrin (all from BD Biosciences, Oxford, UK), rabbit polyclonal anti-Mannosidase II (Millipore/Chemicon, Watford, UK), rabbit polyclonal and mouse monoclonal anti-Myc, monoclonal anti-VPS35 and anti-LAMP1 (all from Santa Cruz Biotechnology Inc., Heidelberg, Germany), monoclonal anti-Flag (clone M2) and rabbit anti-GLG1 (both from Sigma), monoclonal anti-CIMPR and anti-β1-integrin and rabbit anti-STX16 (Abcam), rabbit anti-STX5 (Synaptic Systems, Goettingen, Germany), monoclonal anti-MICAL-L1 (Novus Biologicals, Cambridge, UK), rabbit anti-STX6, anti-STX7, anti-STX8, anti-STX10, anti-VAMP3, anti-VAMP7, anti-VAMP8, and mouse anti-STX16 (all generous gifts from Andrew Peden, University of Sheffield, UK). Other antibodies used for Western blotting: rabbit polyclonal anti-CIMPR (a generous gift from Paul Luzio, University of Cambridge, UK), monoclonal anti-transferrin receptor (Life Technologies), rabbit polyclonal anti-actin and monoclonal anti-tubulin (both from Sigma), rabbit anti-FAM21, rabbit anti-strumpellin and monoclonal anti-TBC1D5 (all from Santa Cruz Biotechnology Inc.). Alexa Fluor 488, Alexa Fluor 555 or Alexa Fluor 647 labelled anti-mouse and anti-rabbit antibodies were obtained from Life Technologies, including isotope-specific goat anti-mouse IgG_1, IgG_{2a} or IgG_{2b} antibodies. Horse radish peroxidase-conjugated antibodies were from Sigma.

Immunofluorescence

In preparation for immunofluorescent staining cells on 22 mm square coverslips in 6-well plates were washed once with PBS and fixed in 4% paraformaldehyde (Polysciences Inc.) in PBS for 10 minutes at room temperature. Cells were then permeabilized for 10 minutes in 0.1% TX-100 (Sigma) in PBS. Next, unspecific antibody binding was blocked by incubation for 20 minutes in IF buffer (see *Mini-library screens*). Coverslips were then incubated sequentially with the primary and secondary antibodies diluted in IF buffer at room temperature for 1 hour each, with three 5-minute washes with PBS in between. After 3 final washes with PBS the coverslips were mounted using Prolong Gold (Life Technologies). This permeabilization, blocking and staining protocol was followed for all antibodies except when staining for SNARE proteins, in which case each of these steps were done in PBS buffer containing 0.1% saponin (Sigma-Aldrich) and 5% fetal bovine serum. Immunolabeled cells were imaged at room temperature using a 63x oil immersion 1.4 N.A. objective on a ZeissAxiovert epifluorescence microscope equipped with a Hamamatsu ORCA-R2 CCD camera, using Simple PCI6 acquisition software. Images were processed in Zeiss LSM Browser software, using the range indicator display option to adjust background and displayed intensity range, and applying identical adjustments to all images to be compared. Some immunofluorescence images were acquired on a Zeiss Axioimager epifluorescence microscope equipped with an ORCA Flash 4 camera, using a 63x 1.4 N.A. oil immersion objective and the Zeiss ZenBlue software.

For the co-localization analysis in **Figure S2** images were opened in ImageJ software (National Institutes of Health) and Pearson’s correlation coefficients calculated using the Just Another Colocalization Plugin (JACoP). Average intensity measurements were also made in ImageJ after applying a constant intensity threshold to all images to be analysed. For the co-localization and intensity analyses in **Figures 3**, **6** and **S4-C** cells were seeded and stained in

24-well plates and images were acquired on the Cellomics Arrayscan automated microscope using the co-localization bio-application. For each experimental condition at least 500 cells were imaged, and, when possible, technical replicates were included. Co-localization and intensity parameters were obtained from analysis in the Cellomics® vHCS™:View software. As in the anti-CD8 uptake assay, a whole cell stain was used to define the cellular outline.

Western Blotting

To prepare total cell lysates for Western blotting cells grown to confluency in 6-well plates were washed once with ice-cold PBS and then scraped in 100 μL/well 1% TX-100 in PBS. Alternatively, cell lysates were prepared as part of an immunoprecipitation or lectin pulldown protocol (*see below*). Lysates were reduced using DTT and denatured in LDS PAGE buffer (Life Technologies) at 85°C for 5 minutes before loading onto 4-12% NuPage Bis-Tris gels (Life Technologies). After electrophoretic separation at 150V for 70 minutes proteins were transferred onto nitrocellulose membranes. After overnight blocking with 5% milk in Tris-buffered saline buffer containing 0.1% Tween (TBST) membranes were probed with primary antibodies at room temperature for $1^{1/2}$ to 2 hours, followed by 3 washes with TBST/milk and incubation for 1 hour with HRP-conjugated secondary antibodies. After extensive washing of excess antibodies the proteins were visualized using luminescence detection reagents (GE Healthcare) and film.

Lectin Pull-Down

HeLa cells were seeded in 100 mm dishes at ~30-40% confluency. The next day cells were transfected with siRNA using Oligofectamine. For each dish 50 μL Oligofectamine was diluted with 100 μL Optimem and equilibrated for 5 minutes. 10 μL 20 μM siRNA was diluted in 850 μL Optimem before addition of 140 μL of the Optimem/Oligofectamine dilution. siRNA: Oligofectamine complexes were allowed to equilibrate for 20 minutes at r.t.

The siRNA: oligofectamine complexes were then diluted by the addition of 4 mL Optimem and added to the cells (after 1 wash of the cells with Optimem). After a 4 hour incubation at 37ºC 5 mL complete medium containing 20% FBS was added to each dish before further incubation at 37°C. Twenty-four hours later cells were trypsinized and seeded into 140 mm dishes. Seventy-two hours after the siRNA transfection the cells were washed once with PBS before incubation with 10 mL 0.1 mg/mL cycloheximide for 3 hours to halt new protein synthesis. Next, cells were washed once with ice-cold PBS and lysed in ice-cold lysis buffer (1% Triton X-100 in PBS containing 1 Complete™ protease inhibitor tablet (Roche) per 50 mL). Insoluble material was removed by centrifugation at 10,000 x g for 5 minutes at 4°C. Lysates were pre-cleared by rotating for 30 minutes at 4ºC in the presence of 50 μL sepharose-bound protein A. After removing the sepharose by centrifugation the lysates were incubated with 40 μL of a suspension of wheat germ agglutinin (WGA) conjugated to agarose (Sigma) for 2 hours at 4°C. The WGA-agarose beads were washed 4 x with lysis buffer and stored at -20°C until further processing. For analysis by Western blotting beads were vortexed in 1x LDS PAGE loading buffer before Western blotting as above.

Native Immunoprecipitation

HeLa cells stably expressing VPS29-GFP were seeded in 140 mm dishes and transfected at ~30-40% confluency with siRNA using Oligofectamine as described above for the siRNA transfections in the lectin pulldown experiments but using 2.5 x the amounts of siRNA, Oligofectamine and Optimem. After 24 hours cells were trypsinized and seeded into two 140 mm dishes. Seventy-two hours after siRNA transfection VPS29-GFP complexes were immunoprecipitated using a previously used lysis buffer and protocol, both recently detailed in (Breusegem and Seaman, 2014).

Supplemental References

Breusegem, S.Y., and Seaman, M.N. (2014). Image-based and biochemical assays to investigate endosomal protein sorting. Methods in enzymology *534*, 155-178.

Collins, B.M., Skinner, C.F., Watson, P.J., Seaman, M.N., and Owen, D.J. (2005). Vps29 has a phosphoesterase fold that acts as a protein interaction scaffold for retromer assembly. Nat Struct Mol Biol *12*, 594-602.

Gokool, S., Tattersall, D., and Seaman, M.N. (2007). EHD1 interacts with retromer to stabilize SNX1 tubules and facilitate endosome-to-Golgi retrieval. Traffic *8*, 1873-1886.

He, Y., and Linder, M.E. (2009). Differential palmitoylation of the endosomal SNAREs syntaxin 7 and syntaxin 8. J. Lipid Res. *50*, 398-404.

Roland, J.T., Kenworthy, A.K., Peranen, J., Caplan, S., and Goldenring, J.R. (2007). Myosin Vb interacts with Rab8a on a tubular network containing EHD1 and EHD3. Mol. Biol. Cell *18*, 2828-2837.

Seaman, M.N. (2004). Cargo-selective endosomal sorting for retrieval to the Golgi requires retromer. J Cell Biol *165*, 111-122.

Seaman, M.N., Harbour, M.E., Tattersall, D., Read, E., and Bright, N. (2009). Membrane recruitment of the cargo-selective retromer subcomplex is catalysed by the small GTPase Rab7 and inhibited by the Rab-GAP TBC1D5. J Cell Sci *122*, 2371-2382.

Sharma, M., Giridharan, S.S., Rahajeng, J., Naslavsky, N., and Caplan, S. (2009). MICAL-L1 links EHD1 to tubular recycling endosomes and regulates receptor recycling. Mol. Biol. Cell *20*, 5181-5194.

Zhang, X.D. (2007). A pair of new statistical parameters for quality control in RNA interference high-throughput screening assays. Genomics *89*, 552-561.

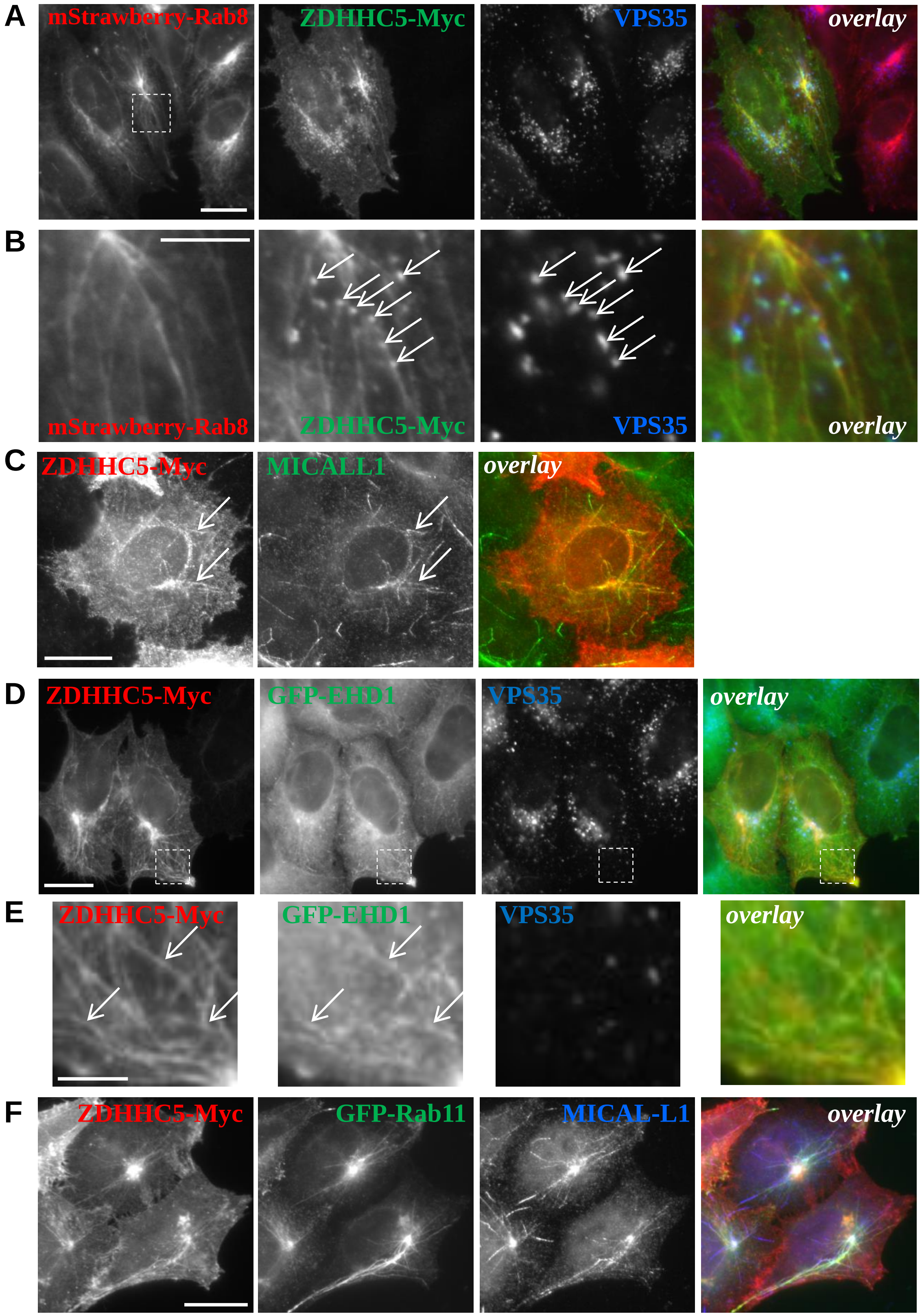
A
mStrawberry-Rab8
ZDHHC5-Myc
VPS35
overlay
B
mStrawberry-Rab8
ZDHHC5-Myc
VPS35
overlay
C
ZDHHC5-Myc
MICALL1
overlay
D
ZDHHC5-Myc
GFP-EHD1
VPS35
overlay
E
ZDHHC5-Myc
GFP-EHD1
VPS35
overlay
F
ZDHHC5-Myc
GFP-Rab11
MICAL-L1
overlay

Figure S1

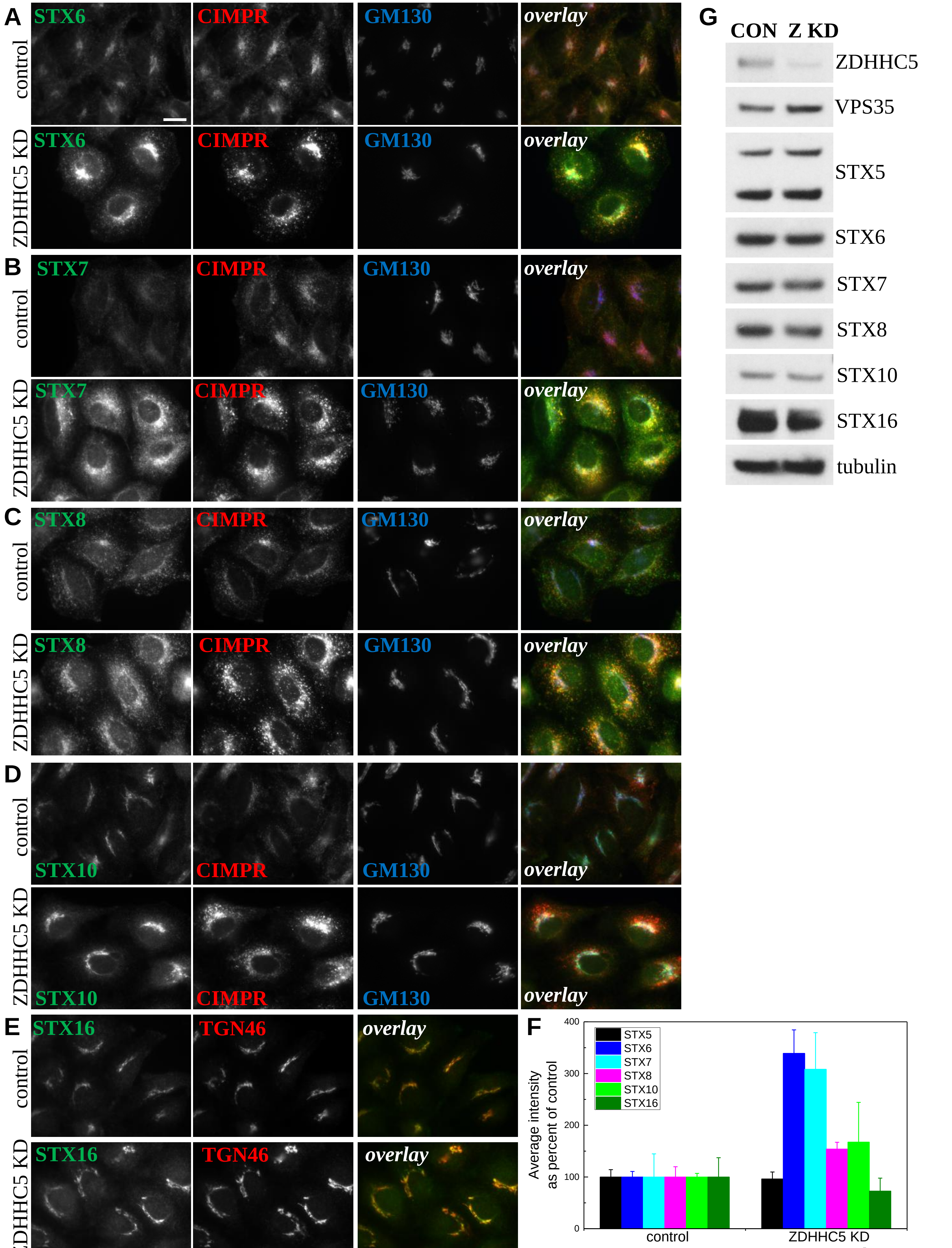
A
control
ZDHHC5 KD
STX6
CIMPR
GM130
overlay
B
STX7
C
STX8
D
STX10
E
STX16
TGN46
F
Average intensity as percent of control
STX5
STX6
STX7
STX8
STX10
STX16
400
300
200
100
0
control
ZDHHC5 KD
G
CON
Z KD
ZDHHC5
VPS35
STX5
STX6
STX7
STX8
STX10
STX16
tubulin

Figure S2

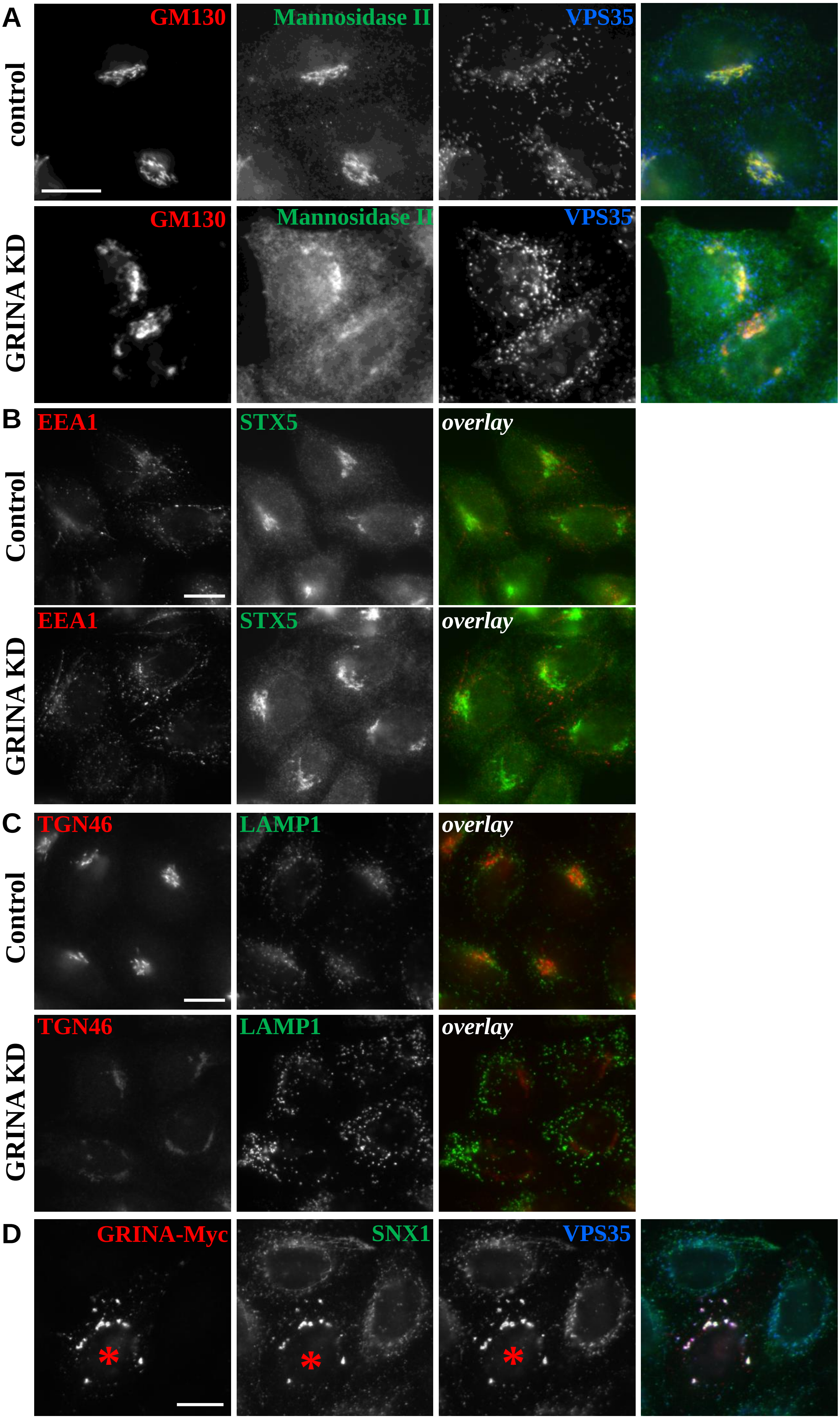
A
control
GM130
Mannosidase II
VPS35
GRINA KD
GM130
Mannosidase II
VPS35
B
Control
EEA1
STX5
overlay
GRINA KD
EEA1
STX5
overlay
C
Control
TGN46
LAMP1
overlay
GRINA KD
TGN46
LAMP1
overlay
D
GRINA-Myc
SNX1
VPS35

Figure S3

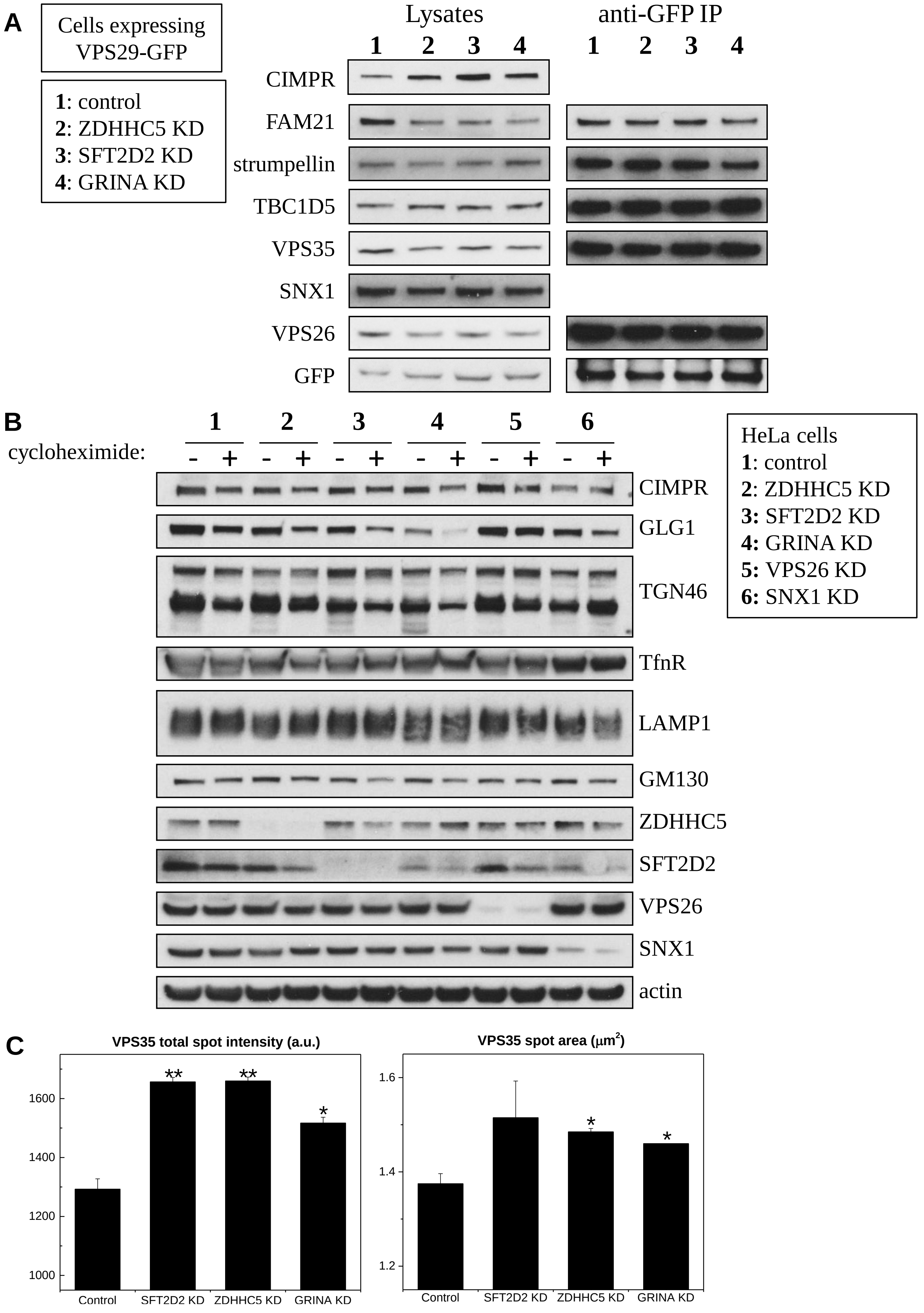
A
Cells expressing VPS29-GFP
1: control
2: ZDHHC5 KD
3: SFT2D2 KD
4: GRINA KD
Lysates
anti-GFP IP
1 2 3 4
1 2 3 4
CIMPR
FAM21
strumpellin
TBC1D5
VPS35
SNX1
VPS26
GFP
B
1 2 3 4 5 6
cycloheximide:
- + - + - + - + - + - +
CIMPR
GLG1
TGN46
TfnR
LAMP1
GM130
ZDHHC5
SFT2D2
VPS26
SNX1
actin
HeLa cells
1: control
2: ZDHHC5 KD
3: SFT2D2 KD
4: GRINA KD
5: VPS26 KD
6: SNX1 KD
C
VPS35 total spot intensity (a.u.)
1000
1200
1400
1600
**
**
*
Control
SFT2D2 KD
ZDHHC5 KD
GRINA KD
VPS35 spot area (μm2)
1.2
1.4
1.6
*
*
Control
SFT2D2 KD
ZDHHC5 KD
GRINA KD

Figure S4
