# Supplementary material for: Genome-wide RNAi Screen Reveals a Role for Multipass Membrane Proteins in Endosome-to-Golgi Retrieval
Source: Cell Rep. 2014 Nov 20;9(5):1931–45. doi: 10.1016/j.celrep.2014.10.053 (PMC4542293; doi:10.1016/j.celrep.2014.10.053)
Supplement: Document S2. Article plus Supplemental Information [file mmc6.pdf]

Cell Reports

Resource

Genome-wide RNAi Screen Reveals a Role for Multipass Membrane Proteins in Endosome-to-Golgi Retrieval

Graphical Abstract

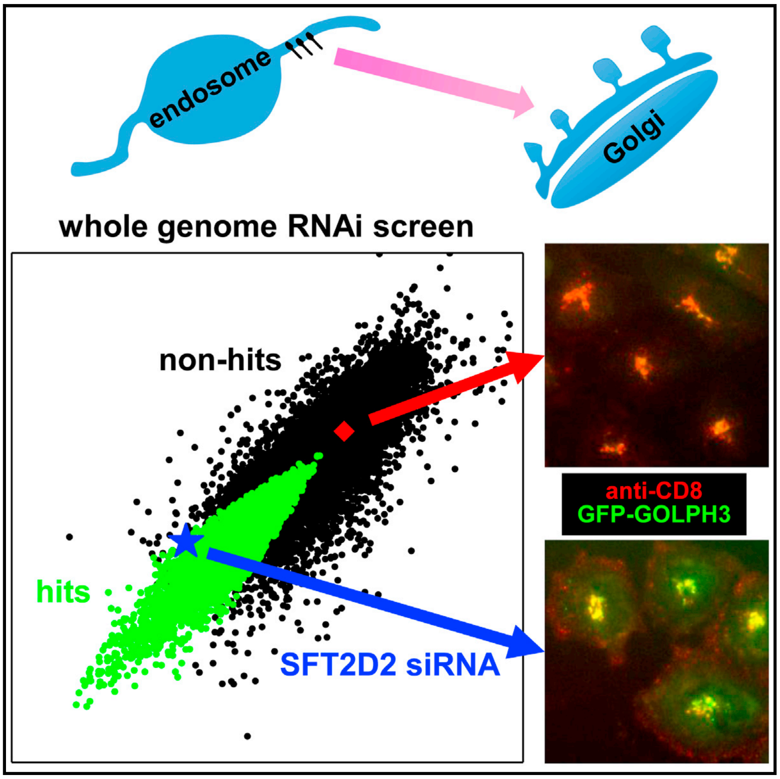

Highlights

Endosome-to-Golgi retrieval is critical for diverse physiological processes

We carried out a genome-wide siRNA screen for regulators of this pathway

We report 88 genes whose knockdown inhibits endosome-to-Golgi retrieval

We verify a role for three multipass membrane-spanning proteins in this pathway

Authors

Sophia Y. Breusegem, Matthew N.J. Seaman

Correspondence

syab2@cam.ac.uk (S.Y.B.),
mnjs100@cam.ac.uk (M.N.J.S.)

In Brief

Breusegem et al. perform an imaging-based genome-wide siRNA screen for proteins required for endosome-to-Golgi retrieval of the cation-independent mannose-6-phosphate receptor. Their hits include several multipass membrane proteins, a class of proteins often overlooked in intracellular trafficking. Among the hits, they have characterized SFT2D2, ZDHHC5, and GRINA in endosome-to-Golgi retrieval.

Breusegem & Seaman, 2014, Cell Reports 9, 1931–1945
December 11, 2014 ©2014 The Authors
http://dx.doi.org/10.1016/j.celrep.2014.10.053

OPEN ACCESS
CellPress

Genome-wide RNAi Screen Reveals a Role for Multipass Membrane Proteins in Endosome-to-Golgi Retrieval

Sophia Y. Breusegem[1,*] and Matthew N.J. Seaman[1,*]
[1]Department of Clinical Biochemistry, Cambridge Institute for Medical Research, University of Cambridge, Wellcome Trust/MRC Building, Cambridge Biomedical Campus, Hills Road, Cambridge CB2 0XY, UK
*Correspondence: syab2@cam.ac.uk (S.Y.B.), mnjs100@cam.ac.uk (M.N.J.S.)
http://dx.doi.org/10.1016/j.celrep.2014.10.053
This is an open access article under the CC BY license (http://creativecommons.org/licenses/by/3.0/).

SUMMARY

Endosome-to-Golgi retrieval is an essential membrane trafficking pathway required for many important physiological processes and linked to neurodegenerative disease and infection by bacterial and viral pathogens. The prototypical cargo protein for this pathway is the cation-independent mannose 6-phosphate receptor (CIMPR), which delivers lysosomal hydrolases to endosomes. Efficient retrieval of CIMPR to the Golgi requires the retromer complex, but other aspects of the endosome-to-Golgi retrieval pathway are poorly understood. Employing an image-based antibody-uptake assay, we conducted a genome-wide RNAi loss-of-function screen for novel regulators of this trafficking pathway and report ~90 genes that are required for endosome-to-Golgi retrieval of a CD8-CIMPR reporter protein. Among these regulators of endosome-to-Golgi retrieval are a number of multipass membrane-spanning proteins, a class of proteins often overlooked with respect to a role in membrane trafficking. We further demonstrate a role for three multipass membrane proteins, SFT2D2, ZDHHC5, and GRINA, in endosome-to-Golgi retrieval.

INTRODUCTION

The endosome-to-Golgi retrieval pathway is conserved across all eukaryotes, sorting a diverse set of cargo proteins that operate in lysosome biogenesis, iron homeostasis, polarity generation, and other essential cellular functions. The pathway is also at the epicenter of many pathogenic events including Alzheimer's disease (AD), Parkinson's disease (PD), and bacterial and viral infections. This universally conserved trafficking route functions to maintain a diverse array of membrane proteins at the Golgi. Possibly the best characterized cargo proteins for the endosome-to-Golgi pathway are the lysosomal or vacuolar hydrolase sorting receptors that mediate the transport of acid hydrolases required for lysosomal and vacuole-mediated degradation.

An essential regulator of this pathway is the retromer complex, which was first described in budding yeast (Seaman et al., 1998) and is conserved across all eukaryotes (Arighi et al., 2004; Carlton et al., 2004; Koumandou et al., 2011; Seaman, 2004). Its prototypical cargo includes the hydrolase receptors, particularly the cation-independent mannose-6-phosphate receptor (CIMPR), and it is also required for localization of the TGN marker protein TGN46, the Wnt transporter Wntless (Belenkaya et al., 2008; Yang et al., 2008), and SorL1, a member of the Vps10-domain-containing family (Fjorback et al., 2012; Nielsen et al., 2007). This interaction may therefore be relevant in AD, because SorL1 interacts with amyloid precursor protein (APP) to regulate its processing via the endosome-to-Golgi pathway, and loss of SorL1 or loss of retromer function can increase amyloidogenic processing of APP to the AD-causing Aβ form (reviewed in Fjorback and Andersen, 2012; Small, 2008; Willnow and Andersen, 2013). Some pathogens have also evolved to exploit retromer and/or endosome-to-Golgi retrieval to their own ends. For example, the human papilloma virus (HPV), following entry into the cell, interacts with retromer and is directed into an endosome-to-Golgi pathway that is believed to contribute to viral propagation within the host cell (Lipovsky et al., 2013). Furthermore, Shiga toxin produced by Shigella bacteria also utilizes retromer-mediated endosome-to-Golgi retrieval after uptake (Popoff et al., 2007). For Shiga toxin, retromer-mediated endosome-to-Golgi retrieval facilitates access to first the Golgi and then the endoplasmic reticulum where the toxin can exert its cytotoxic effects. Thus, understanding how retromer-mediated trafficking is controlled has broad implications in development and disease.

The retromer complex comprises two distinct functional units: the cargo-selective complex (CSC), which is a trimer of the Vps35, Vps29, and Vps26 proteins and a membrane-bending sorting nexin (Snx) dimer that can tubulate membranes to generate a transport intermediate (reviewed in Bonifacino and Hurley, 2008; Seaman, 2005, 2012). Although it is essential for efficient endosome-to-Golgi retrieval, retromer does not operate in isolation. The Snx dimer component of retromer (comprising SNX1 or SNX2 with SNX5 or SNX6) that mediates tubule formation also links to the microtubule cytoskeleton through interactions with p150 glued (Hong et al., 2009; Wassmer et al., 2009). These tubules are stabilized by EH-domain-containing proteins EHD1 and EHD3 (Gokool et al., 2007; McKenzie et al.,

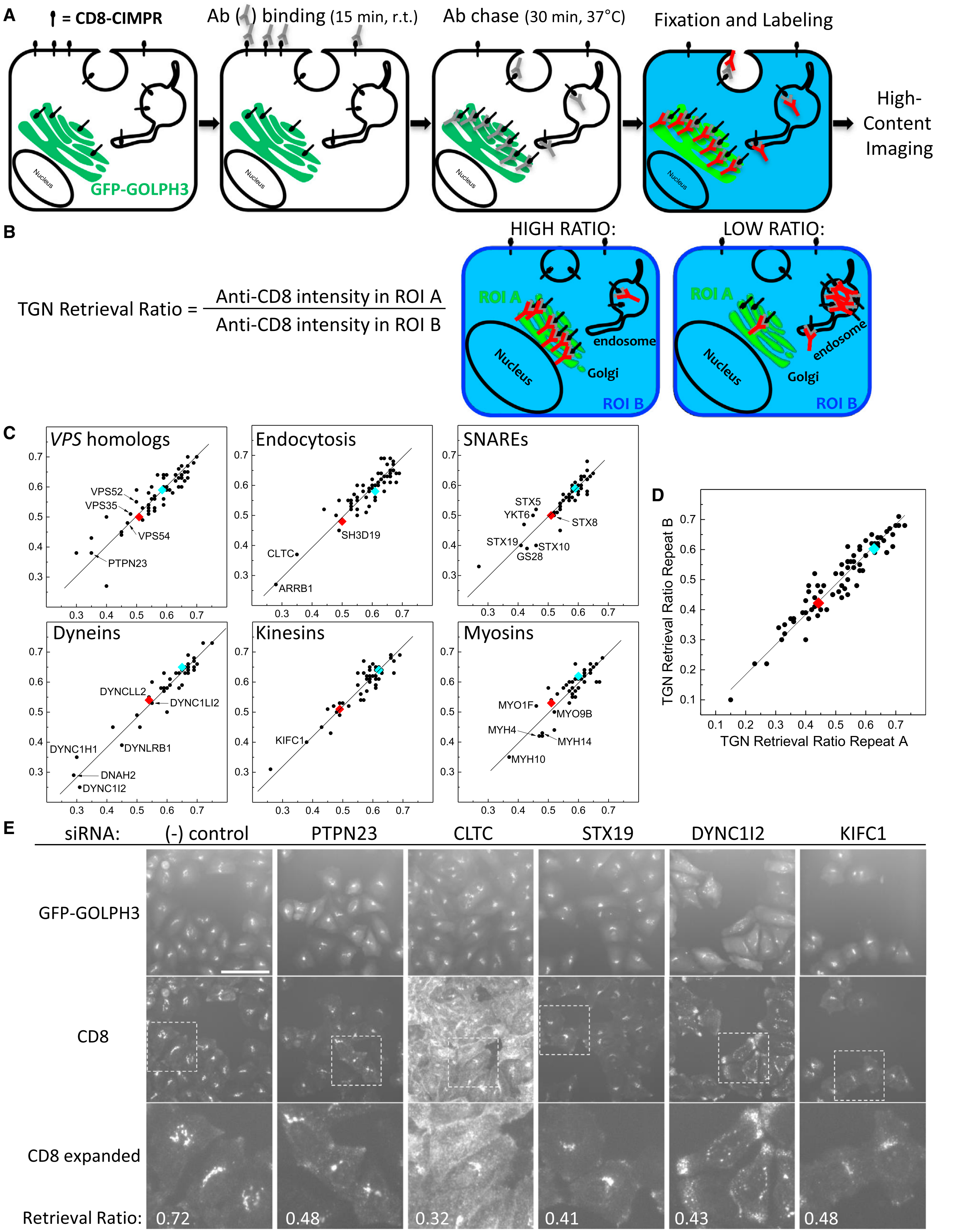

(legend on next page)

2012; Naslavsky et al., 2009). Retromer also associates with the Arp2/3-activating WASH complex that generates branched actin patches on endosomes (Harbour et al., 2010; Derivery et al., 2009; Gomez and Billadeau, 2009; and reviewed in Seaman et al., 2013). Finally, its activity is regulated by the small GTPase Rab7a, which mediates membrane recruitment of the retromer CSC (Rojas et al., 2008; Seaman et al., 2009).

Aside from retromer, only a few other factors have been linked to this trafficking pathway (including the SNARE proteins syntaxins 5, 6, 10, and 16 that mediate membrane fusion events; Ganley et al., 2008; Mallard et al., 2002; Tai et al., 2004). We therefore hypothesized that there will be many other uncharacterized components of the endosome-to-Golgi retrieval pathway, including proteins that act during retromer-mediated sorting, or independently of retromer. To address this, we have undertaken a genome-wide small interfering RNA (siRNA) screen for genes that affect endosome-to-Golgi trafficking. We have identified ~90 genes that, when silenced, result in reduced endosome-to-Golgi retrieval. These include kinases, phosphatases, cytoskeleton-associated proteins, as well as several factors that have been linked to PD. Notably, several of the genes encode multipass membrane-spanning proteins—a class of proteins often overlooked with respect to a role in membrane trafficking. We have characterized three of these multipass membrane proteins, SFT2D2, ZDHHC5, and GRINA, to further verify their function in endosome-to-Golgi retrieval.

RESULTS

Anti-CD8 Antibody Uptake Assay for siRNA Screening

CIMPR is a prototypical cargo for retromer-mediated retrieval in the endosome-to-Golgi pathway. It binds hydrolases at the *trans*-Golgi network (TGN), is packaged into clathrin-coated vesicles for delivery to endosomes, and is then recycled back to the Golgi by retromer for further rounds of hydrolase sorting. We and others have used HeLa cells stably expressing a CD8-CIMPR chimera in combination with immunofluorescence to assay the trafficking routes and protein interaction partners of the CIMPR cargo protein (Carlton et al., 2004; Harasaki et al., 2005; Seaman, 2004; Wassmer et al., 2007). In particular, we have shown that the efficient retrieval of the CD8-CIMPR reporter from endosomes to the Golgi requires retromer (Seaman, 2004). In addition, we have used a cell line stably expressing both CD8-CIMPR and GFP-tagged GOLPH3, a peripheral Golgi protein (Wu et al., 2000), to evaluate the role of retromer-interacting proteins (e.g., members of the WASH complex, TBC1D5, SNX3, and Rab7a) in endosome-to-Golgi retrieval of the CD8-CIMPR reporter (Harbour et al., 2010).

We therefore utilized the CD8-CIMPR reporter in a genome-wide siRNA screen to uncover proteins that act in endosome-to-Golgi retrieval. Our protocol employed siRNA transfection of cells stably expressing CD8-CIMPR and GFP-GOLPH3 in 96-well plates, followed by a three-step semiautomated anti-CD8 antibody uptake assay depicted in Figure 1A and detailed in Breusegem and Seaman (2014). The assay relies on a proportion of CD8-CIMPR being present at the cell surface at any given time. Thus, to assay endosome-to-Golgi retrieval the uptake of the CD8-CIMPR reporter by endocytosis is a necessary first step.

Images were acquired on an automated microscope, and, for every selected cell imaged, the retrieval of the anti-CD8 antibody to the TGN was quantified as a TGN retrieval ratio (Figure 1B). For each siRNA, TGN retrieval ratios were averaged over all measured cells and compared to average TGN retrieval ratios of negative and positive control wells.

Pilot Screen with Known Trafficking Genes

We first assessed our experimental workflow by assaying six mini-siRNA libraries, each containing between 41 and 60 ON TARGETplus siRNA pools that target human genes coding for known regulators of intracellular trafficking or cytoskeletal dynamics. In total, 310 individual genes were assayed in duplicate (data in Table S1), and this duplicate screen was repeated to assess biological reproducibility. Technical replicates showed high correlation coefficients for the TGN retrieval ratio (Figure 1C). Knockdown (KD) of most of the assayed genes yielded a TGN retrieval ratio that was similar to the value measured for the negative control wells (cyan data point, between 0.6 and 0.7), whereas KD of a few genes in each mini-library yielded a TGN retrieval ratio equal to or less than the one measured for the positive control cells (SNX1 KD, red data point, between 0.45 and 0.55). Biological replicates reproducibly generated a set of genes for which KD by siRNA led to a measured TGN retrieval ratio that was smaller than the one measured for SNX1 KD. This "hit" list included proteins with a well-documented role in endosome-to-Golgi retrieval and/or endocytosis (e.g., the retromer components VPS35 and SNX1, the GARP complex proteins VPS52 and VPS54, clathrin heavy chain, Golgi SNARE proteins STX5, STX10, GS15, and GS28). It also identified proteins that have not so far been linked to endosome-to-Golgi retrieval, e.g., PTPN23, ARRB1, and STX19.

To validate the role of these proteins in endosome-to-Golgi retrieval, the anti-CD8 uptake assay was repeated using the

Figure 1. Pilot Screen for Known Regulators of Endosome-to-Golgi Retrieval Using Anti-CD8 Uptake Assay

(A) Schematic of the anti-CD8 antibody retrieval assay as adapted for high-throughput screening. HeLa cells stably expressing a CD8-CIMPR reporter and the Golgi protein GFP-GOLPH3 were used. Anti-CD8 antibody (Ab) was bound at room temperature for 15 min and chased at 37°C for 30 min. Labeling and imaging details are in Supplemental Experimental Procedures.

(B) Definition of the TGN retrieval ratio used in our studies and depiction of subcellular antibody localizations that give rise to high or low TGN retrieval ratios.

(C) Scatterplots of the TGN retrieval ratios measured in replicate screens of 60 *VPS* gene homologs, 60 endocytosis genes, 43 SNARE protein genes, 41 dynein genes, 49 kinesin genes, and 43 myosin genes. Negative control measurements are in blue; SNX1 siRNA positive control measurements are in red. A linear fit to the data points is also shown. Marked data points indicate known endosome-to-Golgi retrieval pathway genes or genes that were selected for further validation.

(D) Replicate TGN retrieval ratios for the validation screen of the pilot study, in which 80 individual ON TARGETplus siRNA sequences (four per gene) were assayed. Controls and linear fit are as in (C).

(E) Representative images of the pilot validation screen for the negative control (no siRNA) and for five sets of siRNA-treated cells with reduced TGN retrieval ratio. Different phenotypes that give rise to reduced TGN retrieval ratios are discussed in the text. Scale bar, 100 μm (top two rows) and 38 μm (bottom row).

OPEN ACCESS
CellPress

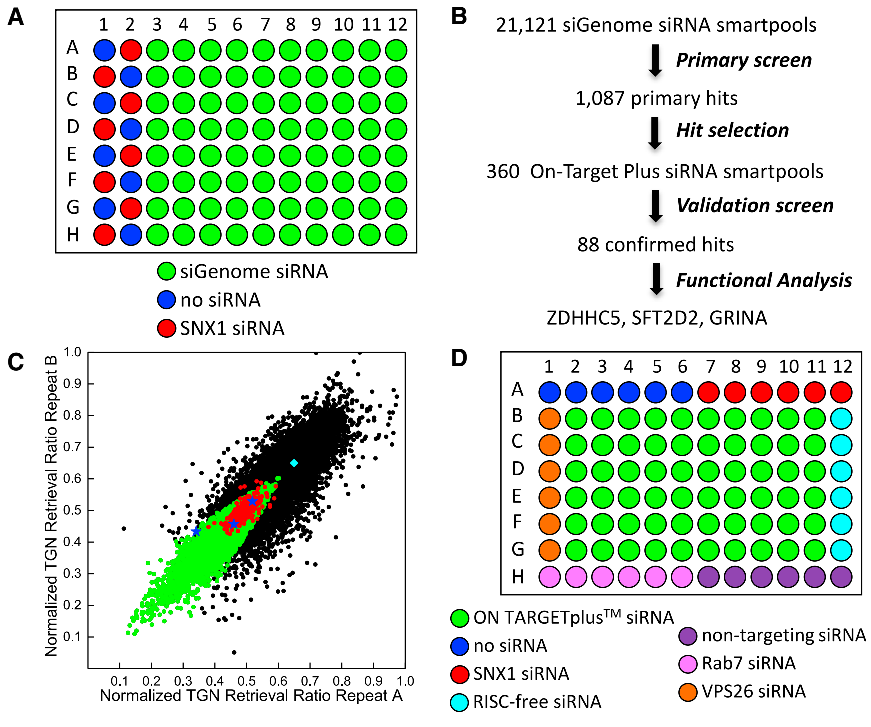

GRK6	MMGT1	TNS4	MNT
MAP3K6	MPP2	ACSF3	MVD
MAPK11	NDUFA3	AGBL4	NOV
MAPK12	NUP107	C1orf156	NPAT
PAK2	PLD3	C19orf45	OTX1
PKDCC	RYR1	CCL7	POC1B
PKN2	SEC11C	CIZ1	POLE
PPP4C	SFT2D2	CLTC	PRPF31
PTK2	SLC22A10	CWC15	PTBP1
STK3	SLC22A20	ECSIT	RAB1B
TNK2	SLC26A8	ERCC8	RBM14
C19orf26	SSR4	FAM24B	RNF123
C9orf167	TMEM201	GLRX	RPUSD4
CTXN1	TMTC4	GNL2	SELENBP1
DPAGT1	TXNDC15	GTF2E1	SFRS3
FZD3	ZDHHC5	H1FX	STRAP
GRINA	CEP192	KCTD13	TIMM44
KCNK3	CEP290	MAP1D	TRIM68
KCNV2	MCPH1	MBNL3	VAV3
LAPTM4A	SH3PXD2A	METTL8	VWC2L
LOC402377	SKA1	MFAP1	ZNF503
LY6E	SNCA	MIF	ZNF511

Figure 2. A Whole-Genome siRNA Screen to Identify Regulators of Endosome-to-Golgi Retrieval

(A) Plate layout for genome-wide loss-of-function screen.

(B) Whole-genome screen hit selection.

(C) Scatterplot of the normalized TGN retrieval ratios for the two replicates of the whole-genome screen. Each data point represents a single siRNA pool. Only valid measurements (18,465 siRNA pools) are included. Negative (cyan square) and positive (SNX1 siRNA, red data points) controls are shown. Green data points indicate siRNA pools with TGN retrieval ratio SSMD $\leq (-3)$ (very strong hits). Blue star data points indicate the three hits characterized further in Figures 3, 4, 5, and 6.

(D) Plate layout for the ON TARGETplus validation screen.

(E) Replicate normalized TGN retrieval ratios for the ON TARGETplus smartpool validation screen (360 pools). The colors used are as in (C), with additional magenta data points indicating siRNA pools with TGN retrieval ratio SSMD between (-2) and (-3) (strong hits).

(F) Table listing the 88 genes that were very strong or strong hits in the validation screen. Red-bordered cells indicate kinase and phosphatase genes, green-bordered cells contain genes encoding membrane proteins, and blue-bordered cells contain genes encoding cytoskeletal proteins. More information about these genes is provided in Table S4.

individual siRNA sequences of the ON TARGETplus pool in four separate siRNA transfections for 20 selected genes (Figure 1D). Ten out of the 20 genes were validated with two or more single siRNA oligos (Table S1). Using automated microscopy, both control cells and cells transfected with individual siRNA sequences (both of which express CD8-CIMPR and GFP-GOLPH3) were imaged after anti-CD8 antibody uptake. Example images in Figure 1E show that changes in anti-CD8 localization correlate with a decreased TGN retrieval ratio. For example, clathrin heavy chain (CLTC) KD results in a large fraction of anti-CD8 antibody binding to plasma membrane localized CD8-CIMPR, indicative of a defect in the endocytic uptake of the CD8-CIMPR reporter. In contrast, KD of DYNC1I2 causes an accumulation of anti-CD8 in cytoplasmic vesicles located at the cell periphery. Other examples in Figure 1E display intermediate phenotypes with reduced levels of anti-CD8 at the TGN and increased amounts of anti-CD8 in cytoplasmic vesicles.

Thus, the pilot screens provide a strong proof-of-principle that this strategy can be used to identify bona fide regulators of endosome-to-Golgi retrieval and candidate genes for further testing.

Genome-wide Primary Screen

We next carried out a genome-wide screen for effectors of endosome-to-Golgi retrieval of CIMPR using this same strategy with a human siRNA library. The genome-wide siRNA library contained 21,121 siGenome siRNA pools arrayed onto 267 library plates (Figure 2A). TGN retrieval ratios were normalized and strictly standardized mean difference (SSMD) values were calculated, both for the normalized TGN retrieval ratio and for the cellular anti-CD8 intensity, to allow hit selection based on a statistical analysis (see Experimental Procedures). Figure 2B summarizes the genome screen workflow, whereas Figure 2C shows a plot of the normalized TGN retrieval ratios measured in the two replicates of the genome-wide screen. Only valid data points are included, i.e., siRNAs that cause cell toxicity or severe cell division defects are not included. As in the pilot screen, data points are scattered around the diagonal indicating good reproducibility (correlation coefficient 0.88). Green data points represent 4,556 siRNAs with TGN retrieval ratio SSMD values smaller than (-3), indicating very strong siRNA effects. Positive control cells (SNX1 siRNA) also had a TGN retrieval ratio SSMD

≤ (−3) on the majority of the plates (red data points in Figure 2C). Further hit selection criteria included HeLa cell gene expression data and exclusion of hits that had been removed from the NCBI database (see Experimental Procedures for full details). This resulted in 1,087 hits from the genome-wide screen (~5% of the siRNAs screened) (Table S2).

Validation Screen

We selected 360 genes for further validation in a follow-up siRNA screen. For this, we arrayed ON TARGETplus siRNA pools in the 60 central wells of six 96-well plates (Figure 2D). The ON TARGETplus siRNA pools comprise, in many cases, completely distinct sequences from the ones in the siGenome siRNA pools and therefore are an effective means of validating the phenotype identified in the primary screen.

Similarly to the primary screen, we first assayed endosome-to-Golgi retrieval in HeLa cells stably expressing CD8-CIMPR and GFP-GOLPH3. The results are listed in Table S3, whereas Figure 2E shows the TGN retrieval ratios measured for the 360 genes in duplicate. Statistical analysis of the normalized TGN retrieval ratios yielded 44 very strong hits [12% of assayed genes with SSMD ≤ (−3), including clathrin, which is required for the uptake of the CD8-CIMPR reporter]. Because the ON TARGETplus siRNA pools are designed to higher specificity standards than the siGenome siRNA pools (Jackson et al., 2006), we also included 44 strong hits [−3 < SSMD ≤ (−2)] in our table of hits (Figure 2F). Interestingly, PLD3, a gene recently linked to late-onset AD (Cruchaga et al., 2014), was among the confirmed hits, whereas pathway analysis indicated a significant enrichment in genes linked to PD (SNCA, MAPK11, MAPK12). Further hits included genes with diverse predicted functions (kinases/phosphatases, red border in Figure 2F) and cellular locations (e.g., cytoskeleton, blue border in Figure 2F) as well as several membrane proteins (green border in Figure 2F). Available functional information, cellular location, and aliases of the 88 hit proteins are listed in Table S4.

Detailed Hit Characterization: SFT2D2, ZDHHC5, and GRINA

Closer inspection of the hits (Figure 2F) indicated a large number of multipass membrane-spanning proteins, including KCNK3, ZDHHC5, SLC22A10, SFT2D2, and GRINA. Many of these are high-confidence hits, i.e., the primary screen siGenome and validation screen ON TARGETplus siRNA pools for these genes did not share any sequences (Table S4). Current protein structure prediction tools suggest that ~26% of human genes encode membrane-spanning proteins, with almost half of these being multipass membrane proteins (Fagerberg et al., 2010; Kanapin et al., 2003). Yet, few multipass membrane-spanning proteins have been shown to affect membrane trafficking, and, to our knowledge, they have not been linked to endosome-to-Golgi retrieval in mammals. Therefore, we selected three multipass membrane-spanning high-confidence hits for further study: SFT2D2, ZDHHC5, and GRINA.

SFT2D2

SFT2D2 is partially homologous to the yeast Sft2p protein, a genetic interactor of Sed5p (the yeast syntaxin 5 protein) and affects post-Golgi trafficking (Conchon et al., 1999). However, the mammalian homologs (SFT2D1, SFT2D2, and SFT2D3) are uncharacterized. Images of anti-CD8 localization obtained in the primary screen show an accumulation of antibody in peripheral puncta in SFT2D2-silenced cells compared to control HeLa cells (Figure 3A) as well as reduced levels of antibody at the TGN.

To determine where in the endosome-to-Golgi pathway SFT2D2 could be acting, we established a cell line stably expressing Myc-tagged SFT2D2. We find that SFT2D2 localizes to both perinuclear membranes and structures positive for the retromer CSC protein, VPS35. The colocalization of SFT2D2 with VPS35 is especially apparent after treatment with nocodazole to depolymerize microtubules (Figure 3B). The reported genetic interaction between yeast Sft2p and Sed5 (Conchon et al., 1999) prompted us to evaluate the localization of SFT2D2 with respect to a number of SNARE proteins that function in post-Golgi trafficking. We find that SFT2D2 partially colocalizes with syntaxin 5 but exhibits almost complete colocalization with syntaxin 6 (indicated by arrowheads in the immunofluorescence images shown in Figure 3C). Quantitation of the colocalization of SFT2D2 with a number of post-Golgi SNARE proteins is shown graphically in Figure 3C (right panel) and confirms that SFT2D2 resides in a compartment strongly positive for syntaxin 6.

Extending the immunofluorescence-based investigation of SNARE protein distribution, when SFT2D2 expression is silenced by RNAi, we observed a marked change in the fluorescence intensity of several SNARE proteins, with syntaxin 6 and VAMP3 exhibiting the strongest change (Figure 3D). An example of the altered fluorescence intensity for VAMP3 is shown along with the graph in Figure 3D. Surprisingly, however, changes in the fluorescence intensity are not the result of changes in overall levels of the respective SNARE proteins (Figure 3E).

ZDHHC5

ZDHHC5 is a palmitoyl-acyl transferase enzyme that has been shown to palmitoylate a number of substrates, including the somatostatin receptor 5 (SSR5), flotillin-2, and GRIP1b (Kokkola et al., 2011; Li et al., 2012; Thomas et al., 2012). Its KD resulted in decreased amounts of anti-CD8 localization at the TGN and an increase in anti-CD8 positive puncta close to the plasma membrane following our antibody-uptake protocol (Figure 4A).

The ZDHHC5 protein, unlike most palmitoyl transferases, has an extensive C-terminal domain with four tyrosine-containing YxxΦ motifs that indicate ZDHHC5 undergoes clathrin-mediated sorting. Indeed, in cells stably expressing a Myc-tagged ZDHHC5 protein, we find that ZDHHC5 localizes extensively to the plasma membrane but also to intracellular tubular and vesicular structures (Figure 4B). As detailed in Figure S1, we further characterized the tubular structures on which ZDHHC5 is localized and found them to be Rab8a- or Rab11-positive recycling tubules. Figure 4B illustrates the partial colocalization of ZDHHC5 with the retromer CSC protein VPS35, which becomes more prominent after nocodazole treatment.

A reported substrate for ZDHHC5 is the SFT2D2 protein. Therefore, in cells expressing SFT2D2-Myc, ZDHHC5 expression was silenced using RNAi. Treated and control cells were mixed and seeded onto coverslips and then labeled with antibodies against ZDHHC5, Myc, and VPS35. In cells where ZDHHC5 expression was silenced (marked with *), the SFT2D2 staining appeared brighter and more concentrated in the perinuclear region (Figure 4C).

OPEN
ACCESS
CellPress

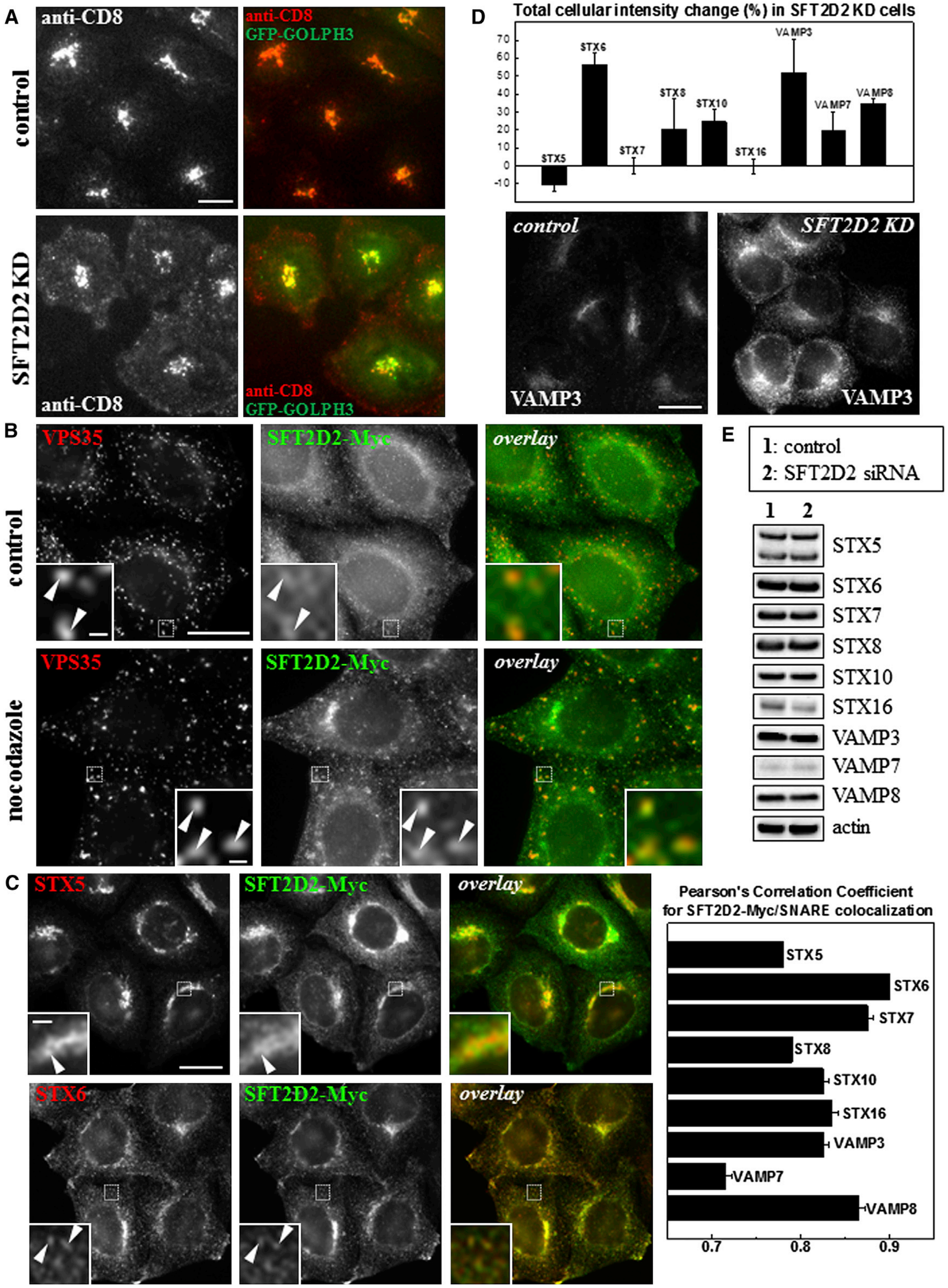

Figure 3. Characterization of SFT2D2 Function in Endosome-to-Golgi Retrieval

(A) Primary screen anti-CD8 antibody-uptake images for control cells (top) and SFT2D2 KD cells (bottom).

(B) Cells stably expressing SFT2D2-Myc show localization of SFT2D2 to perinuclear membranes and endosomes, including VPS35-positive endosomes (arrowheads in inset). Treatment with nocodazole disperses the endosomes and even more clearly shows colocalization of SFT2D2-Myc and VPS35 (arrowheads in inset).

(legend continued on next page)

The retromer and the WASH complexes can mediate endosome-to-plasma membrane recycling in addition to endosome-to-Golgi retrieval (Derivery et al., 2009; Gomez and Billadeau, 2009; Steinberg et al., 2013; Temkin et al., 2011). Therefore, we examined the effect of ZDHHC5 KD on the localization of α5β1-integrin, a cargo protein of retromer in the endosome-to-plasma membrane pathway (Duleh and Welch, 2012; Zech et al., 2011). Both plasma membrane localized and intracellular stores of α5-integrin (Figure 4D), as well as β1-integrin (Figure 4E), are markedly increased in ZDHHC5-silenced cells (marked by an asterisk in Figure 4D) compared to control cells.

In separate experiments, we assessed and quantified the localization and expression of several post-Golgi SNARE proteins upon ZDHHC5 KD (Figure S2). Although immunofluorescence indicated increased staining of several (STX6, STX7, STX8) but not all (e.g., STX16) SNARE proteins, total cellular SNARE protein levels as assessed by western blotting were unchanged upon ZDHHC5 KD (Figure S2).

GRINA

GRINA (*g*lutamate *r*eceptor, *i*onotropic, *N*-methyl D-aspartate-*a*ssociated protein 1), also known as LFG1 or TMBIM3, is a 42 kDa protein with a conserved BAX inhibitor-1 motif and a reported role in protecting cells from ER stress-induced apoptosis (Rojas-Rivera et al., 2012). In addition, a C-terminal fragment of the protein protects HeLa cells from Shiga-toxin induced cytotoxicity (Yamaji et al., 2010). Images from the primary screen indicated that KD of GRINA in HeLa cells expressing CD8-CIMPR and GFP-GOLPH3 leads to a reduction in anti-CD8 antibody that reaches the TGN after a 30 min chase period (Figure 5A). In addition, we noted that our chosen TGN marker, GOLPH3, appeared slightly fragmented in the GRINA KD cells. Further characterization of cells in which GRINA expression was suppressed using siRNA showed reduced levels of TGN46 at the TGN (Figure 5C) and in total cell lysates (Figure 5D), whereas GFP-Rab6 levels and appearance were unaltered (Figures 5C and 5D).

GRINA KD severely altered expression of GLG1, a type-I Golgi membrane protein that traffics from endosomes to the Golgi (Ahn et al., 2005) (Figure 5B and leftmost western blot in Figure 5D), but had no effect on *cis*-Golgi localized GM130 levels (Figure 5D, rightmost western blot). The effect of the loss of GRINA on other Golgi localized proteins (e.g., α-mannosidase II and STX5) or endo/lysosomal proteins (e.g., EEA1 and LAMP1) is shown in Figure S3. In some instances, fragmentation of the *cis*-Golgi was observed in cells in which GRINA was silenced (see Figure 5B), but no changes were observed in the distribution or intensity of the core retromer protein VPS35 (Figures 5C and S3A).

Transient transfection of HeLa cells with Myc-tagged GRINA had pronounced effects on both TGN and endosome morphology. In particular, transfected cells showed a shrunken TGN with reduced levels of TGN46, whereas endosomes appeared enlarged (Figures 5E and 5F). Myc-tagged GRINA colocalized with the remaining TGN46 and also extensively with VPS35-positive (Figure 5F) and SNX1-positive (Figure S3D) endosomes, including endosomes that appeared enlarged. GRINA overexpression severely disrupted CIMPR staining, with CIMPR found in GRINA-positive vesicular structures, some enlarged, scattered throughout the cell (Figure 5G).

SFT2D2, ZDHHC5, and GRINA Loss Does Not Disrupt Retromer Complex but Leads to Altered Levels of Endosome-to-Golgi Cargo Proteins

To address how SFT2D2, ZDHHC5, or GRINA might affect endosome-to-Golgi retrieval and CIMR trafficking, we performed a biochemical analysis of how their loss affects the integrity of the retromer complex or its interaction with the WASH complex. Native coimmunoprecipitation experiments determined that KD of SFT2D2, ZDHHC5, or GRINA did not affect the protein interactions between the cargo-selective retromer components VPS26, VPS29, and VPS35, nor the interaction between these core retromer components and WASH complex members (strumpellin, FAM21) or TBC1D5, suggesting that SFT2D2, ZDHHC5, and GRINA do not act directly on the cargo-selective retromer complex or the WASH complex (Figure S4A).

We next assessed whether silencing of SFT2D2, ZDHHC5, or GRINA affected levels of retromer cargo proteins. In addition to blotting for various membrane proteins in crude lysates, immobilized wheat germ agglutinin (WGA), a lectin that binds to galactose and sialic acid moieties, was used to enrich for Golgi and post-Golgi localized glycosylated membrane proteins from lysates of control or KD cells where the retromer components VPS26 or SNX1, or SFT2D2, ZDHHC5, or GRINA, were silenced (Figure 6A). Silencing of ZDHHC5 or SFT2D2, respectively, resulted in reduced levels of CIMPR or TGN46 bound to the lectin, whereas silencing GRINA reduced lectin-bound levels of GLG1, CIMPR, and TGN46 but also decreased their abundance in the lysates from cycloheximide-treated cells (Figure 6A; Figure S4B). Importantly, levels of the transferrin receptor (TfnR) that cycles from endosomes to the cell surface were not affected in any of the KDs. Interestingly, levels of SFT2D2 were found to be strongly affected by the KD of SNX1, suggesting that its steady-state localization may be regulated by retromer.

Using automated microscopy, we next examined the fluorescence intensity of two endogenous proteins known to traffic from endosomes to the TGN in a retromer-dependent manner: the CIMPR and TGN46 (Figure 6B). We find that RNAi-mediated KD of SFT2D2, ZDHHC5, or GRINA all lead to an increase in

(C) Cells stably expressing SFT2D2-Myc were costained with antibodies against various post-Golgi SNARE proteins. Colocalization was quantified by Pearson's correlation coefficient (right-hand graph) and indicates very extensive colocalization with STX6, STX7, and VAMP8. The image panels illustrate colocalization of SFT2D2-Myc and STX5 at the Golgi (top, arrowhead) but much more extensive colocalization of SFT2D2-Myc and STX6 (bottom, arrowheads). In (B) and (C), the white dashed box delineates the area magnified in the insets.

(D) SNARE protein staining was compared for control and SFT2D2 KD HeLa cells and quantified. The graph shows the change in cellular intensity measured for each post-Golgi SNARE investigated. Images illustrate the increased cellular intensity of VAMP3 in SFT2D2 KD cells.

(E) Control and SFT2D2 KD HeLa cell lysates were separated by LDS-PAGE and blotted for the indicated SNARE proteins or actin. Scale bars in (A)–(D), 20 μm, except insets in (B), 1 μm, and in (C), 2 μm. Quantitation in (C) and (D) was done using automated microscopy (see Experimental Procedures); error bars indicate SD of two separate multicell experiments. Average Pearson's correlation coefficients measured were often identical in repeated experiments.

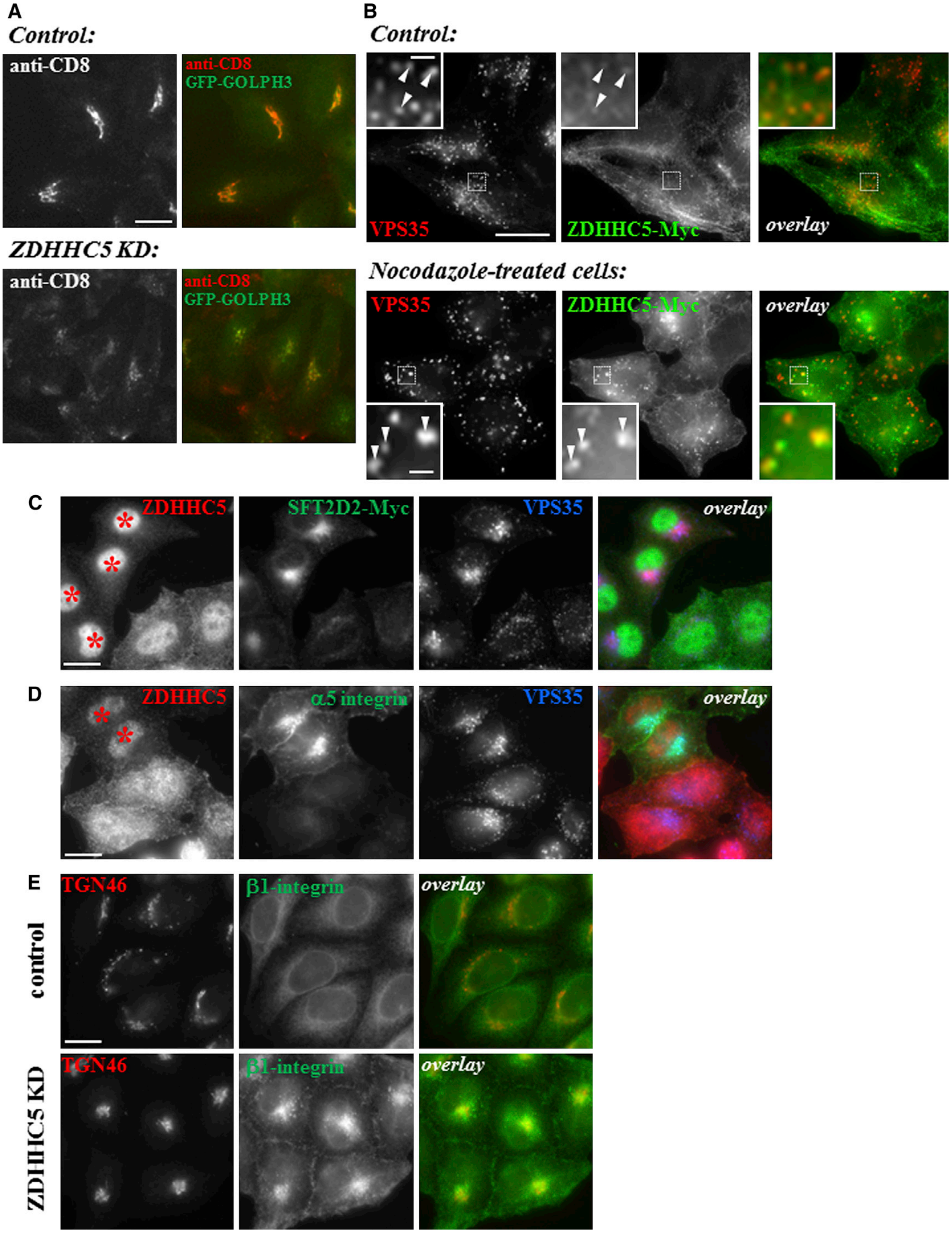

Figure 4. Localization and Characterization of ZDHHC5 in HeLa Cells

(A) Primary screen anti-CD8 antibody-uptake images for control cells (top) and ZDHHC5 KD cells (bottom).

(B) Cells stably expressing ZDHHC5-Myc show localization of ZDHHC5 to the plasma membrane and to intracellular tubules and vesicles. Some colocalization between ZDHHC5 and retromer VPS35 is observed (arrowheads in inset). Following nocodazole treatment endosomes are dispersed and some are labeled with ZDHHC5 and VPS35 (arrowheads in inset).

(C) Control and ZDHHC5 siRNA-treated SFT2D2-Myc cells were mixed and stained for ZDHHC5, Myc, and VPS35. KD cells are marked by an asterisk.

(D) Control and ZDHHC5 siRNA-treated HeLa cells were mixed and stained for ZDHHC5, α5-integrin, and VPS35. KD cells are indicated with an asterisk.

(E) Control (top) and ZDHHC5 KD (bottom) HeLa cells were fixed and stained for TGN46 and β1-integrin.

Scale bars in (A)–(E), 20 μm, except inset in (B), 2 μm.

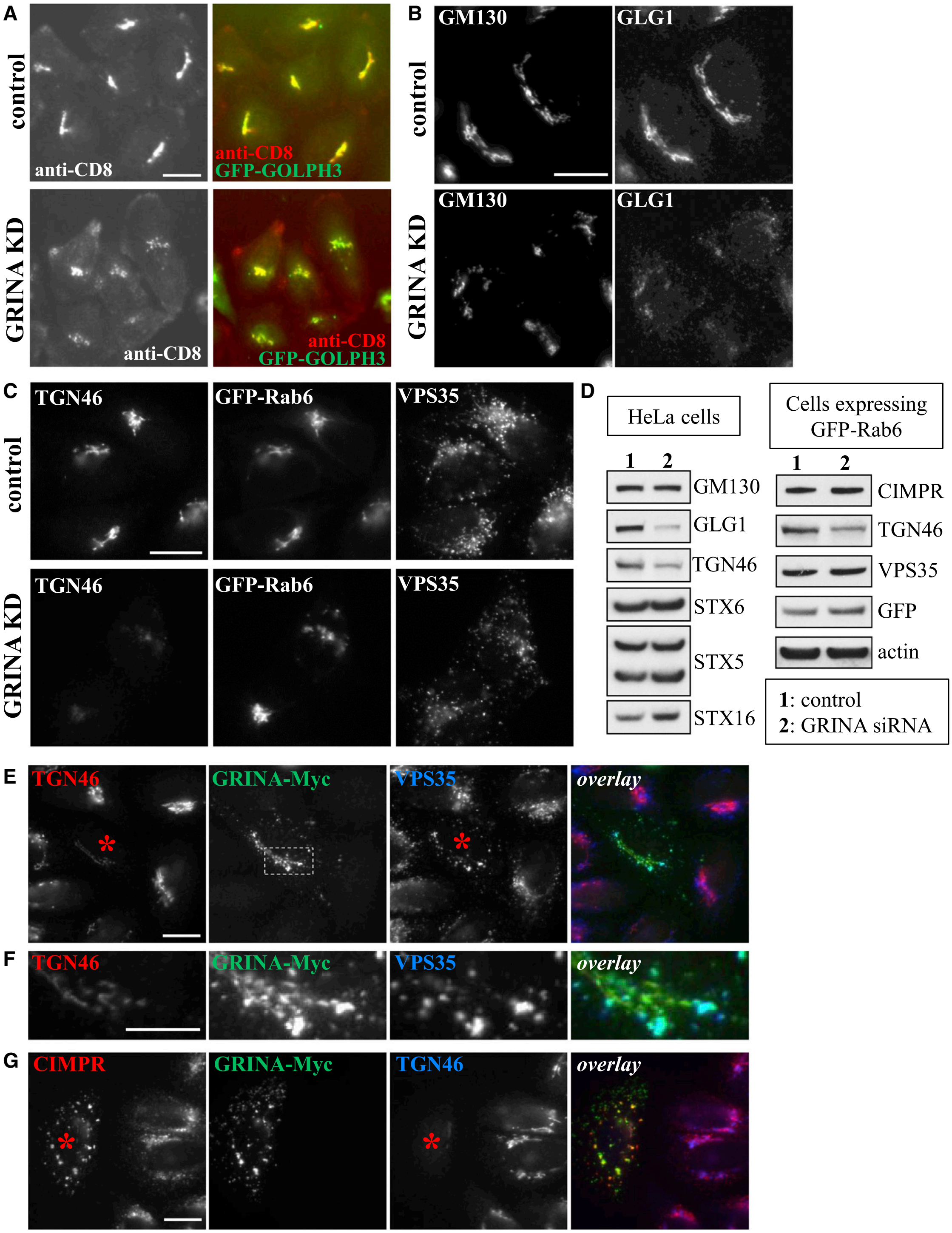

Figure 5. GRINA Knockdown or Overexpression Inhibits Endosome-to-Golgi Retrieval

(A) Primary screen anti-CD8 antibody uptake images for control cells (top) and GRINA KD cells (bottom).

(B) Control (top) and GRINA KD (bottom) HeLa cells were stained for Golgi marker GM130 and Golgi glycoprotein-1 (GLG1).

(C) Cells stably expressing GFP-Rab6 were treated with GRINA siRNA (bottom) and compared to control cells (top) upon staining for TGN46 and VPS35.

(D) Quantitation by western blotting of lysates from control and GRINA-silenced HeLa cells and GFP-Rab6 cells.

(E–G) HeLa cells were transiently transfected with GRINA-Myc for 24 hr before fixing and staining. (E and F) GRINA-Myc colocalizes with both TGN46 and VPS35. In this example, GRINA-Myc expression (in the cell marked by an asterisk in E) reduced the cell's TGN46 expression compared to surrounding untransfected cells and caused enlargement of VPS35-positive endosomes. The area in (E) magnified in (F) is indicated by a dashed line. (G) GRINA-Myc

(legend continued on next page)

the CIMPR fluorescence intensity although GRINA KD markedly reduces TGN46 staining. Quantitative analysis shows increased CIMPR fluorescence intensity in Golgi structures marked by mannosidase II or GM130 (Figure 6C) and also, for KD of SFT2D2 and ZDHHC5, increased correlation coefficients for CIMPR and VPS35 (Figure 6D). VPS35 endosomes are also significantly brighter in the three types of KD cells compared to control cells (Figure S4C). The RNAi-mediated silencing of GRINA appears to result in some loss of Golgi integrity and results in a general reduction in fluorescence intensity for both TGN46 and GLG1 (Figure 6E).

Collectively, these experiments indicate that SFT2D2 and ZDHHC5 are important for recycling of specific cargo proteins to the Golgi, whereas loss of GRINA may have more wide-ranging effects on TGN structure and stability of a number of membrane proteins.

DISCUSSION

Endosome-to-Golgi retrieval is an essential pathway in several key physiological processes and is exploited by bacterial and viral pathogens, underscoring the importance of expanding our understanding of the pathway. Here, we have conducted a genome-wide RNAi screen using an antibody-uptake assay and a model cargo reporter to identify ~90 proteins that, when silenced, impede the endosome-to-Golgi retrieval. This included a number of kinases, phosphatases, and several multipass membrane spanning proteins that were uncharacterized with respect to endosome-to-Golgi retrieval.

We successfully adapted our antibody-uptake assay for this high-throughput screen. A pilot screen of siRNA mini-libraries that targeted known membrane trafficking genes demonstrated the feasibility of using the CD8 antibody-uptake assay in 96-well plates, identifying known endosome-to-Golgi components such as retromer VPS35 and the GARP complex proteins VPS52 and VPS54 (Pérez-Victoria et al., 2008) as hits. In addition, the pilot screen identified proteins with a role in endosome-to-Golgi retrieval that can now be further investigated, e.g., PTPN23 (also known as HD-PTP), KIFC1 (also known as HSET), and STX19.

Our genome-wide screen generated ~1,100 hits, including known endosome-to-Golgi participants, such as VPS26, SNX6, VPS54, and endocytic proteins such as clathrin. Other known components of the endosome-to-Golgi retrieval pathway fell just below our stringent hit selection cutoff (e.g., VPS29, SNX5, Rab7a, VPS52). Our distribution of hits is similar to other reported screens, e.g., the screen that reported a role for retromer in HPV infectivity (Lipovsky et al., 2013). The presence of genes known to mediate endocytosis (e.g., clathrin heavy chain) among the hits is a predictable consequence of the use of the antibody-uptake assay. The accumulation of anti-CD8 at the cell surface observed in a clathrin KD (see Figure 1E) provides a useful benchmark against which to compare the other hits reported in our study, and, among the ~90 endosome-to-Golgi genes we report, none produce a phenotype like the clathrin KD.

We validated our hits from the primary screen using ON TARGETplus siRNA pools. Not only are the four siRNA duplexes in these second generation siRNA pools chemically modified to avoid off-target effects, their sequences are also, for a great many genes, entirely different from the ones in our whole-genome library (siGenome siRNA). They are therefore an effective means of validating our primary screen hits.

Among the confirmed high-confidence hits, we noted several kinases, phosphatases, and proteins that have been implicated in PD. As the retromer protein, VPS35, has been revealed to be a PD gene (Vilariño-Güell et al., 2011; Zimprich et al., 2011), the identification of other PD genes among the hits is consistent with the important role that endosomal protein sorting plays in the pathology of PD.

There was also an enrichment for multipass membrane spanning proteins, a group often overlooked with respect to membrane trafficking regulation. In fact, very few multipass membrane-spanning proteins have a reported role in membrane trafficking. Examples to date include the function of Atg9 in autophagy (reviewed in Reggiori and Tooze, 2012) and that of CLN3 in anterograde post-Golgi trafficking (Metcalf et al., 2008). Thus, this screen has revealed a role for this class of proteins in endosome-to-Golgi retrieval.

We chose three multipass membrane spanning proteins for further characterization: SFT2D2, ZDHHC5, and GRINA. Although each of them, when silenced, had a strong effect on endosome-to-Golgi retrieval of CD8-CIMPR and also affected other cargoes of the endosome-to-Golgi pathway such as TGN46 and GLG1, none affected retromer integrity or compromised retromer association with proteins such as the WASH complex or TBC1D5. Additionally, no association between retromer and the SFT2D2, ZDHHC5, or GRINA proteins has been detected thus far (S.Y.B. and M.N.J.S., unpublished data).

SFT2D2 is evolutionarily conserved, and yeast Sft2p, first described as a genetic interactor of Sed5p (the fungal syntaxin 5 protein; Banfield et al., 1995), has been implicated in post-Golgi membrane traffic (Conchon et al., 1999). We report that SFT2D2 localizes to structures positive for TGN and endosomal markers. ZDHHC5 is a palmitoyl transferase reported to localize to the plasma membrane (Ohno et al., 2006). The extended cytoplasmic tail of ZDHHC5 contains several YxxΦ motifs required for clathrin-mediated sorting processes and marks out ZDHHC5 as distinct from most other palmitoyl transferases (Korycka et al., 2012). We show localization of ZDHHC5-Myc to retromer-positive endosomes and endosomal recycling tubules positive for EHD1, Rab8, and Rab11. EHD1 operates with retromer in endosome-to-Golgi retrieval (Gokool et al., 2007; Zhang et al., 2012), and both EHD1 and Rab11-mediated endosomal protein recycling have recently been shown to regulate localization and processing of APP consistent with the prominent role that endosomal protein sorting plays in AD (Buggia-Prévot et al., 2013;

expression also perturbs CIMPR localization. In this example, GRINA-Myc transfection (in the cell marked by an asterisk) caused CIMPR to localize to round vesicular structures positive for GRINA-Myc, some of which appeared larger than regular endosomes, whereas TGN46 staining was almost absent in the transfected cell.
Scale bars in (A)–(C), (E), and (G), 20 μm, and in (F), 10 μm.

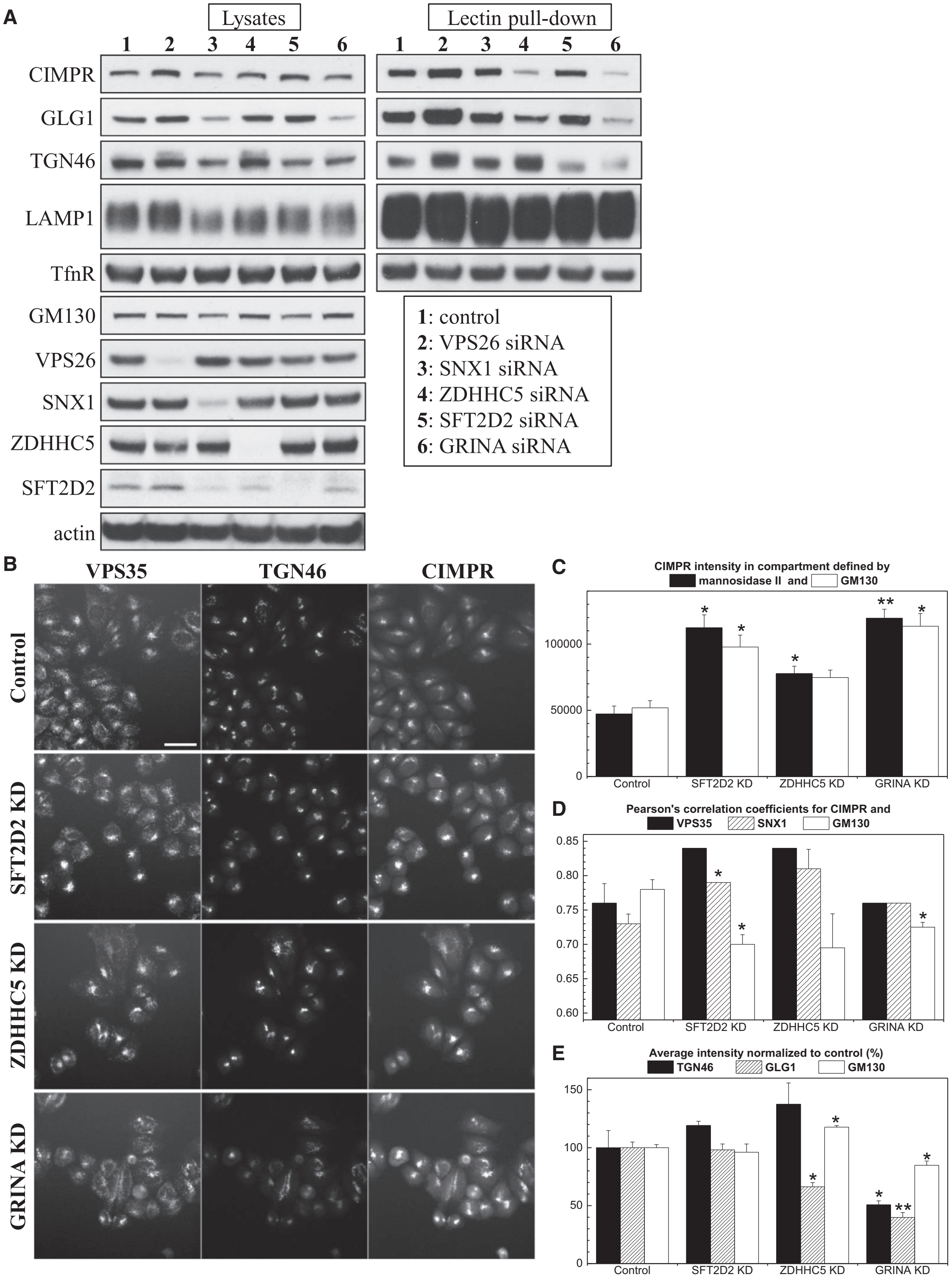

Figure 6. ZDHHC5, SFT2D2, or GRINA Depletion Affect Levels of Endosome-to-Golgi Cargo Proteins

(A) Control HeLa cells or cells transfected with the indicated siRNAs were treated for 3 hr with cycloheximide, lysed, and incubated with agarose-bound wheat germ agglutinin to capture glycosylated membrane proteins. Total cell lysates (left) and lectin pull-down samples (right) were assayed by western blotting. The experiment was repeated three times, and representative data are shown.

(B–E) Quantitative analysis of SFT2D2, ZDHHC5, and GRINA KD cells immunofluorescence using automated microscopy (see Experimental Procedures). (B) Representative images showing VPS35, TGN46, and CIMPR staining. Scale bar, 50 μm. (C) Quantitative analysis of CIMPR intensity at the Golgi indicates a

(legend continued on next page)

OPEN
ACCESS
CellPress

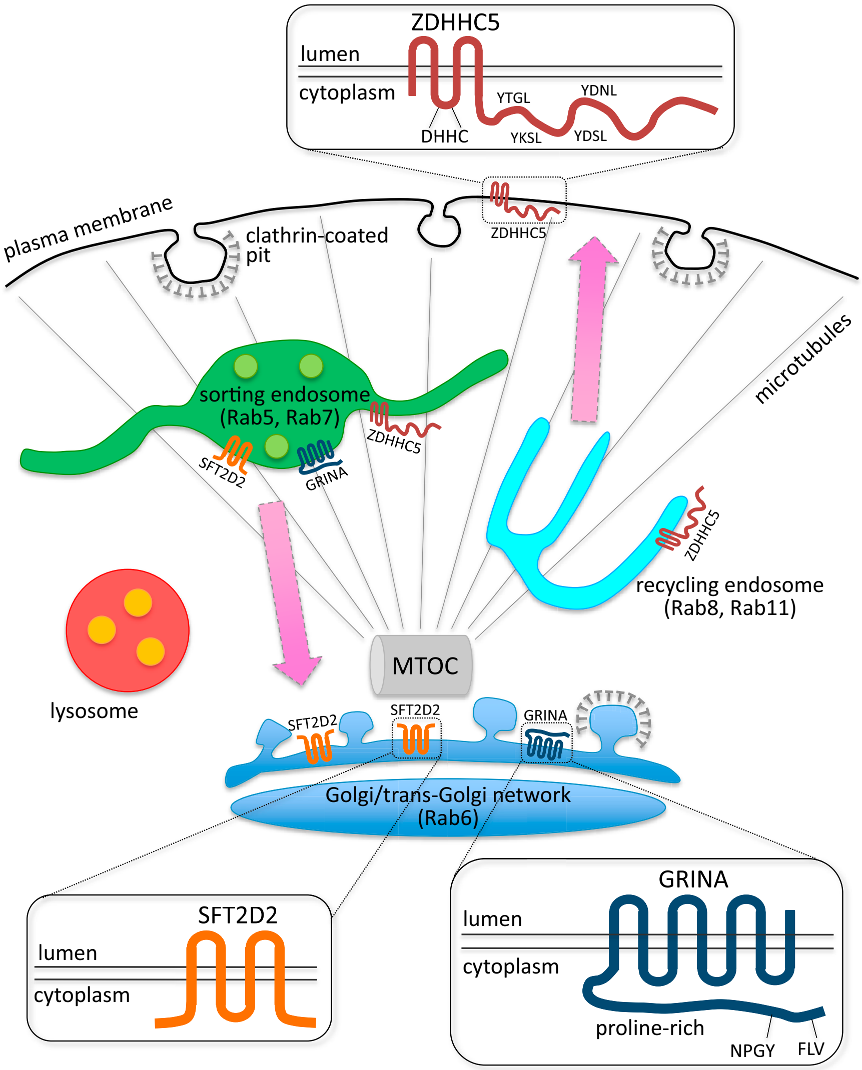

Figure 7. Model

Schematic depiction of the localization and topology of the three multipass membrane proteins identified as regulators of endosome-to-Golgi retrieval. Features such as the YxxΦ motifs in ZDHHC5 are indicated.

Udayar et al., 2013). Palmitoylation of the cytoplasmic tail of the CIMPR does facilitate its endosome-to-Golgi retrieval, but this modification is mediated by ZDHHC15, a palmitoyl transferase distinct from ZDHHC5 (McCormick et al., 2008). Interestingly, SFT2D2 has been identified as a substrate of ZDHHC5 (Li et al., 2012), and we indeed find that ZDHHC5 KD affects the localization of SFT2D2.

The KD of either SFT2D2 or ZDHHC5 resulted in changes to the fluorescence intensity of several SNARE proteins. For example, loss of SFT2D2 markedly increased the fluorescence intensity of syntaxin 6 and VAMP3. These two SNAREs have been shown to function together (Riggs et al., 2012), so it is perhaps not surprising that both are affected by SFT2D2 KD. Although the fluorescence intensity of several SNAREs was affected by SFT2D2 or ZDHHC5 KD, western blotting indicated that overall levels of the SNARE proteins were not altered. The increased fluorescence must therefore result from either altered localization of the respective SNARE, so it is more concentrated in a discrete localization, and hence the fluorescence intensity is increased, or from changes in the conformation of the respective SNARE such that the antisera used for the immunofluorescence staining has greater access to its epitope resulting in increased fluorescence intensity. Further work is required to distinguish these two possibilities.

GRINA is a member of the LFG (Lifeguard) family of proteins, a family of proteins highly conserved among eukaryotes with a proposed protective role in apoptosis (Hu et al., 2009), a function also reported for GRINA (Rojas-Rivera et al., 2012). We observed that GRINA-Myc localizes to the Golgi and to post-Golgi membranes, and that its expression has a dominant-negative effect on the appearance of both the Golgi and endosomes. These observations are in line with a report that GRINA induces resistance to Shiga toxin (Yamaji et al., 2010). In addition, we found that GRINA KD disrupted Golgi localization of several but not all Golgi-localized proteins and reduced the steady-state levels of a number of endosome-to-Golgi cargo proteins including TGN46. Our analyses of the three multipass membrane-spanning proteins, summarized in Figure 7, confirm their function in endosome-to-Golgi retrieval, and future studies may reveal precisely how these proteins operate in this pathway.

Other genes identified as modulating endosome-to-Golgi and reported herein are good candidates for further studies focused on the pathology of AD and PD. A recent analysis of genes encoding retromer-associated proteins identified single nucleotide polymorphisms and other variants linked with late-onset AD (Vardarajan et al., 2012). We report here that PLD3, a gene that increases risk of AD (Cruchaga et al., 2014), is required for

significant increase in SFT2D2, ZDHHC5, or GRINA KD cells. (D) SFT2D2, ZDHHC5, and GRINA KD increase the Pearson's correlation coefficient for colocalization between CIMPR and retromer proteins VPS35 or SNX1 while decreasing the correlation between CIMPR and Golgi matrix protein GM130. In some cases, identical correlation coefficients were measured in the replicate experiments. (E) Quantitation of the TGN46 and GLG1 intensity in the three types of KD cells. In (C)–(E), error bars indicate the SD of the replicate measurements. *$p < 0.05$; **$p < 0.01$.

endosome-to-Golgi retrieval and therefore establish a role for PLD3 in a pathway that is now viewed as key to events early in the pathology of AD (Small, 2008; Willnow and Andersen, 2013). Thus, other genes we report merit investigation for linkage to diseases such as AD and PD and are also worth examining for a role in the processes that underlie infection by bacterial and viral pathogens (e.g., Legionella and HPV) that exploit the endosome-to-Golgi pathway for their own ends.

EXPERIMENTAL PROCEDURES

Screening

Using a Beckman Biomek robot, cells stably expressing CD8-CIMPR and GFP-GOLPH3 (described in Harbour et al., 2010) were seeded onto 20 μl 180 nM siGenome (for the primary screen) or ON TARGETplus (for the pilot screen and validation screens) siRNA pools in 96-well plates. After 72 hr at 37°C, cells were allowed to bind anti-CD8 antibody at room temperature for 15 min, washed, and then chased for 30 min at 37°C before fixing and immunolabeling. Images were acquired on a Cellomics Arrayscan V automated microscope and analyzed using the Cellomics vHCS:View software and its colocalization bioapplication. Data were exported for further analysis, including plate-wise normalization and SSMD calculation, in SQL and Origin.

Characterization of SFT2D2, ZDHHC5, and GRINA

Myc-tagged human SFT2D2, ZDHHC5, and GRINA constructs were purchased from Origene and used for transient transfections in HeLa cells including into cells stably expressing GFP- or mStrawberry-tagged Rab or EHD1 constructs. Immunolabeled cells were imaged on a wide-field fluorescence microscope (Zeiss). siRNA-mediated silencing was performed using ON TARGETplus siRNA pools. Retromer assembly and interactions were investigated by immunoprecipitation using cells stably expressing VPS29-GFP and published protocols (Harbour et al., 2012). Cargo protein glycosylation changes upon protein silencing were assessed using agarose-bound wheat germ agglutinin (Sigma) precipitation followed by western blotting.

Supplemental Experimental Procedures, including screening details, hit selection criteria, and antibodies used are in Supplemental Information. Screening data not available in Tables S1, S2, S3, and S4, as well as images from any of the reported screens, are available upon request (syab2@cam.ac.uk).

SUPPLEMENTAL INFORMATION

Supplemental Information includes Supplemental Experimental Procedures, four figures, and four tables and can be found with this article online at http://dx.doi.org/10.1016/j.celrep.2014.10.053.

ACKNOWLEDGMENTS

This work was financially supported through a Medical Research Council Senior Research Fellowship awarded to M.N.J.S. Work in the CIMR is also supported by a Welcome Trust Strategic Award (100140). The authors wish to thank Dr. Andrew Peden for antibodies and the SNARE siRNA mini-library, Dr. Folma Buss and Prof. Scottie Robinson for siRNA mini-libraries, and Patrycja Kozik and Nicole Simecek for advice on and help with RNAi screening and data analysis. We additionally thank Scottie Robinson and Alison Schuldt for helpful comments on the manuscript.

Received: June 6, 2014
Revised: September 18, 2014
Accepted: October 17, 2014
Published: November 20, 2014

REFERENCES

Ahn, J., Febbraio, M., and Silverstein, R.L. (2005). A novel isoform of human Golgi complex-localized glycoprotein-1 (also known as E-selectin ligand-1, MG-160 and cysteine-rich fibroblast growth factor receptor) targets differential subcellular localization. J. Cell Sci. *118*, 1725–1731.

Arighi, C.N., Hartnell, L.M., Aguilar, R.C., Haft, C.R., and Bonifacino, J.S. (2004). Role of the mammalian retromer in sorting of the cation-independent mannose 6-phosphate receptor. J. Cell Biol. *165*, 123–133.

Banfield, D.K., Lewis, M.J., and Pelham, H.R. (1995). A SNARE-like protein required for traffic through the Golgi complex. Nature *375*, 806–809.

Belenkaya, T.Y., Wu, Y., Tang, X., Zhou, B., Cheng, L., Sharma, Y.V., Yan, D., Selva, E.M., and Lin, X. (2008). The retromer complex influences Wnt secretion by recycling wntless from endosomes to the trans-Golgi network. Dev. Cell *14*, 120–131.

Bonifacino, J.S., and Hurley, J.H. (2008). Retromer. Curr. Opin. Cell Biol. *20*, 427–436.

Breusegem, S.Y., and Seaman, M.N. (2014). Image-based and biochemical assays to investigate endosomal protein sorting. Methods Enzymol. *534*, 155–178.

Buggia-Prévot, V., Fernandez, C.G., Udayar, V., Vetrivel, K.S., Elie, A., Roseman, J., Sasse, V.A., Lefkow, M., Meckler, X., Bhattacharyya, S., et al. (2013). A function for EHD family proteins in unidirectional retrograde dendritic transport of BACE1 and Alzheimer's disease Aβ production. Cell Reports *5*, 1552–1563.

Carlton, J., Bujny, M., Peter, B.J., Oorschot, V.M., Rutherford, A., Mellor, H., Klumperman, J., McMahon, H.T., and Cullen, P.J. (2004). Sorting nexin-1 mediates tubular endosome-to-TGN transport through coincidence sensing of high- curvature membranes and 3-phosphoinositides. Curr. Biol. *14*, 1791–1800.

Conchon, S., Cao, X., Barlowe, C., and Pelham, H.R. (1999). Got1p and Sft2p: membrane proteins involved in traffic to the Golgi complex. EMBO J. *18*, 3934–3946.

Cruchaga, C., Karch, C.M., Jin, S.C., Benitez, B.A., Cai, Y., Guerreiro, R., Harari, O., Norton, J., Budde, J., Bertelsen, S., et al.; UK Brain Expression Consortium; Alzheimer's Research UK Consortium (2014). Rare coding variants in the phospholipase D3 gene confer risk for Alzheimer's disease. Nature *505*, 550–554.

Derivery, E., Sousa, C., Gautier, J.J., Lombard, B., Loew, D., and Gautreau, A. (2009). The Arp2/3 activator WASH controls the fission of endosomes through a large multiprotein complex. Dev. Cell *17*, 712–723.

Duleh, S.N., and Welch, M.D. (2012). Regulation of integrin trafficking, cell adhesion, and cell migration by WASH and the Arp2/3 complex. Cytoskeleton *69*, 1047–1058.

Fagerberg, L., Jonasson, K., von Heijne, G., Uhlén, M., and Berglund, L. (2010). Prediction of the human membrane proteome. Proteomics *10*, 1141–1149.

Fjorback, A.W., and Andersen, O.M. (2012). SorLA is a molecular link for retromer-dependent sorting of the Amyloid precursor protein. Commun. Integr. Biol. *5*, 616–619.

Fjorback, A.W., Seaman, M., Gustafsen, C., Mehmedbasic, A., Gokool, S., Wu, C., Militz, D., Schmidt, V., Madsen, P., Nyengaard, J.R., et al. (2012). Retromer binds the FANSHY sorting motif in SorLA to regulate amyloid precursor protein sorting and processing. J. Neurosci. *32*, 1467–1480.

Ganley, I.G., Espinosa, E., and Pfeffer, S.R. (2008). A syntaxin 10-SNARE complex distinguishes two distinct transport routes from endosomes to the trans-Golgi in human cells. J. Cell Biol. *180*, 159–172.

Gokool, S., Tattersall, D., and Seaman, M.N. (2007). EHD1 interacts with retromer to stabilize SNX1 tubules and facilitate endosome-to-Golgi retrieval. Traffic *8*, 1873–1886.

Gomez, T.S., and Billadeau, D.D. (2009). A FAM21-containing WASH complex regulates retromer-dependent sorting. Dev. Cell *17*, 699–711.

Harasaki, K., Lubben, N.B., Harbour, M., Taylor, M.J., and Robinson, M.S. (2005). Sorting of major cargo glycoproteins into clathrin-coated vesicles. Traffic *6*, 1014–1026.

Harbour, M.E., Breusegem, S.Y., Antrobus, R., Freeman, C., Reid, E., and Seaman, M.N. (2010). The cargo-selective retromer complex is a recruiting hub for

OPEN
ACCESS
CellPress

protein complexes that regulate endosomal tubule dynamics. J. Cell Sci. *123*, 3703–3717.

Harbour, M.E., Breusegem, S.Y., and Seaman, M.N. (2012). Recruitment of the endosomal WASH complex is mediated by the extended 'tail' of Fam21 binding to the retromer protein Vps35. Biochem. J. *442*, 209–220.

Hong, Z., Yang, Y., Zhang, C., Niu, Y., Li, K., Zhao, X., and Liu, J.J. (2009). The retromer component SNX6 interacts with dynactin p150(Glued) and mediates endosome-to-TGN transport. Cell Res. *19*, 1334–1349.

Hu, L., Smith, T.F., and Goldberger, G. (2009). LFG: a candidate apoptosis regulatory gene family. Apoptosis *14*, 1255–1265.

Jackson, A.L., Burchard, J., Leake, D., Reynolds, A., Schelter, J., Guo, J., Johnson, J.M., Lim, L., Karpilow, J., Nichols, K., et al. (2006). Position-specific chemical modification of siRNAs reduces "off-target" transcript silencing. RNA *12*, 1197–1205.

Kanapin, A., Batalov, S., Davis, M.J., Gough, J., Grimmond, S., Kawaji, H., Magrane, M., Matsuda, H., Schönbach, C., Teasdale, R.D., and Yuan, Z.RIKEN GER Group; GSL Members (2003). Mouse proteome analysis. Genome Res. *13* (6B), 1335–1344.

Kokkola, T., Kruse, C., Roy-Pogodzik, E.M., Pekkinen, J., Bauch, C., Hönck, H.H., Hennemann, H., and Kreienkamp, H.J. (2011). Somatostatin receptor 5 is palmitoylated by the interacting ZDHHC5 palmitoyltransferase. FEBS Lett. *585*, 2665–2670.

Korycka, J., Lach, A., Heger, E., Boguslawska, D.M., Wolny, M., Toporkiewicz, M., Augoff, K., Korzeniewski, J., and Sikorski, A.F. (2012). Human DHHC proteins: a spotlight on the hidden player of palmitoylation. Eur. J. Cell Biol. *91*, 107–117.

Koumandou, V.L., Klute, M.J., Herman, E.K., Nunez-Miguel, R., Dacks, J.B., and Field, M.C. (2011). Evolutionary reconstruction of the retromer complex and its function in Trypanosoma brucei. J. Cell Sci. *124*, 1496–1509.

Li, Y., Martin, B.R., Cravatt, B.F., and Hofmann, S.L. (2012). DHHC5 protein palmitoylates flotillin-2 and is rapidly degraded on induction of neuronal differentiation in cultured cells. J. Biol. Chem. *287*, 523–530.

Lipovsky, A., Popa, A., Pimienta, G., Wyler, M., Bhan, A., Kuruvilla, L., Guie, M.A., Poffenberger, A.C., Nelson, C.D., Atwood, W.J., and DiMaio, D. (2013). Genome-wide siRNA screen identifies the retromer as a cellular entry factor for human papillomavirus. Proc. Natl. Acad. Sci. USA *110*, 7452–7457.

Mallard, F., Tang, B.L., Galli, T., Tenza, D., Saint-Pol, A., Yue, X., Antony, C., Hong, W., Goud, B., and Johannes, L. (2002). Early/recycling endosomes-to-TGN transport involves two SNARE complexes and a Rab6 isoform. J. Cell Biol. *156*, 653–664.

McCormick, P.J., Dumaresq-Doiron, K., Pluviose, A.S., Pichette, V., Tosato, G., and Lefrancois, S. (2008). Palmitoylation controls recycling in lysosomal sorting and trafficking. Traffic *9*, 1984–1997.

McKenzie, J.E., Raisley, B., Zhou, X., Naslavsky, N., Taguchi, T., Caplan, S., and Sheff, D. (2012). Retromer guides STxB and CD8-M6PR from early to recycling endosomes, EHD1 guides STxB from recycling endosome to Golgi. Traffic *13*, 1140–1159.

Metcalf, D.J., Calvi, A.A., Seaman, M.Nj., Mitchison, H.M., and Cutler, D.F. (2008). Loss of the Batten disease gene CLN3 prevents exit from the TGN of the mannose 6-phosphate receptor. Traffic *9*, 1905–1914.

Naslavsky, N., McKenzie, J., Altan-Bonnet, N., Sheff, D., and Caplan, S. (2009). EHD3 regulates early-endosome-to-Golgi transport and preserves Golgi morphology. J. Cell Sci. *122*, 389–400.

Nielsen, M.S., Gustafsen, C., Madsen, P., Nyengaard, J.R., Hermey, G., Bakke, O., Mari, M., Schu, P., Pohlmann, R., Dennes, A., and Petersen, C.M. (2007). Sorting by the cytoplasmic domain of the amyloid precursor protein binding receptor SorLA. Mol. Cell. Biol. *27*, 6842–6851.

Ohno, Y., Kihara, A., Sano, T., and Igarashi, Y. (2006). Intracellular localization and tissue-specific distribution of human and yeast DHHC cysteine-rich domain-containing proteins. Biochim. Biophys. Acta *1761*, 474–483.

Pérez-Victoria, F.J., Mardones, G.A., and Bonifacino, J.S. (2008). Requirement of the human GARP complex for mannose 6-phosphate-receptor-dependent sorting of cathepsin D to lysosomes. Mol. Biol. Cell *19*, 2350–2362.

Popoff, V., Mardones, G.A., Tenza, D., Rojas, R., Lamaze, C., Bonifacino, J.S., Raposo, G., and Johannes, L. (2007). The retromer complex and clathrin define an early endosomal retrograde exit site. J. Cell Sci. *120*, 2022–2031.

Reggiori, F., and Tooze, S.A. (2012). Autophagy regulation through Atg9 traffic. J. Cell Biol. *198*, 151–153.

Riggs, K.A., Hasan, N., Humphrey, D., Raleigh, C., Nevitt, C., Corbin, D., and Hu, C. (2012). Regulation of integrin endocytic recycling and chemotactic cell migration by syntaxin 6 and VAMP3 interaction. J. Cell Sci. *125*, 3827–3839.

Rojas, R., van Vlijmen, T., Mardones, G.A., Prabhu, Y., Rojas, A.L., Mohammed, S., Heck, A.J., Raposo, G., van der Sluijs, P., and Bonifacino, J.S. (2008). Regulation of retromer recruitment to endosomes by sequential action of Rab5 and Rab7. J. Cell Biol. *183*, 513–526.

Rojas-Rivera, D., Armisén, R., Colombo, A., Martínez, G., Eguiguren, A.L., Díaz, A., Kiviluoto, S., Rodríguez, D., Patron, M., Rizzuto, R., et al. (2012). TMBIM3/GRINA is a novel unfolded protein response (UPR) target gene that controls apoptosis through the modulation of ER calcium homeostasis. Cell Death Differ. *19*, 1013–1026.

Seaman, M.N. (2004). Cargo-selective endosomal sorting for retrieval to the Golgi requires retromer. J. Cell Biol. *165*, 111–122.

Seaman, M.N. (2005). Recycle your receptors with retromer. Trends Cell Biol. *15*, 68–75.

Seaman, M.N. (2012). The retromer complex - endosomal protein recycling and beyond. J. Cell Sci. *125*, 4693–4702.

Seaman, M.N., McCaffery, J.M., and Emr, S.D. (1998). A membrane coat complex essential for endosome-to-Golgi retrograde transport in yeast. J. Cell Biol. *142*, 665–681.

Seaman, M.N., Harbour, M.E., Tattersall, D., Read, E., and Bright, N. (2009). Membrane recruitment of the cargo-selective retromer subcomplex is catalysed by the small GTPase Rab7 and inhibited by the Rab-GAP TBC1D5. J. Cell Sci. *122*, 2371–2382.

Seaman, M.N., Gautreau, A., and Billadeau, D.D. (2013). Retromer-mediated endosomal protein sorting: all WASHed up!. Trends Cell Biol. *23*, 522–528.

Small, S.A. (2008). Retromer sorting: a pathogenic pathway in late-onset Alzheimer disease. Arch. Neurol. *65*, 323–328.

Steinberg, F., Gallon, M., Winfield, M., Thomas, E.C., Bell, A.J., Heesom, K.J., Tavaré, J.M., and Cullen, P.J. (2013). A global analysis of SNX27-retromer assembly and cargo specificity reveals a function in glucose and metal ion transport. Nat. Cell Biol. *15*, 461–471.

Tai, G., Lu, L., Wang, T.L., Tang, B.L., Goud, B., Johannes, L., and Hong, W. (2004). Participation of the syntaxin 5/Ykt6/GS28/GS15 SNARE complex in transport from the early/recycling endosome to the trans-Golgi network. Mol. Biol. Cell *15*, 4011–4022.

Temkin, P., Lauffer, B., Jäger, S., Cimermancic, P., Krogan, N.J., and von Zastrow, M. (2011). SNX27 mediates retromer tubule entry and endosome-to-plasma membrane trafficking of signalling receptors. Nat. Cell Biol. *13*, 715–721.

Thomas, G.M., Hayashi, T., Chiu, S.L., Chen, C.M., and Huganir, R.L. (2012). Palmitoylation by DHHC5/8 targets GRIP1 to dendritic endosomes to regulate AMPA-R trafficking. Neuron *73*, 482–496.

Udayar, V., Buggia-Prévot, V., Guerreiro, R.L., Siegel, G., Rambabu, N., Soohoo, A.L., Ponnusamy, M., Siegenthaler, B., Bali, J., Simons, M., et al.; AESG (2013). A paired RNAi and RabGAP overexpression screen identifies Rab11 as a regulator of β-amyloid production. Cell Reports *5*, 1536–1551.

Vardarajan, B.N., Breusegem, S.Y., Harbour, M.E., Inzelberg, R., Friedland, R., St. George-Hyslop, P., Seaman, M.N., and Farrer, L.A. (2012). Identification of Alzheimer disease-associated variants in genes that regulate retromer function. Neurobiol. Aging *33*, 2231.e15–2231.e30.

Vilariño-Güell, C., Wider, C., Ross, O.A., Dachsel, J.C., Kachergus, J.M., Lincoln, S.J., Soto-Ortolaza, A.I., Cobb, S.A., Wilhoite, G.J., Bacon, J.A., et al. (2011). VPS35 mutations in Parkinson disease. Am. J. Hum. Genet. *89*, 162–167.

Wassmer, T., Attar, N., Bujny, M.V., Oakley, J., Traer, C.J., and Cullen, P.J. (2007). A loss-of-function screen reveals SNX5 and SNX6 as potential components of the mammalian retromer. J. Cell Sci. *120*, 45–54.

Wassmer, T., Attar, N., Harterink, M., van Weering, J.R., Traer, C.J., Oakley, J., Goud, B., Stephens, D.J., Verkade, P., Korswagen, H.C., and Cullen, P.J. (2009). The retromer coat complex coordinates endosomal sorting and dynein-mediated transport, with carrier recognition by the trans-Golgi network. Dev. Cell *17*, 110–122.

Willnow, T.E., and Andersen, O.M. (2013). Sorting receptor SORLA—a trafficking path to avoid Alzheimer disease. J. Cell Sci. *126*, 2751–2760.

Wu, C.C., Taylor, R.S., Lane, D.R., Ladinsky, M.S., Weisz, J.A., and Howell, K.E. (2000). GMx33: a novel family of trans-Golgi proteins identified by proteomics. Traffic *1*, 963–975.

Yamaji, T., Nishikawa, K., and Hanada, K. (2010). Transmembrane BAX inhibitor motif containing (TMBIM) family proteins perturbs a trans-Golgi network enzyme, Gb3 synthase, and reduces Gb3 biosynthesis. J. Biol. Chem. *285*, 35505–35518.

Yang, P.T., Lorenowicz, M.J., Silhankova, M., Coudreuse, D.Y., Betist, M.C., and Korswagen, H.C. (2008). Wnt signaling requires retromer-dependent recycling of MIG-14/Wntless in Wnt-producing cells. Dev. Cell *14*, 140–147.

Zech, T., Calaminus, S.D., Caswell, P., Spence, H.J., Carnell, M., Insall, R.H., Norman, J., and Machesky, L.M. (2011). The Arp2/3 activator WASH regulates $\alpha5\beta1$-integrin-mediated invasive migration. J. Cell Sci. *124*, 3753–3759.

Zhang, J., Reiling, C., Reinecke, J.B., Prislan, I., Marky, L.A., Sorgen, P.L., Naslavsky, N., and Caplan, S. (2012). Rabankyrin-5 interacts with EHD1 and Vps26 to regulate endocytic trafficking and retromer function. Traffic *13*, 745–757.

Zimprich, A., Benet-Pagès, A., Struhal, W., Graf, E., Eck, S.H., Offman, M.N., Haubenberger, D., Spielberger, S., Schulte, E.C., Lichtner, P., et al. (2011). A mutation in VPS35, encoding a subunit of the retromer complex, causes late-onset Parkinson disease. Am. J. Hum. Genet. *89*, 168–175.

Cell Reports, Volume *9*

Supplemental Information

Genome-wide RNAi Screen Reveals a Role for Multipass Membrane Proteins in Endosome-to-Golgi Retrieval

Sophia Y. Breusegem and Matthew N.J. Seaman

Supplemental Information

Genome-wide RNAi screen reveals role for multi-pass membrane proteins in endosome-to-Golgi retrieval

Sophia Y. Breusegem and Matthew N. J. Seaman

Inventory of Supplemental Information:

Supplemental Data:

Figure S1: ZDHHC5-Myc localizes to the plasma membrane, endosomes, and Rab8- and Rab11-positive recycling tubules – Related to Figure 4B.

Figure S2: ZDHHC5 KD differentially affects SNARE proteins – Related to Figure 4.

Figure S3: GRINA KD perturbs Golgi protein localization; GRINA-Myc expression coalesces retromer-positive endosomes – Related to Figure 5.

Figure S4: Further characterization of retromer and retromer cargo in SFT2D2, ZDHHC5 or GRINA KD cells – Related to Figure 6.

Table S1: TGN retrieval ratios measured in pilot screen of 310 trafficking genes – Related to Figure 1.

Table S2: Primary screen data for the 1087 primary screen hits – Related to Figure 2C.

Table S3: Validation screen results – Related to Figure 2E.

Table S4: Reported cellular locations and functional information for the 88 validated hits – Related to Figure 2F.

Supplemental Experimental Procedures

Supplemental References

Supplemental Data:

Supplemental Figures and Legends:

Figure S1: ZDHHC5-Myc localizes to the plasma membrane, endosomes, and Rab8- and Rab11-positive recycling tubules – Related to Figure 4B.

(A-B) Cells stably expressing mStrawberry-Rab8a were transfected with ZDHHC5-Myc for 24 hours before fixing and staining. Transfected cells demonstrate co-localization of ZDHHC5-Myc with Rab8-positive recycling tubules and with VPS35-positive endosomes (arrows in (**B**)). The area of (**A**) magnified in (**B**) is marked by a white box. **(C)** HeLa cells were transfected with ZDHHC5-Myc for 24 hours before fixation and staining for Myc (*red*) and MICAL-L1 (*green*). Arrows indicate co-localization of ZDHHC5 and MICAL-L1, an interactor of Rab8a (Sharma et al., 2009). **(D-E)** HeLa cells stably expressing GFP-EHD1 were transfected with ZDHHC5-Myc, fixed and stained for Myc (*red*), GFP (*green*) and VPS35 (*blue*). The boxed area in (**D**) is magnified in (**E**). Arrows indicate co-localization of ZDHHC5 and EHD1, another interactor of Rab8a (Roland et al., 2007), on recycling tubules. (**F**) Cells stably expressing GFP-Rab11 were transfected with ZDHHC5-Myc for 24 hours before fixing and staining. Transfected cells show co-localization of intracellular ZDHHC5-Myc with GFP-Rab11 and with MICAL-L1 (*white* colour in overlay image). However, some ZDHHC5-positive tubules are positive for MICAL-L1 but not GFP-Rab11 (*pink* colour in overlay image). Scale bars (**A**, **C**, **D**, **F**) 20 μm, (**B**, **E**) 5 μm.

Figure S2: ZDHHC5 KD differentially affects SNARE proteins – Related to Figure 4.

(A-E) Control and ZDHHC5-silenced HeLa cells were fixed and stained for CIMPR (**A-D**, *red*) or TGN46 (**E**, *red*), GM130 (*blue*, **A-D**) and, in *green*, STX6 (**A**), STX7 (**B**), STX8 (**C**), STX10 (**D**) or STX16 (**E**). **(F)** Whole cell lysates of control and ZDHHC5-silenced HeLa

cells were separated on LDS-PAGE and blotted for the indicated proteins. ZDHHC5 silencing did not affect the total cellular level for any of the SNARE proteins investigated. **(G)** Quantification of the average intensity of the staining for the indicated syntaxin proteins in a representative set of images as in (**A-E**). ZDHHC5 knockdown significantly increased the measured average intensity of STX6, STX7 and STX8 but not of STX5 (*no images shown*), STX10 or STX16. While some of these proteins could be substrates for the palmitoyl transferase activity of ZDHHC5 (e.g. STX7 and/or STX8 (He and Linder, 2009)), others might, similar to CIMPR, depend on ZDHHC5 for normal trafficking, and thus their localization is disrupted when ZDHHC5 is silenced. Scale bar (**A-E**): 20 μm.

Figure S3: GRINA KD perturbs Golgi protein localization; GRINA-Myc expression coalesces retromer-positive endosomes – Related to Figure 5.

(A-C) Control (*top rows*) and GRINA-silenced (*bottom rows*) cells were fixed and stained for (**A**): GM130 (*red*), Golgi mannosidase II (*green*) and VPS35 (*blue*); (**B**): EEA1 (*red*) and STX5 (*green*); (**C**): TGN46 (*red*) and LAMP1 (*green*). GRINA knock-down perturbs Golgi-targeting of Mannosidase II (**A**) and decreases Golgi-localized TNG46 (**C**). In addition, GM130 staining appears fragmented (**A**) while STX5 staining is unaffected (**B**). Finally, GRINA knock-down does not change endosomal morphology of EEA1 (**B**) or VPS35 (**A**) but results in an apparent increase in lysosome size (LAMP1 staining, **C**). **(D)** HeLa cells were transfected with GRINA-Myc and stained for Myc (*red*), SNX1 (*green*) and VPS35 (*blue*). The transfected cell is indicated by an asterisk. GRINA-Myc expression coalesces SNX1- and VPS35-positive endosomes into larger round structures that contain GRINA-Myc. Scale bars (**A-D**): 20 μm.

Figure S4: Further characterization of retromer and retromer cargo in SFT2D2, ZDHHC5 and GRINA KD cells – Related to Figure 6.

(A) SFT2D2, ZDHHC5 or GRINA KD do not affect retromer assembly or interactions with the WASH complex or with TBC1D5. Control and siRNA-treated HeLa cells stably expressing VPS29-GFP were lysed and VPS29-GFP-interacting proteins were isolated by co-immunoprecipitation (co-IP). Lysates and co-IP samples were analysed by Western blotting for the indicated proteins. **(B)** GRINA KD reduces retromer cargo stability. Control and KD cells, silenced for the indicated proteins, were either left untreated or incubated for 3 hours at 37ºC with 100 μg/mL cycloheximide before lysis. Total cell lysates were separated by LDS-PAGE and blotted for the indicated proteins. **(C)** VPS35 endosomes are brighter in SFT2D2, ZDHHC5 or GRINA KD cells compared to control cells. Multiple cells (> 500) were imaged using an automated microscope and measured fluorescence intensities were quantified. SFT2D2, ZDHHC5 or GRINA KD increase the brightness of the VPS35-positive endosomes, increasing their size to a much smaller extent. ** indicates $p < 0.01$ compared to control, * indicates $p < 0.05$ compared to control.

Supplemental Tables

Table S1: TGN retrieval ratios measured in pilot screen of 310 trafficking genes – Related to Figure 1.

Table S2: Primary screen data for the 1087 primary screen hits – Related to Figure 2C.

Table S3: Validation screen results – Related to Figure 2E.

Table S4: Reported cellular locations and functional information for the 88 validated hits – Related to Figure 2F.

Supplemental Experimental Procedures

Plasmids and Plasmid Transfection

The CD8-CIMPR reporter construct was described before (Seaman, 2004) and subcloned in pIRESpuro2 (Clontech, Saint-Germain-en-Laye, France). Human GOLPH3 was PCR-amplified, cloned into pEGFP-C1 (Clontech) and subcloned in pIRESNeo2 (Clontech). Single colonies of HeLa cells stably expressing both GFP-GOLPH3 and CD8-CIMPR were selected and screened for expression. A single cell line was used for all studies presented.

Transfection-ready plasmids encoding Myc- and Flag-tagged proteins (SFT2D2, ZDHHC5, GRINA) were from Origene (Rockville, MD, USA) and purchased from Cambridge Bioscience (Cambridge, UK). Plasmids were transfected into HeLa cells using Effectene (Qiagen, Manchester, UK) according to the manufacturer's instructions, or using polyethylenimine (PEI, Polysciences Inc., Warrington, PA, USA) according to (Breusegem and Seaman, 2014). To create a cell line stably expressing SFT2D2-Myc the gene was excised from the pCMV6 vector by digestion with SgfI and FseI and inserted into a similarly digested pIRESneo2 modified by insertion of a SgfI and FseI site between its NheI and BamHI sites. Restriction enzymes were from New England Biolabs (Hitchin, UK) and ligations were done using the Rapid Ligation kit (Roche Diagnostics Ltd, Burgess Hill, UK). Constructs were verified by agarose gel electrophoresis and immunofluorescence after transient transfection. Stable cell lines were selected using geneticin (Life Technologies Ltd, Paisley, UK).

Cell culture

Mouse hybridoma cells (American Tissue Culture Collection) for the production of monoclonal anti-CD8 antibody were grown in Excell medium (Sigma-Aldrich, Gillingham, UK) supplemented with 10 mM L-glutamine (Sigma-Aldrich).

All other cell lines were maintained in DMEM/high glucose medium containing 5% fetal bovine serum (FBS), 2 mM L-glutamine, 50 units/ml penicillin and 50 μg/ml streptomycin (all from Sigma-Aldrich) (complete medium). HeLa cells stably expressing GFP-GOLPH3 and CD8-CIMPR were grown in complete medium that additionally contained 0.4 mg/mL geneticin (Life Technologies Ltd) and 1 μg/ml puromycin (Sigma-Aldrich). HeLa cells stably expressing mStrawberry-Rab8a were a gift from Andrew Peden (University of Sheffield, UK) and maintained in complete medium containing 0.4 mg/mL geneticin. HeLa cells stably expressing GFP-EHD1 were described before (Gokool et al., 2007), as were HeLa cells stably expressing GFP-tagged Rab proteins (Seaman et al., 2009) and HeLa cells stably expressing VPS29-GFP (Collins et al., 2005). To eliminate possible mycoplasma contamination cells were treated with Plasmocin (Invivogen, San Diego, CA, USA) before use in screening.

Mini-library screens

Six mini-libraries of ON TARGETplus^{TM} siRNA pools (4 oligos/gene, Dharmacon, Thermo Fisher Scientific, Waltham, MA, USA) targeting genes homologous to yeast *VPS* proteins, SNARE protein genes, candidate endocytosis genes or kinesin, dynein or myosin motor protein genes were arrayed into the central wells of V-bottomed 96-well plates (Nunc, Thermo Fisher) and stored at -20°C until ready to use. On a day of screening plates containing 10 μL 1 μM siRNA pool (in siRNA buffer, Thermo Fisher) in the central wells were thawed to room temperature and spun at 5,000 x g for 5 minutes. SiRNA oligos

targeting the retromer proteins VPS26 and SNX1 (obtained from Dharmacon and described in (Gokool et al., 2007)) were added to selected outer wells of the plates as positive controls, while some wells only contained siRNA buffer. Oligofectamine (Life Technologies) was diluted 1:10 in Optimem (Life Technologies) and equilibrated at room temperature for 8 minutes. The siRNA pools were diluted by the addition of 36 μL Optimem to each well. Next 9 μL of the Oligofectamine dilution was added to each well, and siRNA:Oligofectamine complexes were allowed to form for 20 minutes at room temperature. Meanwhile HeLa cells stably expressing CD8-CIMPR and GFP-GOLPH3 were lifted off a 75 cm^2 flask and counted using a CASY cell counter (Roche, Basel, Switzerland). The siRNA:Oligofectamine complexes were aliquoted in 2 flat-bottomed assay plates (Corning, Amsterdam, The Netherlands), each containing 20 μL/well. Cells were diluted in complete medium to 60,000 cells/mL, and 100 μL of this cell suspension was added to each assay plate well. The plates were vortexed briefly and incubated at 37ºC for 72 hours.

Trafficking of CD8-CIMPR was then assessed using the anti-CD8 antibody uptake assay depicted in **Figure 1A**. The cell culture medium was flicked out of the plates and cells were washed once with room temperature PBS (100 μL/well) before incubation with anti-CD8 monoclonal antibody (hybridoma cell culture supernatant diluted 1:5 in complete cell culture medium, 40 μL/well) for 15 minutes at room temperature. Unbound antibody was removed by quickly washing the cells with 50 μL/well PBS and 100 μL/well pre-warmed (37°C) complete medium was then added for a 30-minute antibody chase at 37ºC. All subsequent steps were carried out at room temperature. At the end of the antibody chase period the cells were washed again with PBS before fixation with 50 μL/well 4% paraformaldehyde (PFA, Polysciences Inc., Warrington, PA, USA) in PBS for 10 minutes. Cells were permeabilized with 50 μL/well 0.1% TX-100 (Sigma-Aldrich) in PBS for 10 minutes. Unspecific binding of

antibodies was blocked by incubation for 30 minutes with 3% BSA (Fisher Scientific UK, Loughborough, UK) in PBS (IF buffer). Rabbit anti-GFP (described in (Seaman et al., 2009)) was added at 1:1,000 in IF buffer for 1 hour. Following 2 washes with PBS cells were incubated with Alexa Fluor 488 anti-rabbit antibody and Alexa Fluor 555 anti-mouse IgG_{2a} antibody (each diluted 1:2,000 in IF buffer) for 1 hour. Cells were washed twice with PBS before incubation for 30 minutes with Whole Cell Stain blue (Cellomics®, Thermo Fisher) diluted 1:1,000 in PBS. After 2 final washes with PBS cells were stored with 150 µL/well PBS at 4°C until imaging could be performed.

Cells were imaged on a Cellomics® Arrayscan V^{TI} automated microscope using the Colocalization Bio-application in the Arrayscan software. Per well at least 250 cells ("objects" defined by the whole cell stain) were imaged unless the "sparse well" criterion (8 consecutive field with less than 2 selected objects) was reached. The whole cell stain, Alexa Fluor 488 and Alexa Fluor 555 images were acquired sequentially using a single multi-pass filter set. Images were stored and further analysed in the Cellomics® vHCS^{TM}:View software. Regions of interest (ROIs) A and B as indicated in **Figure 1B** were defined in the Colocalization bio-application and derived from the GFP-GOLPH3 and whole cell stain images, respectively. The TGN retrieval ratio as defined in **Figure 1B** was directly calculated in the software for each cell and averaged over all cells selected in each well. Average TGN retrieval ratio values were imported into Origin software (OriginLab Corporation, Northampton, MA, USA) for further analysis and graphical presentation.

Each mini-library was screened at least 2 times in duplicate. From the replicate measurements 20 genes were selected whose knockdown reproducibly reduced the TGN retrieval ratio to values equal to or lower than the average TGN retrieval ratio measured for the SNX1 siRNA positive control. For these 20 genes the sequences making up the ON TARGETplus^{TM} siRNA

pool were ordered as individual ON TARGETplus^{TM} oligos (Dharmacon) and arrayed in columns 3 to 12 of a 96-well plate. Positive (SNX1 siRNA) and negative control wells were arrayed as in **Figure 2A**. Cells expressing GFP-GOLPH3 and CD8-CIMPR were reverse transfected with siRNA using the same protocol as for the ON TARGETplus^{TM} siRNA pool transfection. The anti-CD8 antibody uptake assay was also as described above and repeated twice in duplicate. Results of all mini-library screens are in **Table S1**.

Genome-wide siRNA screen

21,121 siRNA smartpools (siGenome, Dharmacon/Thermo Scientific) were arrayed onto 267 ninety-six-well plates and stored in 10 μL 1 μM aliquots at -20ºC. On a day of transfection siRNAs were thawed to room temperature and spun at 5,000 x g for 5 minutes. Cells stably expressing GFP-GOLPH3 and CD8-CIMPR were reverse transfected as in the mini-library screens, except that 8,000 cells were seeded per well and all steps involving 96-well plates were carried out using a robotic liquid handling system (BiomekNX, Beckman Coulter, High Wycombe, UK) enclosed in a in a custom-made enclosure with airflow technology and a UV lamp (BigNeat Containment Technology, Hampshire). Only cells that were passaged less than 10 times were used. Anti-CD8 antibody uptake was also carried out as described for the mini-library screens, using a bench-top semi-automated liquid dispenser system (Matrix Wellmate, Thermo Scientific) to aid in the antibody-uptake steps as well as in the fixing and staining steps.

Primary hit selection

Several acquired data as well as values calculated by the iView software were exported, assembled and linked to the plate layouts in a SQL database before being imported in Origin for statistical analysis and graphical presentation. TGN retrieval ratios were normalized plate-by-plate, such that the average TGN retrieval ratio for the negative control wells on each pate

equalled the average value measured across the entire screen (0.65). Hit selection was based on calculated SSMD values. SSMD values, defined in (Zhang, 2007), measure the magnitude of the difference between an siRNA of interest and a negative reference: $SSMD = \frac{\mu_i - \mu_N}{\sqrt{\sigma_i^2 + \sigma_N^2}}$, where μ_i and σ_i are the mean, respectively standard deviation, for an siRNA of interest and μ_N and σ_N the mean, respectively standard deviation for the negative control. We calculated SSMD values for the normalized TGN retrieval ratio as well as for the anti-CD8 intensity in the cytoplasm outside the GOLPH3-defined TGN mask. Primary hits were defined as having absolute values for these 2 SSMD values larger than 3 (i.e. very strong effects *vs*. the negative control), as well as fulfilling the following additional criteria: i) at least 40 cells were measured on each plate, with at least one measurement requiring less than 50 fields (i.e. siRNA is non-toxic and does not target a gene necessary for cell division); ii) the siRNA pool targets a single gene, taking into account re-annotation of the original siGenome pools by M. Boutros (DKFZ, using RefSeq25); and iii) for targets represented on an analysed microarray only detected ones were retained as hits. These criteria yielded 1106 primary hits, of which 1087 were still retained in the NCBI database.

To select hits for secondary screening an additional SSMD was calculated for the total cellular anti-CD8 signal to exclude siRNAs that cause a secretion defect (and therefore have less CD8-CIMPR at the plasma membrane at any one time) instead of an endosome-to-Golgi trafficking defect. This intensity criterion yielded 389 very strong hits which were visually inspected to assess morphology, anti-CD8 signal intensity distribution, cell polarization and cell size. In addition, manual inspection was also done for primary hits associated with the gene ontology terms intracellular trafficking, cytoskeleton, Golgi or GTPase activity. In this way 360 genes were selected for follow-up using ON TARGETplus^{TM} siRNA SMARTpools.

Table S2 assembles relevant data acquired for the 1087 primary screen hits as well as the 3 calculated SSMD values used in hit selection.

Validation screen

ON TARGETplus^{TM} siRNA SMARTpools in siRNA buffer were arrayed in the central wells of 96-well plates in 5 μL or 10 μL 1 μM aliquots and stored at -20ºC until use. Reverse transfection of cells with siRNA and anti-CD8 antibody uptake assay were as in the primary screen except that the ratio siRNA:Oligofectamine was decreased by ½ and additional negative (RISC-free and non-targeting) and positive (Rab7 and VPS26) siRNAs were included (see **Figure 2D**).

Small scale siRNA knockdown

For silencing of selected genes the cells of choice were seeded into 6-well plates to 30-40% confluency. Sixteen hours later cells were transfected with ON TARGETplus^{TM} siRNA SMARTpools, or, in the case of SNX1, a single ON TARGETplus^{TM} siRNA oligo. For each well to be transfected 10 μL Oligofectamine was diluted into 20 μL Optimem and left at r.t. for 5-10 minutes. 5 μL of a 20 μM siRNA stock solution was diluted in 165 μL Optimem. The diluted Oligofectamine solution was then added to the diluted siRNA solution and left for 20 minutes at r.t. Cells were washed once with Optimem. siRNA:Oligofectamine complexes were further diluted with 0.8 mL Optimem. The final 1 mL siRNA:Oligofectamine was added drop-wise to the cells. Four hours later 1 mL complete medium containing 20% FBS was added to each well. After 24 hours at 37ºC the medium was replaced with regular complete medium and cells were further incubated at 37ºC. Cells were assayed 72 hours after siRNA transfection. When required cells were trypsinised and seeded onto clean coverslips 48 hours after transfection.

Antibodies and Reagents

Anti-CD8 monoclonal antibody was obtained from hybridoma cell culture supernatant. Rabbit polyclonal antibodies against GFP and against human VPS26, TGN46 and SNX1 were generated in house and are described in previous publications (Seaman, 2004; Seaman et al., 2009). Rabbit polyclonal anti-SFT2D2 antibody was from Abcam (Cambridge, UK). Rabbit polyclonal anti-ZDHHC5 antibodies were obtained from Abcam, Sigma and Protein Tech Group Inc (Manchester, UK), with the Sigma antibody being used for immunofluorescence. Other antibodies used in immunofluorescence: monoclonal anti-GFP (Life Technologies), monoclonal anti-SNX1, anti-EEA1, anti-GM130 and anti-α5-integrin (all from BD Biosciences, Oxford, UK), rabbit polyclonal anti-Mannosidase II (Millipore/Chemicon, Watford, UK), rabbit polyclonal and mouse monoclonal anti-Myc, monoclonal anti-VPS35 and anti-LAMP1 (all from Santa Cruz Biotechnology Inc., Heidelberg, Germany), monoclonal anti-Flag (clone M2) and rabbit anti-GLG1 (both from Sigma), monoclonal anti-CIMPR and anti-β1-integrin and rabbit anti-STX16 (Abcam), rabbit anti-STX5 (Synaptic Systems, Goettingen, Germany), monoclonal anti-MICAL-L1 (Novus Biologicals, Cambridge, UK), rabbit anti-STX6, anti-STX7, anti-STX8, anti-STX10, anti-VAMP3, anti-VAMP7, anti-VAMP8, and mouse anti-STX16 (all generous gifts from Andrew Peden, University of Sheffield, UK). Other antibodies used for Western blotting: rabbit polyclonal anti-CIMPR (a generous gift from Paul Luzio, University of Cambridge, UK), monoclonal anti-transferrin receptor (Life Technologies), rabbit polyclonal anti-actin and monoclonal anti-tubulin (both from Sigma), rabbit anti-FAM21, rabbit anti-strumpellin and monoclonal anti-TBC1D5 (all from Santa Cruz Biotechnology Inc.). Alexa Fluor 488, Alexa Fluor 555 or Alexa Fluor 647 labelled anti-mouse and anti-rabbit antibodies were obtained from Life Technologies, including isotope-specific goat anti-mouse IgG_1, IgG_{2a} or IgG_{2b} antibodies. Horse radish peroxidase-conjugated antibodies were from Sigma.

Immunofluorescence

In preparation for immunofluorescent staining cells on 22 mm square coverslips in 6-well plates were washed once with PBS and fixed in 4% paraformaldehyde (Polysciences Inc.) in PBS for 10 minutes at room temperature. Cells were then permeabilized for 10 minutes in 0.1% TX-100 (Sigma) in PBS. Next, unspecific antibody binding was blocked by incubation for 20 minutes in IF buffer (see *Mini-library screens*). Coverslips were then incubated sequentially with the primary and secondary antibodies diluted in IF buffer at room temperature for 1 hour each, with three 5-minute washes with PBS in between. After 3 final washes with PBS the coverslips were mounted using Prolong Gold (Life Technologies). This permeabilization, blocking and staining protocol was followed for all antibodies except when staining for SNARE proteins, in which case each of these steps were done in PBS buffer containing 0.1% saponin (Sigma-Aldrich) and 5% fetal bovine serum. Immunolabeled cells were imaged at room temperature using a 63x oil immersion 1.4 N.A. objective on a ZeissAxiovert epifluorescence microscope equipped with a Hamamatsu ORCA-R2 CCD camera, using Simple PCI6 acquisition software. Images were processed in Zeiss LSM Browser software, using the range indicator display option to adjust background and displayed intensity range, and applying identical adjustments to all images to be compared. Some immunofluorescence images were acquired on a Zeiss Axioimager epifluorescence microscope equipped with an ORCA Flash 4 camera, using a 63x 1.4 N.A. oil immersion objective and the Zeiss ZenBlue software.

For the co-localization analysis in **Figure S2** images were opened in ImageJ software (National Institutes of Health) and Pearson's correlation coefficients calculated using the Just Another Colocalization Plugin (JACoP). Average intensity measurements were also made in ImageJ after applying a constant intensity threshold to all images to be analysed. For the co-localization and intensity analyses in **Figures 3**, **6** and **S4-C** cells were seeded and stained in

24-well plates and images were acquired on the Cellomics Arrayscan automated microscope using the co-localization bio-application. For each experimental condition at least 500 cells were imaged, and, when possible, technical replicates were included. Co-localization and intensity parameters were obtained from analysis in the Cellomics® vHCS™:View software. As in the anti-CD8 uptake assay, a whole cell stain was used to define the cellular outline.

Western Blotting

To prepare total cell lysates for Western blotting cells grown to confluency in 6-well plates were washed once with ice-cold PBS and then scraped in 100 μL/well 1% TX-100 in PBS. Alternatively, cell lysates were prepared as part of an immunoprecipitation or lectin pulldown protocol (*see below*). Lysates were reduced using DTT and denatured in LDS PAGE buffer (Life Technologies) at 85°C for 5 minutes before loading onto 4-12% NuPage Bis-Tris gels (Life Technologies). After electrophoretic separation at 150V for 70 minutes proteins were transferred onto nitrocellulose membranes. After overnight blocking with 5% milk in Tris-buffered saline buffer containing 0.1% Tween (TBST) membranes were probed with primary antibodies at room temperature for $1^{1/2}$ to 2 hours, followed by 3 washes with TBST/milk and incubation for 1 hour with HRP-conjugated secondary antibodies. After extensive washing of excess antibodies the proteins were visualized using luminescence detection reagents (GE Healthcare) and film.

Lectin Pull-Down

HeLa cells were seeded in 100 mm dishes at ~30-40% confluency. The next day cells were transfected with siRNA using Oligofectamine. For each dish 50 μL Oligofectamine was diluted with 100 μL Optimem and equilibrated for 5 minutes. 10 μL 20 μM siRNA was diluted in 850 μL Optimem before addition of 140 μL of the Optimem/Oligofectamine dilution. siRNA: Oligofectamine complexes were allowed to equilibrate for 20 minutes at r.t.

The siRNA: oligofectamine complexes were then diluted by the addition of 4 mL Optimem and added to the cells (after 1 wash of the cells with Optimem). After a 4 hour incubation at 37ºC 5 mL complete medium containing 20% FBS was added to each dish before further incubation at 37°C. Twenty-four hours later cells were trypsinized and seeded into 140 mm dishes. Seventy-two hours after the siRNA transfection the cells were washed once with PBS before incubation with 10 mL 0.1 mg/mL cycloheximide for 3 hours to halt new protein synthesis. Next, cells were washed once with ice-cold PBS and lysed in ice-cold lysis buffer (1% Triton X-100 in PBS containing 1 Complete™ protease inhibitor tablet (Roche) per 50 mL). Insoluble material was removed by centrifugation at 10,000 x g for 5 minutes at 4°C. Lysates were pre-cleared by rotating for 30 minutes at 4ºC in the presence of 50 μL sepharose-bound protein A. After removing the sepharose by centrifugation the lysates were incubated with 40 μL of a suspension of wheat germ agglutinin (WGA) conjugated to agarose (Sigma) for 2 hours at 4°C. The WGA-agarose beads were washed 4 x with lysis buffer and stored at -20°C until further processing. For analysis by Western blotting beads were vortexed in 1x LDS PAGE loading buffer before Western blotting as above.

Native Immunoprecipitation

HeLa cells stably expressing VPS29-GFP were seeded in 140 mm dishes and transfected at ~30-40% confluency with siRNA using Oligofectamine as described above for the siRNA transfections in the lectin pulldown experiments but using 2.5 x the amounts of siRNA, Oligofectamine and Optimem. After 24 hours cells were trypsinized and seeded into two 140 mm dishes. Seventy-two hours after siRNA transfection VPS29-GFP complexes were immunoprecipitated using a previously used lysis buffer and protocol, both recently detailed in (Breusegem and Seaman, 2014).

Supplemental References

Breusegem, S.Y., and Seaman, M.N. (2014). Image-based and biochemical assays to investigate endosomal protein sorting. Methods in enzymology *534*, 155-178.

Collins, B.M., Skinner, C.F., Watson, P.J., Seaman, M.N., and Owen, D.J. (2005). Vps29 has a phosphoesterase fold that acts as a protein interaction scaffold for retromer assembly. Nat Struct Mol Biol *12*, 594-602.

Gokool, S., Tattersall, D., and Seaman, M.N. (2007). EHD1 interacts with retromer to stabilize SNX1 tubules and facilitate endosome-to-Golgi retrieval. Traffic *8*, 1873-1886.

He, Y., and Linder, M.E. (2009). Differential palmitoylation of the endosomal SNAREs syntaxin 7 and syntaxin 8. J. Lipid Res. *50*, 398-404.

Roland, J.T., Kenworthy, A.K., Peranen, J., Caplan, S., and Goldenring, J.R. (2007). Myosin Vb interacts with Rab8a on a tubular network containing EHD1 and EHD3. Mol. Biol. Cell *18*, 2828-2837.

Seaman, M.N. (2004). Cargo-selective endosomal sorting for retrieval to the Golgi requires retromer. J Cell Biol *165*, 111-122.

Seaman, M.N., Harbour, M.E., Tattersall, D., Read, E., and Bright, N. (2009). Membrane recruitment of the cargo-selective retromer subcomplex is catalysed by the small GTPase Rab7 and inhibited by the Rab-GAP TBC1D5. J Cell Sci *122*, 2371-2382.

Sharma, M., Giridharan, S.S., Rahajeng, J., Naslavsky, N., and Caplan, S. (2009). MICAL-L1 links EHD1 to tubular recycling endosomes and regulates receptor recycling. Mol. Biol. Cell *20*, 5181-5194.

Zhang, X.D. (2007). A pair of new statistical parameters for quality control in RNA interference high-throughput screening assays. Genomics *89*, 552-561.

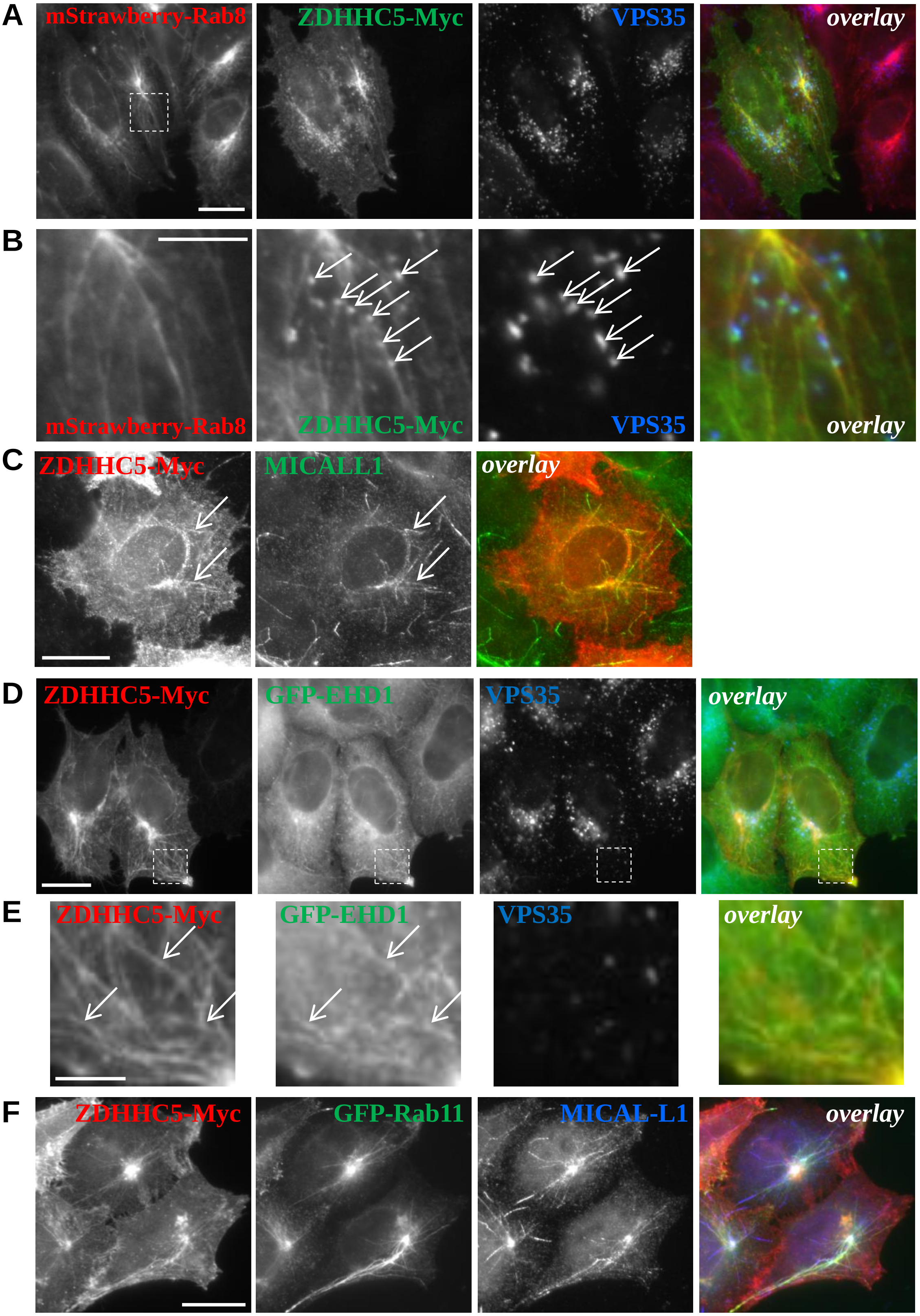
A
mStrawberry-Rab8
ZDHHC5-Myc
VPS35
overlay
B
mStrawberry-Rab8
ZDHHC5-Myc
VPS35
overlay
C
ZDHHC5-Myc
MICALL1
overlay
D
ZDHHC5-Myc
GFP-EHD1
VPS35
overlay
E
ZDHHC5-Myc
GFP-EHD1
VPS35
overlay
F
ZDHHC5-Myc
GFP-Rab11
MICAL-L1
overlay

Figure S1

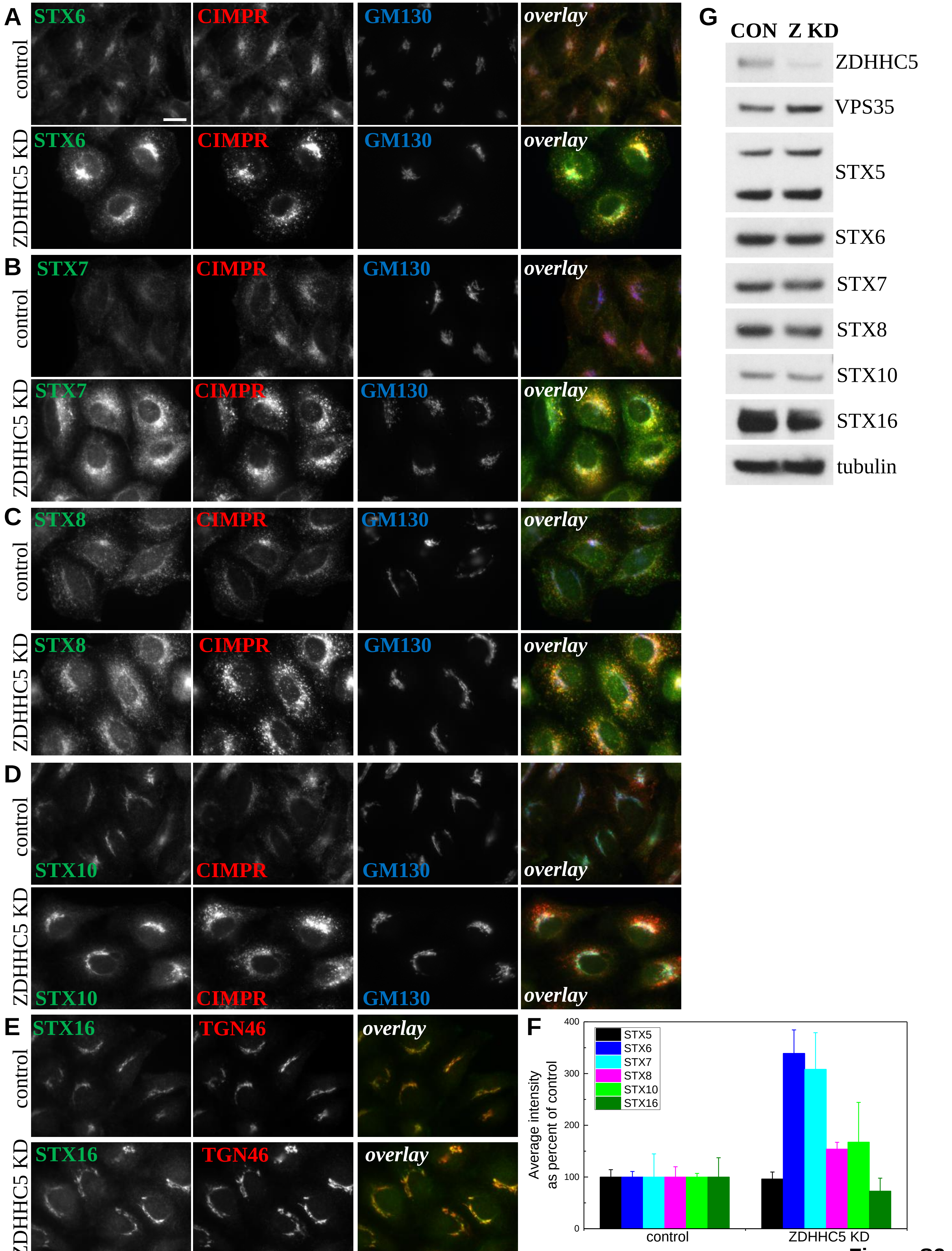

A
B
C
D
E
F
G
control
ZDHHC5 KD
STX6
STX7
STX8
STX10
STX16
CIMPR
GM130
TGN46
overlay
CON
Z KD
ZDHHC5
VPS35
STX5
STX6
STX7
STX8
STX10
STX16
tubulin
Average intensity as percent of control
0
100
200
300
400
STX5
STX6
STX7
STX8
STX10
STX16
control
ZDHHC5 KD

Figure S2

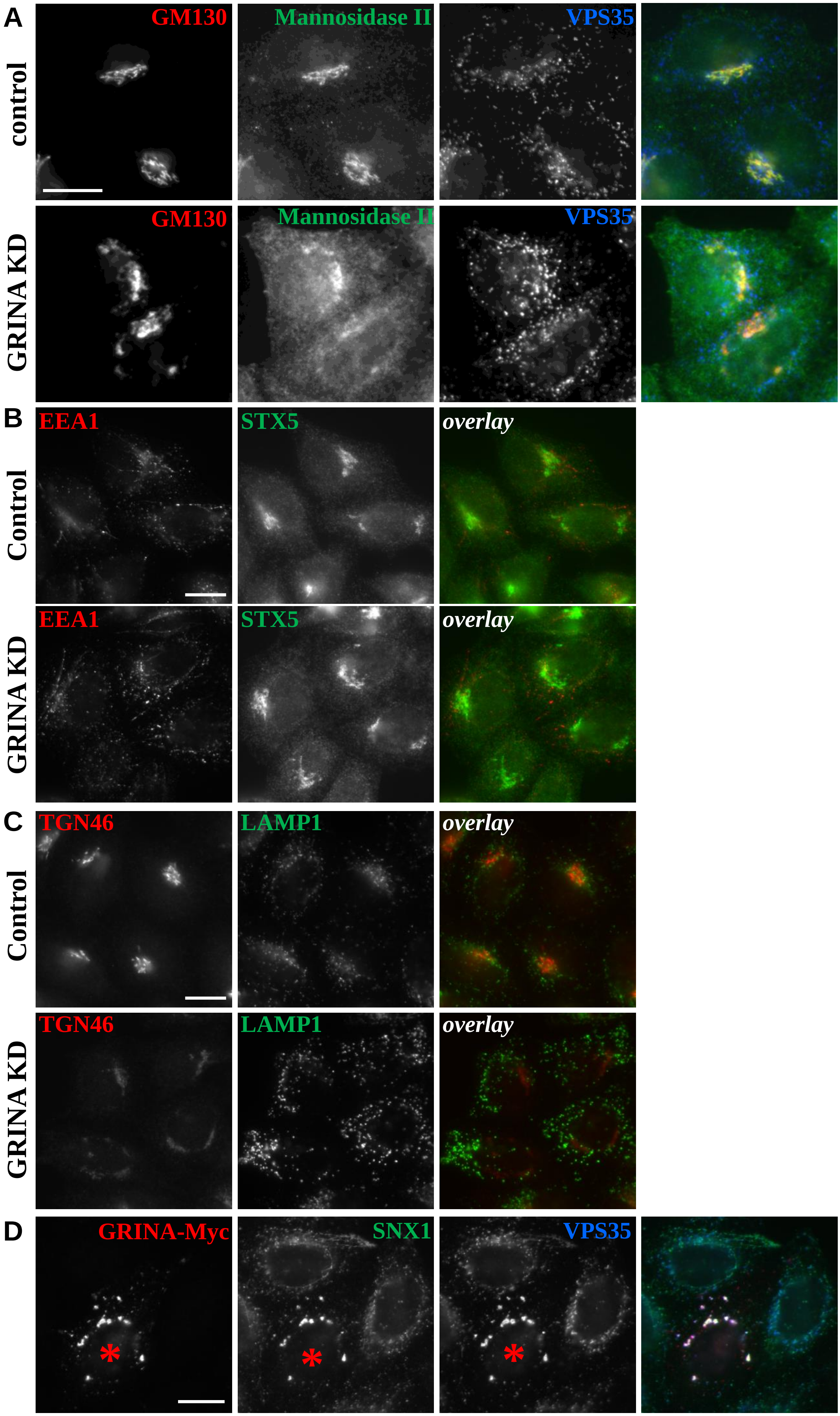
A
control
GM130
Mannosidase II
VPS35
GRINA KD
GM130
Mannosidase II
VPS35
B
Control
EEA1
STX5
overlay
GRINA KD
EEA1
STX5
overlay
C
Control
TGN46
LAMP1
overlay
GRINA KD
TGN46
LAMP1
overlay
D
GRINA-Myc
SNX1
VPS35

Figure S3

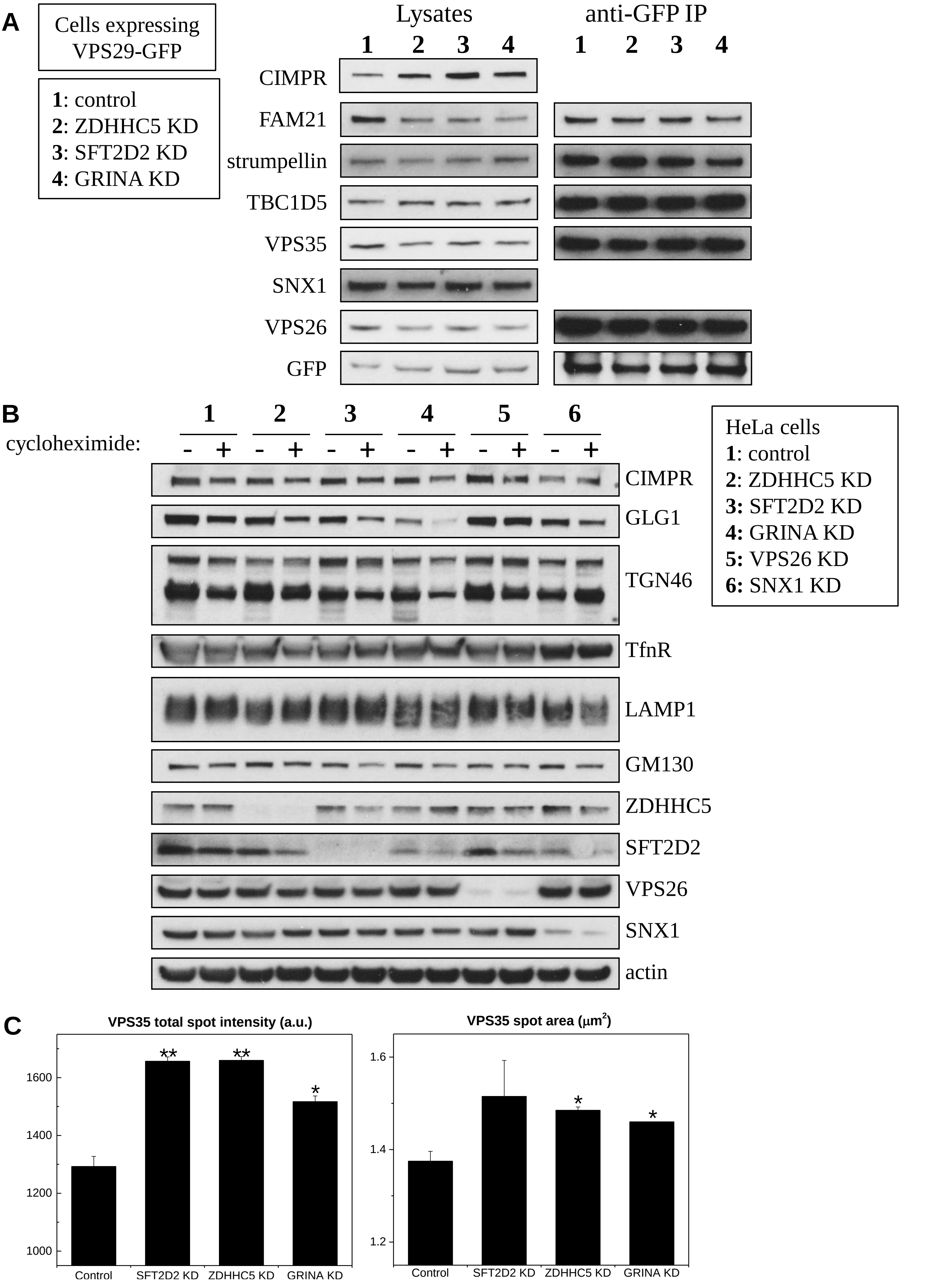
A
Cells expressing VPS29-GFP
1: control
2: ZDHHC5 KD
3: SFT2D2 KD
4: GRINA KD
Lysates
anti-GFP IP
1 2 3 4
1 2 3 4
CIMPR
FAM21
strumpellin
TBC1D5
VPS35
SNX1
VPS26
GFP
B
1 2 3 4 5 6
cycloheximide:
- + - + - + - + - + - +
CIMPR
GLG1
TGN46
TfnR
LAMP1
GM130
ZDHHC5
SFT2D2
VPS26
SNX1
actin
HeLa cells
1: control
2: ZDHHC5 KD
3: SFT2D2 KD
4: GRINA KD
5: VPS26 KD
6: SNX1 KD
C
VPS35 total spot intensity (a.u.)
1000
1200
1400
1600
**
**
*
Control
SFT2D2 KD
ZDHHC5 KD
GRINA KD
VPS35 spot area (μm^2)
1.2
1.4
1.6
*
*
Control
SFT2D2 KD
ZDHHC5 KD
GRINA KD

Figure S4
